# Supplementary material for: Scabies in the Amhara region of northern Ethiopia: a cross-sectional study of prevalence, determinants, clinical presentation and community knowledge
Source: BMJ Open. 2023 Oct 26;13(10):e075038. doi: 10.1136/bmjopen-2023-075038 (PMC10603513; doi:10.1136/bmjopen-2023-075038)
Supplement: Supplementary data [file bmjopen-2023-075038supp001.pdf]

Supplementary Materials

Yirgu, Middleton, Fekadu *et al.* Scabies in the Amhara region of northern Ethiopia: a cross-sectional study of prevalence, determinants, clinical presentation, and community knowledge. Submitted to *BMJ Global Health* April 2023.

Table of Contents

Figure S1. Study area and study locations

Table S1. Full questionnaire

Table S2. Fixed asset variables used to categorise households into wealth quintiles, with justifications

Figure S2. Study diagram, number of participants, and place of residence (*kebeles*)

Table S3. Distribution of participants by age and sex

Table S4. Scabies manifestations among clinically diagnosed cases

Table S5. Care-seeking characteristics of participants who sought care for scabies suggestive symptoms

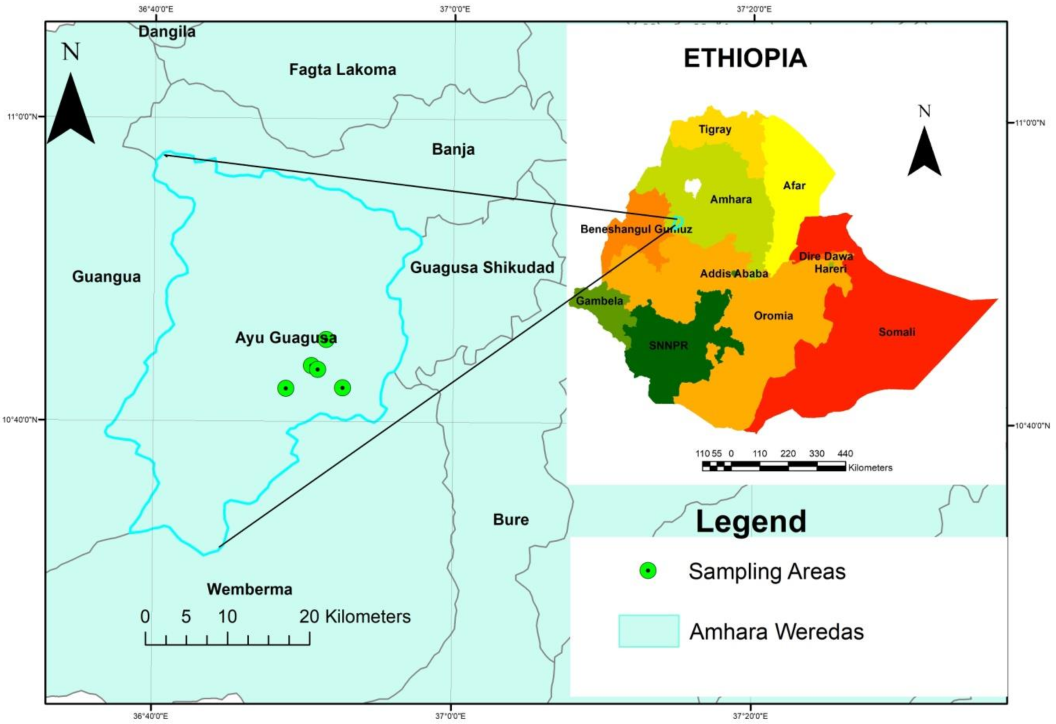

Figure S1. Study area and locations**:** Amhara Regional State; Ayu Guagusa district in Awi zone; sampled kebeles. Produced using ArcGIS version 10.8 (ESRI, Redlands, USA).

Table S1. Full questionnaire

Household Questionnaire

Questionnaire.ID		________________________				
**NO**	**QUESTIONS AND FILTERS**	**CODING CATEGORIES**				
**IDENTIFICATION**<br>**Please record the following identifying information prior to beginning the interview.**						
001	**Name of the interviewer**					
002	**Profession of the interviewer**					
003	**Date and time**	Date	Month	Day	Year	
004a	**Region**	Tigray......1<br>Afar......2<br>Amhara......3<br>Oromia......4<br>Ethiopia Somali......5<br>Benishangul Gumuz......6<br>SNNPR......7<br>Gambella......8<br>Harari......9<br>Addis Ababa......10<br>Dire Dawa......11				
004b	**Zone**					
004c	**District**					
004d	**Locality (Kebele) name**					

**Questionnaire.ID**		________________________	
**NO**	**QUESTIONS AND FILTERS**	**CODING CATEGORIES**	
004e	**Gote**		
005	**Household number**	Number ☐	
006	**Number of visits to the house hold?**	Number ☐	
007	**Respondents first name**		
008	**Relationship of the respondent to the study household?**	HH member…………………………..1<br>Neighbor………………………………2<br>A friend of the family…………………3<br>Community leader……………………4<br>Don’t know……………………………-88<br>No response………………………….-99	

Yirgu R, *et al. BMJ Open* 2023; 13:e075038. doi: 10.1136/bmjopen-2023-075038

**SECTION 1.1 – Household Roster**<br>**I am now going to ask you a series of questions about usual members of the household**								
**HH member ID**	**Relationship to head of the HH**	**Age at the last birth day**	**Gender**	**Education**	**Marital status**	**Occupation**	**Scabies symptoms**	**Referred for verification**
	101.What is (name) relationship to (name of the head of the HH)?<br>Head…………..1<br>Wife/Husband...2<br>Son/Daughter…3<br>Son/Daughter-in-law...4<br>Grandchild…..….5<br>Parent.………….6<br>Parent in law.….7<br>Brother/Sister…8<br>House help…….9<br>Other____________<br>Don't know……-88<br>No response.…-99	102.How old is (name)?	103.Is (name) male or female?<br><br>Male………1<br>Female……2	104.What is the highest level of school (name) attended? *Document the last completed grade.*<br>Never Attended .... 0<br>Primary .... 1<br>Secondary .... 2<br>Technical & vocational .... 3<br>Higher.................................. 4<br>No response……….-99	105.What is (name) marital status?<br><br>Married .... 1<br>Living with a partner................ 2<br>Divorced / separated............ 3<br>Widow / widower 4<br>Never Married.. .. 5<br>NA…………….6<br>Don't know…-88<br>No response . . . .-99	106.What is (name) occupation?<br><br>Farmer………1<br>Government employee……2<br>Daily laborer..3<br>Merchant……4<br>Student……...5<br>Unemployed...6<br>Notapplicable..7	107.Did (name) complain itchy skin rash/papules in the past two months?<br><br>Yes……….1<br>No………...2<br>No response…-99<br>Don't know-88	108. The participant is sent for verification?<br><br>Yes……….1<br>No………...2

CHAPTER - I - HOUSEHOLD CHARACTERISTICS

SECTION-1 HOUSEHOLD CHARACTERISTICS

Section 1. 2 – Household asset

Now I would like to ask you a few questions about the asset your household owns, this information helps us to assess the contribution of household wealth to the health status of members of the household

NO	QUESTIONS AND FILTERS	CODING CATEGORIES	Skip to:
109	**Please tell me about the items your household owns. Does your household have:**<br>*Read out all types and select all that apply.*<br>*If an item is reported broken but said to be out of use only temporarily, select the item. Otherwise do not select the item.*	Electricity .... 1/0<br>A watch/clock.. 1/0<br>A radio.. 1/0<br>A television .... 1/0<br>A mobile phone .... 1/0<br>A non-mobile telephone .... 1/0<br>A refrigerator.. 1/0<br>A table .... 1/0<br>A chair .... 1/0<br>A bed with cotton/sponge/spring mattress 1/0<br>An electric mitad .... 1/0<br>A kerosene lamp/pressure lamp................. 1/0<br>A bicycle .... 1/0<br>A motorcycle/ scooter .... 1/0<br>An animal-drawn cart................................ 1/0<br>A car/truck.. 1/0<br>None of the above.....................................-77<br>No response..………………………-99	
110a	**Does this household own any livestock, herds, other farm animals, or poultry?**<br>*These livestock can be kept anywhere, not necessarily on the homestead.*	Yes... 1<br>No .... 0<br>No response ....-99	0 → 111a

Yirgu R, *et al. BMJ Open* 2023; 13:e075038. doi: 10.1136/bmjopen-2023-075038

110b	**How many of the following animals does this household own?**<br>**Zero is a possible answer. Enter -88 for do not know. Enter -99 for no response.**<br>*The household can keep the livestock anywhere but must own the livestock recorded here.*	Milk cows/bulls/oxen [ ]<br>Horses/donkeys/mules [ ]<br>Camels [ ]<br>Goats [ ]<br>Sheep [ ]<br>Chickens [ ]<br>Beehives [ ]	
110c	**Are any of the livestock, herds, other farm animals, or poultry kept in the homestead?**	Yes..……………………………………1<br>No..……………………………………..2<br>Don't know..…………………………-88<br>No response..……………………….-99	0 → 111a
110d	**Which animals are kept in the homestead?**<br>*Only refer to those animals which were mentioned in 109c*	Milk cows/bulls/oxen ………………1<br>Horses/donkeys/mules……...............2<br>Camels………………………………3<br>Goats………………………………..4<br>Sheep……………………………….5<br>Chickens……………………………6<br>Don't know…………………………-88<br>No response……………………….-99	
**The next questions are about the source of water your household regularly use for different purposes.**			

111a	**Which of the following water sources does your household use on a regular basis for any part of the year for any purpose?**<br>*Read out all types and check all that are used.*	Piped Water<br>Piped into dwelling/indoor.. .... 1/0<br>Pipe to yard/plot…………... ... 1/0<br>Public tap/standpipe ………1/0<br>Tube well or borehole ... ……………1/0<br>Dug Well<br>Protected Well ... ……………..1/0<br>Unprotected Well . ……………1/0<br>Water from Spring<br>Protected Spring .... …………..1/0<br>Unprotected Spring .. ………….1/0<br>Rainwater ………………………….1/0<br>Tanker Truck ………………………1/0<br>Cart or Bicycle with Small Tank .. …1/0<br>Surface water<br>(River / Dam / Lake / Pond / Stream<br>/ Canal / Irrigation Channel)... ……..1/0<br>Bottled Water ……………………...1/0<br>Sachet Water . ………………………1/0<br>No Response________________________-99	

111b	**What is the main source of water used by your household to take shower?**<br>*Read out Q110a selections only.*	Piped Water<br>Piped into dwelling/indoor.. .... 1/0<br>Pipe to yard/plot…………... ... 1/0<br>Public tap/standpipe ……….1/0<br>Tube well or borehole .. …………….1/0<br>Dug Well<br>Protected Well . ………………1/0<br>Unprotected Well …………….1/0<br>Water from Spring<br>Protected Spring .. ……………1/0<br>Unprotected Spring . …………..1/0<br>Rainwater …………………………..1/0<br>Tanker Truck ……………………….1/0<br>Cart or Bicycle with Small Tank . ….1/0<br>Surface water<br>(River / Dam / Lake / Pond / Stream<br>/ Canal / Irrigation Channel)... ……..1/0<br>Bottled Water ……………………...1/0<br>Sachet Water . ………………………1/0<br>No Response________________________-99	

111c	**What is the main source of water used by your household to wash cloths?**<br>*Read out 110b selections only.*	Piped Water<br>Piped into dwelling/indoor.. .... 1/0<br>Pipe to yard/plot…………... ... 1/0<br>Public tap/standpipe ………1/0<br>Tube well or borehole ... ……………1/0<br>Dug Well<br>Protected Well ... ……………..1/0<br>Unprotected Well . ……………1/0<br>Water from Spring<br>Protected Spring .... …………..1/0<br>Unprotected Spring .. ………….1/0<br>Rainwater ………………………….1/0<br>Tanker Truck ………………………1/0<br>Cart or Bicycle with Small Tank .. …1/0<br>Surface water<br>(River / Dam / Lake / Pond / Stream<br>/ Canal / Irrigation Channel)... ……..1/0<br>Bottled Water ……………………...1/0<br>Sachet Water . ………………………1/0<br>No Response………………………-99	
111d	**How long does it take to go to [MAIN WATER SOURCE]get water, and come back?**<br>*Zero is a possible answer*<br>*Enter -88 for do not know*<br>*Enter -99 for no response*<br><br>*Report the time into minutes.*<br>*Answer includes waiting time in line.*	Minutes: [ ]	

<table>
<tr><td colspan="4"><u>SECTION-1 HOUSEHOLD CHARACTERISTICS</u>
<u>Section 1. 3 – Household Observation</u>
To fill the next part of the questionnaire I need to actually observe what materials you have used to build the floor, wall and roof of your house. I also wish to take a look at the place where members of the household wash their hands. Is it ok if I take a walk with you in the compound and do the observations?</td></tr>
<tr><td>112</td><td>Main material of the floor
Observe.</td><td>Earth/Sand 1
Dung 2
Wood Planks...3
Palm/Bamboo 4
Polished wood 5
Asphalt strips 6
Ceramic Tiles 7
Cement 8
Carpet 9
Other________________________
Don't know…………………………-88</td><td></td></tr>
<tr><td>113</td><td>Main material of the roof
Observe.</td><td>No Roof 1
Thatch/Leaf/ Mud...2
Rustic Mat/Plastic Sheets 3
Bamboo 4
Wood Planks...5
Cardboard 6
Corrugated Iron/Metal 7
Asbestos/Cement Fiber...............................8
Cement/Concrete 9
Roof Shingles 10
Others________________________
Don't know…………………………-88</td><td></td></tr>
</table>

114	**Main material of the exterior walls**<br>*Observe.*	No Walls...1<br>Trunks/Bamboo ....2<br>Dirt.…………………………………..3<br>Wood with Mud..4<br>Stone with Mud ....5<br>Uncovered Adobe ....6<br>Plywood..7<br>Cardboard ....8<br>Reused Wood ....9<br>Corrugated sheets..………………10<br>Cement ....11<br>Stone with Lime/Cement..........................12<br>Bricks ....13<br>Cement Blocks ....14<br>Covered Adobe...15<br>Wood Planks/Shingles..............................16<br>Others________________________<br>Don't know…………………………-88	
115a	**We would like to learn about the places that households use to wash their hands. Can you please show me where members of your household most often wash their hands?**	Observed, fixed place ....………………1<br>Observed, mobile.......................................2<br>Not observed, not in yard/plot....................3<br>Not observed, no permission to see............4<br>Not observed, other reason.........................5<br>No response .... -99	
115b	**At the place where the household washes their hands, observe if:**<br>*Check all that apply.*	Soap is present..1/0<br>Stored water is present ....1/0<br>Running water is present ....1/0<br>None of the above.................................. -77	

SECTION – 2 SCABIES AT A HOUSEHOLD LEVEL

Now I am going to ask you about skin complaints, whether you have seen or heard any of your household members complaining [small or medium fluid containing or solid itchy or non-itchy rashes with a burrow connecting the bumps and mainly affecting the finger webs, the wrist, back of the knee, around the ankle or beneath the breast or the buttock].

[→ Show picture set no 1]

201	**Have you seen or heard any of your household members complaining [***Repeat the manifestations indicated in the preamble***]?**	Yes……………………………………1<br>No……………………………………..0<br>Don't know…………………………-88<br>No response……………………….-99	0 → 205
202	**How many of the household members exhibited [***Repeat the manifestations indicated in the preamble***]?**	Number_______	
203	**Which members of the household presented [***Repeat the manifestations indicated in the preamble***]?**<br>*Take name/code of members of the household from section 1(Household roster)*	1.<br>2.<br>3.<br>4.<br>5.	
204	**What signs have you observed on their skin [** Show → picture set no 2]**?**<br>*This question is about any of the household members*<br>*More than one answer is possible*	Vesicles.……………………………….1<br>Papules ……………………………….2<br>Pustules ..……………………………..3<br>Bumps.……………………………….4<br>Scabies burrows.……………………..5<br>Crust.…………………………………...6<br>Scratch.………………………………..7<br>Others________________________<br>Don't know.………………………….-88<br>No response.………………………..-99	

Next, I will ask you few questions about health service availability and utilization			
205	**What is the nearest health facility to your home?**	Health post………………………….1<br>Health center……………………….2<br>Private clinic………………………..3<br>Hospital..…………………………....4<br>Don't know………………………-88<br>No response…………………….-99	
206	**Where do you usually go if you are sick, or to treat a general health problem?**	Health post………………………….1<br>Health center……………………….2<br>Private clinic………………………..3<br>Hospital..…………………………....4<br>Traditional healers…………………5<br>Holly water………………………….6<br>Prayer……………………………….7<br>Don't go anywhere…………………8<br>Others______________________<br>Don't know………………………..-88<br>No response……………………...-99	
207	**How far do you live from the** (*The facility selected in Q.no.205*)**?**<br>*The response could be recorded either in KMS or the time it takes to get to the facility, walking on foot.*<br>*The time should be filled in minutes*	In KMS ☐<br>Time it takes if walked on foot ☐	

208	**How do you travel to (*The facility selected in Q.no.205*)?**	On foot……………………………1<br>On a horse back……………………2<br>On a chariot………………………..3<br>By car……………………………...4<br>By other motorized vehicles……….5<br>Others____________________<br>Don't know………………………-88<br>No response..…………………….-99	

CHAPTER-2

INDIVIDUAL QUESTIONNAIRE FOR MEMBERS OF THE SELECTED HOUSEHOLD

HH number: ___________

Name of HH head :____________

Code HH member :____________

SECTION 3 – SCABIES MANIFESTATIONS ON INDIVIDUAL PARTICIPANTS

I am going to ask you if you have skin complaints, such as itch and rash, we ask these questions to all participants in order to understand the occurrence of the skin condition that we are interested to know about. For some of the questions you can simply tell me whether you have the symptoms, but for some I will ask you to show me parts of your body affected by the condition. I hope it is ok, but if you feel uncomfortable to show me your body you can tell me and we can skip the part which asks to show me your skin.

NB: All members of the household will be asked questions 301-310

301	**Relationship of the respondent with the participant of the study?**<br>*This question will be asked only if the participant is a minor*	Parent………………………………..1<br>Care taker……………………………2<br>Relative………………………………3<br>Others______________________<br>Don't know…………………………-88<br>No response………………………..-99	
302	**Do you feel itch on any part of your body?**	Yes……………………………………1<br>No……………………………………..0<br>Don't know…………………………-88<br>No response……………………….-99	0 → 305
303	**How do you grade the itch?**	Mild…………………………………1<br>Moderate……………………………..2<br>Intense………………………………..3<br>Don't know…………………………-88<br>No response..……………………….-99	

304	**On which part of your body do you mainly feel the itch?**<br>*More than one response is possible*<br>*Please help the respondent use the body map*	The wrist………………………………1<br>Inter digital space…………………….2<br>The elbow……………………………..3<br>Abdomen………………………………4<br>Back of the knee……………………...5<br>Ankle area……………………………..6<br>The head………………………………7<br>The neck area……..…………………..8<br>Genital area…………………………...9<br>The buttock……………………………10<br>Others________________________<br>Don’t know………………………….-88<br>No response………………………..-99	
305	**Do you have a skin rash on any part of your body?**	Yes……………………………………1<br>No……………………………………..0<br>Don’t know…………………………-88<br>No response..……………………….-99	0 → 401
306	**Is the rash associated with an itchy sensation?**	Yes……………………………………1<br>No……………………………………..0<br>Don’t know…………………………-88<br>No response..……………………….-99	

<table>
<tr><td>307</td><td>Which part of your body does the rash mainly involve?
More than one response is possible</td><td colspan="3">The wrist………………………………1
Inter digital space.…………………….2
The elbow……………………………..3
Abdomen………………………………4
Back of the knee.……………………...5
Ankle area……………………………..6
The head………………………………7
The neck area.……..………………….8
Genital area…………………………...9
The buttock..…………………………10
Others________________________
Don’t know………………………….-88
No response..………………………..-99</td><td></td></tr>
<tr><td rowspan="5">308</td><td rowspan="5">Can you show me parts of your body affected by the skin rash?
Observe and select parts of the body affected by the rash
More than one body part can be checked</td><td></td><td>Yes</td><td>No</td><td rowspan="5"></td></tr>
<tr><td>The wrist
Inter digital space
The elbow
Abdomen
Back of the knee
Ankle area
The head
The neck area</td><td></td><td></td></tr>
<tr><td colspan="3">The below two body parts could be documented based on history</td></tr>
<tr><td>Genital area
The buttock</td><td></td><td></td></tr>
<tr><td colspan="3">Others________________________
Don’t know………………………….-88
No response………………………..-99</td></tr>
</table>

<table>
<tr><td>309</td><td>Characterize the rash you have observed on the respondent?
This question is for the data collector</td><td>Vesicles……………………………….1
Papules ……………………………….2
Pustules ..….………………………..3
Bumps…………………………………4
Scabies burrows……………………….5
Crust…………………………………...6
Scratch.………………………………..7
Others________________________
Don’t know………………………….-88
No response..………………………..-99</td><td></td></tr>
<tr><td>310</td><td>How many lesions were observed on the patient.</td><td>≤10……………………………………1
10-50………………………………….2
≥ 50…………………………………..3</td><td></td></tr>
<tr><td colspan="4"><u>SECTION 4 – SCABIES TRANSMISSION</u>
The skin condition which we are interested to know about has manifestations like; [small or medium fluid containing or solid itchy or non-itchy rashes with a burrow connecting the bumps mainly affecting the finger webs, on the wrist, back of the knee, around the ankle or beneath the breast or the buttock]. Since one of the major mode of transmission of this condition is a close physical contact with the infected person, knowing all about the possibilities which invites close physical contact is pretty important for us to understand the disease transmission.
The physical contact should be [In the past two months]
[→ Show picture no 1]
NB: Only adults will respond to questions 401-403</td></tr>
<tr><td>401</td><td>Do you know anyone presenting the symptoms [Read the manifestations indicated in the preamble]?</td><td>Yes……………………………………1
No...…………………………………..0
No response..……………………….-99
Don’t know…………………………-88</td><td>0 → 501</td></tr>
<tr><td colspan="4">When answering questions 402a-c please think of three people, with the mentioned manifestations, whom are closer to you in comparison to the others with manifestations. We will refer to these people as person 1-3.</td></tr>
</table>

402a	**How do you know person 1?**	Friend……………………………......1<br>Neighbour…………………………….2<br>Business associate…………………....3<br>From the market place..……………...4<br>From school………………………….5<br>On a public/private transport...……....6<br>Spouse…………………………….....7<br>Sexual partner..………………..........8<br>Other members of the household……9<br>Others_____________________<br>No response……………………….-99<br>Don’t know……………………….-88	
402b	**How do you know person 2?**	Friend……………………………......1<br>Neighbor……………………………..2<br>Business associate…………………....3<br>From the market place..……………...4<br>From school………………………….5<br>On a public/private transport..……....6<br>Spouse…………………………….....7<br>Sexual partner..………………..........8<br>Other members of the household……9<br>Others_____________________<br>No response……………………….-99<br>Don’t know………………………….-88	

Yirgu R, *et al. BMJ Open* 2023; 13:e075038. doi: 10.1136/bmjopen-2023-075038

402c	**How do you know person 3?**	Friend……………………………......1<br>Neighbor……………………………..2<br>Business associate…………………...3<br>From the market place..……………...4<br>From school………………………….5<br>On a public/private transport...……....6<br>Spouse……………………………....7<br>Sexual partner..……………….........8<br>Other members of the household……9<br>Others________________________<br>No response..……………………….-99<br>Don't know…………………………-88	
When answering questions **403a-c**, please think of the type of contact you had with each one of the three people, you have referred to in questions 402a-c.			
403a	**Which one of the listed encounters did you have with person 1?**<br>*The encounter should be a minimum of one time.*<br>*More than one answer is possible.*<br>*Categories **7 and 8** will only be mentioned as possible responses if the reported contacts are spouses, sexual partners or household members.*	Sat together...…………….………...1<br>Shook hands with....…………………2<br>Dine together...….……………………3<br>Shared work related materials….…….4<br>Shared utensils….……………………5<br>Shared cloths…..…………………....6<br>*Shared a bed/resting space… …...…...7*<br>*Took shower together.….………………8*<br>Others______________________<br>Don't know…..…………………….-88<br>No response..…...…………………..-99	

403b	**Which one of the listed encounters did you have with person 2?**<br>*The encounter should be a minimum of one time.*<br>*More than one answer is possible.*<br>*Categories **7 and 8** will only be mentioned as possible responses if the reported contacts are spouses, sexual partners or household members.*	Sat together...……………….………...1<br>Shook hands with....…………………2<br>Dine together...….………………………3<br>Shared work related materials..………4<br>Shared utensils….……………………5<br>Shared cloths…...………………….....6<br>*Shared a bed/resting space…..........7*<br>*Took shower together….………….....8*<br>Others______________________<br>Don't know…...……………………….-88<br>No response…...…………………..-99	
403c	**Which one of the listed encounters did you have with person 3?**<br>*The encounter should be a minimum of one time.*<br>*More than one answer is possible.*<br>*Categories **7 and 8** will only be mentioned as possible responses if the reported contacts are spouses, sexual partners or household members.*	Sat together...……………….………...1<br>Shook hands with....…………………2<br>Dine together...….………………………3<br>Shared work related materials..………4<br>Shared utensils….……………………5<br>Shared cloths…...………………….....6<br>*Shared a bed/resting space…..........7*<br>*Took shower together….………….....8*<br>Others______________________<br>Don't know…...……………………….-88<br>No response..…...…………………..-99	

SECTION-5 SUMMARY OF THE REPORTED MANIFESTATIONS

This part summarizes the reported symptoms and observed signs on the skin of the participant. This table is to be filled only by the provider, and at the end of this section the HEW will report her impression of the participants condition.

	Main criteria	Yes	No	Not sure	
501	Scabies burrows				
502	Typical lesions affecting male genitalia				
503	Typical lesions in a typical distribution				

504	Atypical lesions or atypical distribution				
	History features				
505	Itch				
506	Close contact with an individual who has itch or typical lesions in a typical distribution				
507	**What is the impression of the health extension worker based on the above information?**	Clinical scabies………………………1<br>Suspected scabies…………………..2<br>Other skin diseases…………………3<br>Others________________________<br>Don't know………………………….-88<br>No response………………………..-99			
508	**Remarks on the diagnosis from the provider**				

<u>SECTION-6 HEATH SEEKING</u>

Based on the symptoms you told me before I think you might probably have an infestation called scabies and we will reach at a confirmatory diagnosis after we discuss with the skin doctors at the health center. But up until then I would like to ask you few questions about your condition.

NB: Only participants with clinical scabies will be asked questions 601-606

601	**What was the first manifestation of the disease?**	Itch……………………………………1<br>Rash………………………………….2<br>Papules………………………………3<br>Nodules..…..…………………………4<br>Ulcer………………………………….5<br>Crust………………………………….6<br>Don't know…………………………-88<br>No response……………………….-99	
602	**For how long did you stay with the disease counting from the day you experienced the [**The response to Q.no 601]?<br>*Assist the respondent to remember the day when he/she had the first manifestation*	Number of days ☐	

603	**Have you done anything to treat the symptoms?**	Yes………………………………1<br>No………………………………..0<br>Don't know………………………-88<br>No response…………………….-99	0 → 701
604	**What did you do for the first time to get rid of the symptoms?**<br>*This question pertains to both modern and traditional health care services*	Home remedy………………………1<br>Traditional medicine……………….2<br>Holly water………………………….3<br>Prayer……………………………….4<br>Health care from a health post……...5<br>Health care from a health center……6<br>Health care from private clinic.……7<br>Health care from hospital………….8<br>Others______________________<br>Don't know………………………..-88<br>No response…..…………………...-99	If 1,2,3,4 → 701
605	**How many days after the onset of the first symptom did you visit** [Health care facility from Q.no.604]**?**	Number of days ☐	

606	**Why didn't you visit** [The facility from Q.no.604] **earlier?**	Not sure where to go……………..……1<br>The symptoms were not serious……2<br>Could recover on its own…………..…3<br>I didn't have time…………………….4<br>Fear of stigma.……………………...5<br>Didn't think there is a medicine for it……………………………………..6<br>Distance/lack of transport to go to the health facility……......................................7<br>Fear of high treatment cost.………..8<br>Others_______________________<br>Don't know……………………….-88<br>No response…………………........-99	
**SECTION- 7- DERMATOLOGIC QUALITY OF LIFE QUESTIONS**<br>**The following questions are intended to measure the effect of your skin condition on your overall quality of life. The responses are organized in a way which reflects the degree of severity of your skin condition on the indicated aspect of your life. So before you give your responses please take a moment to rate how severely it has affected you. The responses range from *not at all to very much*, please select the response which describes your condition appropriately.**<br>*NB: Questions 701-708 will only be directed to adult (>18 years) participants.*			
701	Over the last week, how itchy, sore, painful or stinging has your skin been?	Very much.…………………………..4<br>A lot………………………………….3<br>A little.……………………………….2<br>Not at all..……………………………1	
702	Over the last week, how embarrassed or self conscious have you been because of your skin?	Very much.…………………………..4<br>A lot………………………………….3<br>A little.……………………………….2<br>Not at all..……………………………1	
703	Over the last week, how much has your skin interfered with you going shopping or looking after your home or garden?	Very much.…………………………..4<br>A lot………………………………….3<br>A little.……………………………….2<br>Not at all..……………………………1	

704	Over the last week, how much has your skin influenced the clothes you wear?	Very much……………………………..4<br>A lot………………………………….3<br>A little……………………………….2<br>Not at all..……………………………1	
705	Over the last week, how much has your skin affected any social or leisure activities?	Very much……………………………..4<br>A lot………………………………….3<br>A little……………………………….2<br>Not at all..……………………………1	
706	over the last week how much has your skin been a problem at work or studying?	Very much……………………………..4<br>A lot………………………………….3<br>A little……………………………….2<br>Not at all..……………………………1	
707	Over the last week, how much has your skin created problems with your partner or any of your close friends or relatives	Very much……………………………..4<br>A lot………………………………….3<br>A little……………………………….2<br>Not at all..……………………………1	
708	Over the last week, how much has your skin caused any sexual difficulties?	Very much……………………………..4<br>A lot………………………………….3<br>A little……………………………….2<br>Not at all..……………………………1	
**SECTION-8 KNOWLEDGE ABOUT SCABIES**<br>**I will ask you few questions about your knowledge about scabies. You will just tell me what you know about the disease, you are expected to tell me what you know about the disease. It doesn't need to be the right answer.**<br>**NB: Questions 801-810 are for adult (>18 years of age) study participants.**			
801	**Have you ever heard about scabies?**	Yes…………………………………1<br>No…………………………………..0<br>Don't know………………………-88<br>No response…………………….-99	0 → 1001

802	**Where did you first learn about scabies?**<br>*Check all that are mentioned*	Newspapers and magazines……………..1<br>Radio……………………………………2<br>TV………………………………………3<br>Billboards………………………………4<br>Brochures, posters and other printed materials………………………………..5<br>Health extension workers………………6<br>Health workers…………………………7<br>Family, friends, neighbors and colleagues………………………………8<br>Religious leaders……………………….9<br>Teachers………………………………10<br>Others_______________________<br>Don't know………………………….-88<br>No response……………………........-99	
803	**What are the signs and symptoms of scabies?**<br>*Check all that are mentioned*<br>*Don't read the choices to the respondent*	Itch…………………………………1<br>Rash/papules……………………..2<br>Ulceration of the skin…………….3<br>Skin crust…………………………4<br>Others____________________<br>Don't know………………………..-88<br>No response……………………...-99	

804	**How can a person get scabies?**<br>*Check all that are mentioned*<br>*Don't read the choices to the respondent*	Prolonged physical contact with a patient…..…………………….……….1<br>Sharing clothes with the infected person……..…………………………2<br>Sharing bedding with the infected person…...……………………………3<br>Through droplets when coughing….4<br>Through blood contact………………5<br>Due to evil spirit………………………6<br>From the soil…………………………7<br>From contaminated water…………..8<br>Others______________________<br>Don't know..………………………..-88<br>No response..……………………...-99	
805	**Can a person with scabies be cured?**	Yes…………………………………1<br>No…………………………………..0<br>Don't know………………………..-88<br>No response……………………...-99	
806	**In your opinion who can catch scabies?**<br>*Check all that are mentioned*<br>*Don't read the choices to the respondent*	Anybody……………………………..1<br>Poor people…………………………2<br>Rich people………………………….3<br>Homeless people…………………...4<br>People from the city………………...5<br>Farmers……………………………...6<br>Commercial sex workers…………..7<br>Prisoners……………………………8<br>Others_______________________<br>Don't know………………………..-88<br>No response……………………...-99	

807	**How can someone with scabies get cured?**	With therapeutic cream or tablets…..1<br>With traditional healers………………2<br>With holly water……………………….3<br>With prayer…………………………….4<br>Others______________________<br>Don't know………………………….-88<br>No response……………………......-99	
808	**Do you feel you are well informed about scabies?**	Yes…………………………………1<br>No…………………………………..0<br>Don't know………………………..-88<br>No response……………………...-99	
809	**Do you wish to get more information about scabies?**	Yes…………………………………1<br>No…………………………………..0<br>Don't know………………………..-88<br>No response……………………...-99	

Yirgu R, *et al. BMJ Open* 2023; 13:e075038. doi: 10.1136/bmjopen-2023-075038

810	**Which medium can best reach people like you, who are in need of information on scabies?**	Newspapers and magazines………1<br>Radio…………………………………2<br>TV………………………………….3<br>Billboards……………………………4<br>Brochures, posters and other printed materials……………………………..5<br>Health extension workers…………6<br>Health workers………………………7<br>Family, friends, neighbors and colleagues……………………………8<br>Religious leaders……………………9<br>Teachers……………………………..10<br>Other________________________<br>Don't know………………………..-88<br>No response……………………...-99	
**SECTION-9 ATTITUDE TOWARDS SCABIES**<br>**The following few questions are intended to assess your attitude towards scabies**<br>***NB: Questions 801- 807 will be asked for scabies free adults (Aged $\geq$ 18 years)***			
901	**Do you think you can get scabies?**	Yes…………………………………1<br>No…………………………………..2<br>Don't know………………………..-88<br>No response……………………...-99	
902a	**Why do you think you can catch scabies?**	Reason______________________________<br>________________________	
902b	**Why do you think you don't catch scabies?**	Reason______________________________<br>________________________	

903	**What would you feel if you found out that you have scabies?**<br>*Check all that are mentioned*<br>*Don't read the choices to the respondent*	Nothing……………………………1<br>Fear…………………………………..2<br>Shame………………………………..3<br>Surprise………………………………4<br>Sadness………………………………5<br>Others________________________<br>Don't know………………………..-88<br>No response……………………...-99	
904a	**What would you do if you think you had scabies?**	Go to a health facility………………..1<br>Wait till it goes away on its own……2<br>Seek self-treatment options………...3<br>Go to traditional healers…………….4<br>Go to a pharmacy……………………5<br>Others________________________<br>Don't know………………………..-88<br>No response……………………...-99	
904b	**Why wouldn't you want to go to a health facility?**<br>*Check all that are mentioned*<br>*Don't read the choices to the respondent*	Not sure where to go…………………1<br>The symptoms were not serious……2<br>Could recover on its own…………….3<br>I didn't have time……………………..4<br>Fear of stigma………………………...5<br>Didn't think there is a medicine for it..6<br>Distance/lack of transport to go to the health facility....................................7<br>Fear of high treatment cost….……....8<br>Others________________________<br>Don't know………………………….-88<br>No response……………………......-99	

905	**How expensive do you think the care for scabies at health care facilities?**<br>*Check only one response*	Reasonable price……………………1<br>Moderately expensive..……………...2<br>Very expensive………………………3<br>Free of charge………………………..4<br>Don't know………………………….-88<br>No response…………………......-99	
906a	**Do you know people who have/had scabies?**	Yes……………………………………1<br>No……………………………………..2	
906b	**Which of the following statements best describe your feeling towards scabies patients?**<br>*Check only one response*	I don't feel anything……………………1<br>It is their problem I don't get scabies..2<br>I feel compassion and want to help....3<br>I feel compassion but prefer to stay away……………………………………4<br>I fear they may infect me……………5<br>Others________________________<br>Don't know………………………….-88<br>No response…………………......-99	
907	**In your community how does a person with scabies get treated?**<br>*Check only one response*	Most people prefer to reject him/her..1<br>People appear friendly but they prefer to reject him/her………………………2<br>They get help/support………………..3<br>Others________________________<br>Don't know………………………….-88<br>No response…………………......-99	
**SECTION 10 – PERSONAL HYGIENE AND ONCHO MDA RELATED QUESTIONS**<br>**Now I would like to ask you a few questions about water, sanitation and hygiene**<br>***NB: All participants will be asked questions 1001-1010.***			

Yirgu R, et al. BMJ Open 2023; 13:e075038. doi: 10.1136/bmjopen-2023-075038

1001	**How often do you take shower?**	Number of times:<br>In a week ___<br>In a month ___<br>In a year ___	
1002	**Do you use soap or other detergents when you take shower?**	Yes, always…………………………1<br>Yes, sometimes..…………………..2<br>No…………………………………....3<br>Others________________________<br>No response……………………….-88	
1003	**How often do you wash your cloths?**	In a week ___<br>In a month ___<br>In a year ___	
1004	**Do you use soap or other detergents when you wash your cloths?**	Yes, always…………………………1<br>Yes, sometimes..…………………..2<br>No.……………………………………3<br>Others________________________<br>No response……………………….-88	
**Questions 1005-1010 are about onchocerchiasis MDA given in your kebele, so you will answer the following questions based on your past experience.**			
1005	**Have you ever received a medication to treat or prevent onchocerciasis?**	Yes….………………………………1<br>No ………………………………..0<br>Don't know…..……………………-88<br>No response….…………………..-99	0 → END
1006	**How many times have you received a medication to treat or prevent onchocerciasis?**	Number ☐	
1007	**When was the last time you received a medication to treat or prevent onchocerciasis?**	Day___Month____Year_____	

1008	**Did you take the medication you received the last time?**	Yes……………………………………1<br>No……………………………………..0<br>Don't know…………………………-88<br>No response…..…………………..-99	1→1010
1009	**Why didn't you take the medication?**	It makes me itch……………………..1<br>Not good for my health.……………..2<br>I was fasting…………………………..3<br>My friends/family told me not to…….4<br>Others________________________<br>Don't know…………………………-88<br>No response…..…………………..-99	
1010	**Where did you get the medication from?**	Health center…………………………1<br>Hospital……………………………….2<br>HEWs…………………………………3<br>HDA…………………………………...4<br>Others________________________<br>Don't know…………………………-88<br>No response……………………….-99	
**END**	**I have completed the interview thank you for your time.**		

Table S2. Fixed asset variables used to categorise households into wealth quintiles.

Household asset and infrastructure related variables
Does your house have electricity?
Does your household own a clock?
Does your household own a radio?
Does your household own a television?
Does any member of your household own a mobile phone?
Does your household own a refrigerator?
Does your household own a table?
Does your household own chair?
Does your household own bed with cotton or sponge mattress?
Does your household own an electric hot-plate?
Does your household own a kerosene lamp?
Does any member of your household own a bicycle?
Does any member of your household own a motorcycle?
Does your household own animal drawn cart?
Does your household own a car?
Does your household own farm animals?
Does your household own piped water into the dwelling?
Does your household own piped water into the yard?
Does your household have access to public tap?
Does your household own a dug well?
Does your household use spring water?
Does your household use rainwater?
Does your household use water distributed using tanker trucks?
Is the floor of your house made of sand or earth or sand?
Is the floor of your house made of ceramic tiles?

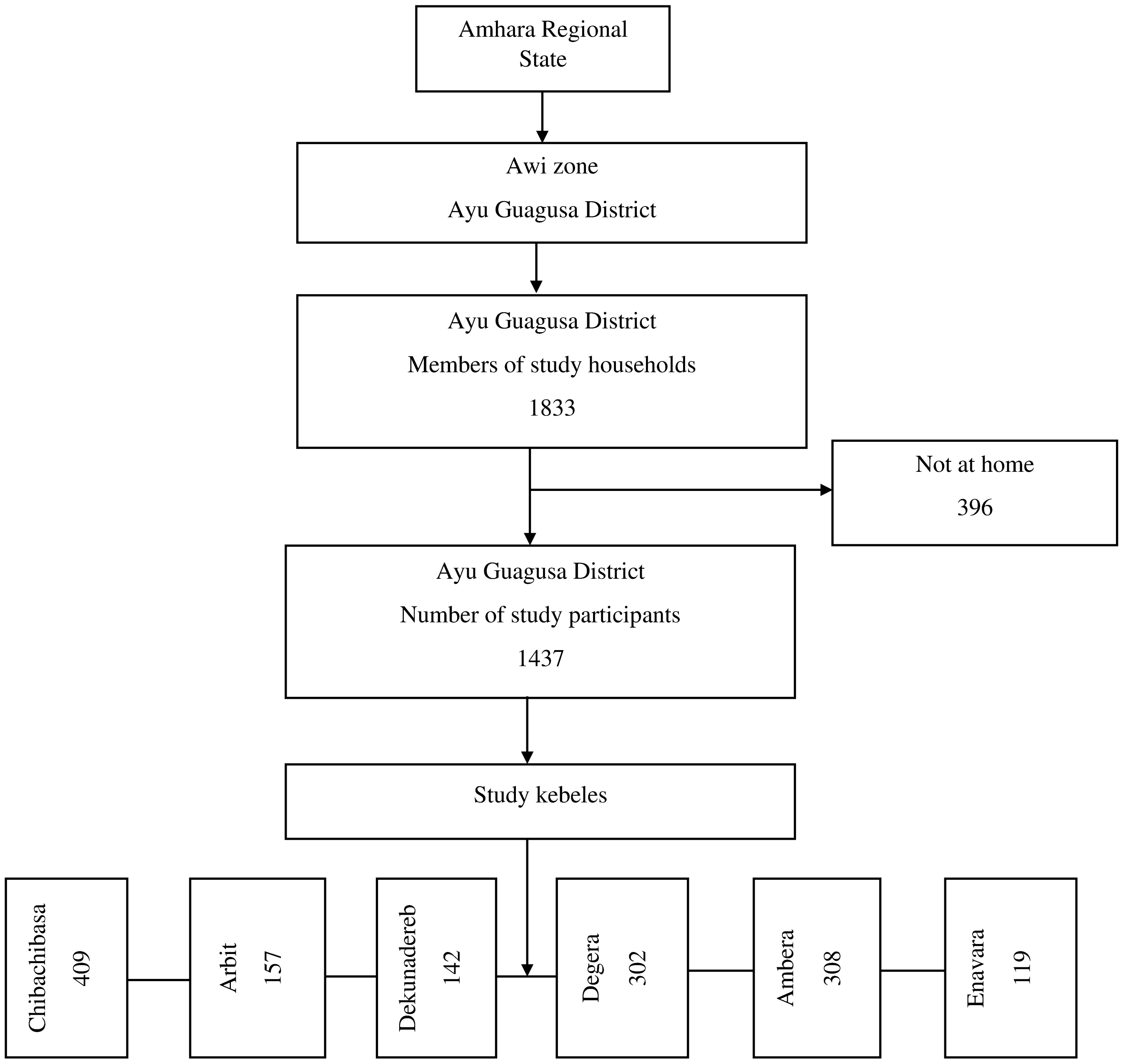

Figure S2. Study diagram, number of participants, and place of residence (*kebeles*)

Table S3 Distribution of participants by age and sex (n=1437)

Sex	Age categories	Total (%) (n=1437)	No scabies (%) (n=1245)	Clinical scabies (%) (n=192)
**Male**	0–10	173 (27.0)	148 (27.5)	25 (24.8)
	11–18	170 (26.6)	134 (24.9)	36 (35.6)
	19–40	165 (25.8)	144 (26.7)	21 (20.8)
	>41	132 (20.6)	113 (20.9)	19 (18.8)
**Female**	0–10	192 (24.1)	168 (23.8)	24 (26.4)
	11–18	176 (22.1)	153 (21.7)	23 (25.3)
	19–40	270 (33.9)	243 (34.4)	27 (29.7)
	>41	159 (19.9)	142 (20.1)	17 (18.7)

Table S4 Scabies manifestations among clinically diagnosed cases (n=192)

Variable	Categories	n (%) *
**Skin lesions**	Vesicles	130 (67.7)
	Scratch marks	117 (60.9)
	Papules	99 (51.6)
	Skin crust	78 (40.6)
	Pustule	52 (27.1)
	Burrows	8 (4.2)
**Skin rash distribution**	Trunk	119 (62.0)
	Elbow	99 (51.6)
	Finger webs	98 (51.0)
	Wrist	81 (42.2)
	Knee	38 (19.8)
	Neck	25 (13.0)
	Ankle	12 (6.3)
	Head	7 (3.6)
**History features**	Itch	184 (95.8)
	Contact history with a presumed scabies patient	184 (95.8)
**Relationship of participant with participant presumed source of infestation (n=184) [#]**	Household members	146 (79.3)
	Neighbour	20 (10.9)
	Friend	15 (8.2)

*Cumulative scores for some variables were more than the number of participants as the items were multi select.

[#] Calculated out of the number of participants who reported contact history with a person exhibiting signs of scabies.

Yirgu R, *et al. BMJ Open* 2023; 13:e075038. doi: 10.1136/bmjopen-2023-075038

Table S5 Care seeking characteristics of participants who sought care for scabies suggestive symptoms (n=62)

Variable	Categories	n (%)
**Source of care**	Health facility	40 (64.5)
	Self-treatment	14 (22.6)
	Holy water	3 (4.8)
	Prayer	1 (1.6)
	Missing values	4 (6.5)
**Time to care seeking**	Median number of days (IQR)	20 (7–30)
