# Supplementary material for: Scabies in the Amhara region of northern Ethiopia: a cross-sectional study of prevalence, determinants, clinical presentation and community knowledge
Source: BMJ Open. 2023 Oct 26;13(10):e075038. doi: 10.1136/bmjopen-2023-075038 (PMC10603513; doi:10.1136/bmjopen-2023-075038)
Supplement: Author's manuscript [file bmjopen-2023-075038.draft_revisions.pdf]

BMJ Open

BMJ Open is committed to open peer review. As part of this commitment we make the peer review history of every article we publish publicly available.

When an article is published we post the peer reviewers' comments and the authors' responses online. We also post the versions of the paper that were used during peer review. These are the versions that the peer review comments apply to.

The versions of the paper that follow are the versions that were submitted during the peer review process. They are not the versions of record or the final published versions. They should not be cited or distributed as the published version of this manuscript.

BMJ Open is an open access journal and the full, final, typeset and author-corrected version of record of the manuscript is available on our site with no access controls, subscription charges or pay-per-view fees (http://bmjopen.bmj.com).

If you have any questions on BMJ Open's open peer review process please email info.bmjopen@bmj.com

BMJ Open

Scabies in the Amhara region of northern Ethiopia: a cross-sectional study of prevalence, determinants, clinical presentation, and community knowledge

Journal:	*BMJ Open*
Manuscript ID	bmjopen-2023-075038
Article Type:	Original research
Date Submitted by the Author:	24-Apr-2023
Complete List of Authors:	Yirgu Belachew, Robel; Addis Ababa University College of Health Sciences, Public Health; Brighton and Sussex Medical School,<br>Middleton, Jo; Brighton and Sussex Medical School,<br>Fekadu, Abebaw; Addis Ababa University, Centre for Innovative Drug Development and Therapeutic Trials for Africa (CDT-Africa); Brighton and Sussex Medical School, Department of Global Health & Infection<br>Cassell, Jackie; Brighton and Sussex Medical School<br>Tesfaye, Abraham; Therapeutic Trials for Africa (CDT-Africa) CDT-Africa<br>Jones, Christopher; Brighton and Sussex Medical School<br>Bremner, Stephen; University of Sussex, Department of Primary Care and Public Health<br>Enbiale, Wendemagegn; Bahir Dar University College of Medical and Health Sciences<br>Davey, Gail; University of Sussex, Wellcome Trust Brighton and Sussex Centre for Global Health Research, Brighton and Sussex Medical School, Brighton
Keywords:	Epidemiology < TROPICAL MEDICINE, Infectious diseases & infestations < DERMATOLOGY, Public health < INFECTIOUS DISEASES, Neglected Diseases

SCHOLARONE™
Manuscripts

I, the Submitting Author has the right to grant and does grant on behalf of all authors of the Work (as defined in the below author licence), an exclusive licence and/or a non-exclusive licence for contributions from authors who are: i) UK Crown employees; ii) where BMJ has agreed a CC-BY licence shall apply, and/or iii) in accordance with the terms applicable for US Federal Government officers or employees acting as part of their official duties; on a worldwide, perpetual, irrevocable, royalty-free basis to BMJ Publishing Group Ltd ("BMJ") its licensees and where the relevant Journal is co-owned by BMJ to the co-owners of the Journal, to publish the Work in this journal and any other BMJ products and to exploit all rights, as set out in our licence.

The Submitting Author accepts and understands that any supply made under these terms is made by BMJ to the Submitting Author unless you are acting as an employee on behalf of your employer or a postgraduate student of an affiliated institution which is paying any applicable article publishing charge ("APC") for Open Access articles. Where the Submitting Author wishes to make the Work available on an Open Access basis (and intends to pay the relevant APC), the terms of reuse of such Open Access shall be governed by a Creative Commons licence – details of these licences and which Creative Commons licence will apply to this Work are set out in our licence referred to above.

Other than as permitted in any relevant BMJ Author's Self Archiving Policies, I confirm this Work has not been accepted for publication elsewhere, is not being considered for publication elsewhere and does not duplicate material already published. I confirm all authors consent to publication of this Work and authorise the granting of this licence.

For peer review only

Scabies in the Amhara region of northern Ethiopia: a cross-sectional study of prevalence, determinants, clinical presentation, and community knowledge

Robel Yirgu[1,2], Jo Middleton[1,3*], Abebaw Fekadu[1,4], Jackie A Cassell[1,3], Abraham Tesfaye[4], Christopher Iain Jones[3], Stephen Bremner[1,3], Wendemagegn Enbiale[5], Gail Davey[1,2]

[1]NIHR Global Health Research Unit on Neglected Tropical Diseases, Brighton and Sussex Medical School, Falmer, UK. [2]School of Public Health, Addis Ababa University, Addis Ababa, Ethiopia. [3]Primary Care and Public Health, Brighton and Sussex Medical School, Falmer, UK. [4]Center for Innovative Drug Development and Therapeutic Trials for Africa, Addis Ababa University, Addis Ababa, Ethiopia. [5]Bahir Dar University, Bahir Dar, Ethiopia.

*Correspondence: Robel Yirgu - yirgurob@gmail.com

Keywords: Scabies, Ethiopia, Neglected Tropical Diseases, *Sarcoptes scabiei*

For peer review only - http://bmjopen.bmj.com/site/about/guidelines.xhtml

ABSTRACT [294/300]

Introduction

The World Health Organization aims to prevent, eliminate, or control Neglected Tropical Diseases (NTDs), including scabies, by 2030. However, limited epidemiological data presented a challenge to control efforts, especially in high burden countries. There was a major scabies outbreak in northern Ethiopia starting in 2015 and prevalence has since increased across much of the country. This study aimed to measure scabies prevalence and presentation, identify its predictors, and assess community perception of, and knowledge about, the infestation.

Methods

This population-based cross-sectional study was conducted in Ayu Guagusa district, Amhara region, northern Ethiopia. 1437 people who were members of 381 randomly selected households participated in the study. Five trained mid-level health workers diagnosed scabies cases clinically. Participants were asked about their socioeconomic characteristics, knowledge and attitudes about scabies, and health seeking behaviours. Multi-level logistic regression models were fitted to adjust for individual and household-level confounding variables, and identify predictors of scabies infestation.

Results

Scabies prevalence was 13.4% (95% CI 11.8 - 15.5). Households of more than five people (adjusted Odds Ratio (aOR)=3.5, 95% CI 1.2 - 10.2) were associated with increased odds of developing scabies, however, females had lower odds (aOR=0.5 95% CI 0.3 - 0.8). Scabietic lesions most frequently involved the trunk (62.0%), and vesicles were the commonest types of lesions (67.7%). Two-thirds of adult study participants had heard about scabies and most obtained scabies related information from informal sources. Only 32% of cases sought care for scabies from any source.

Conclusions

Scabies prevalence was high, signifying the need for community-based control interventions. Host density and sex were important predictors of scabies. Despite the favourable attitude toward the effectiveness of scabies treatment, health care seeking was low.

For peer review only - http://bmjopen.bmj.com/site/about/guidelines.xhtml

What is already known on this topic

- A major scabies outbreak has been underway in northern Ethiopia since 2015. However, knowledge of prevalence, presentation, and predictors is mostly based on data primarily collected to guide control interventions or from medical records of health-facilities, not communities, and little is known about community knowledge and attitudes.

What this study adds

- In Ayu Guagusa district scabies prevalence was 13.4% (above the recommended threshold for mass drug administrations) large household size was associated with increased odds of developing scabies, whilst female sex was protective.
- Community attitudes toward the effectiveness of scabicides given at health care facilities were high, but health care seeking for scabies was low.
- Knowledge of the community about manifestations of scabies and treatment options was high, however, only slightly more than half of the participants mentioned prolonged physical contact (the main mode of scabies transmission) as a facilitator of scabies transmission.

How this study might affect research, practice, or policy

- Scabies mass drug administrations and accompanying behavioural intervention is needed to control scabies outbreak in Ayu Guagusa district.

For peer review only - http://bmjopen.bmj.com/site/about/guidelines.xhtml

INTRODUCTION

The World Health Organization (WHO) aims to prevent, eliminate or control Neglected Tropical Diseases (NTDs) by 2030 (1). Achieving this is contingent on availability of epidemiological data on NTD burden and distribution (2). However, due to long standing neglect we know little about the epidemiology of many NTDs, including scabies, especially in high-burden low-income settings (3).

Scabies is a skin infestation caused by the mite *Sarcoptes scabiei* (4), which establishes after a pregnant female burrow into the epidermis (4, 5). The average number of burrowing mites on an individual human host ranges between 10 and 15 but severe infestations of 'crusted scabies' (which are less common), can involve thousands of mites on an individual host (6). Typical manifestations include generalized itch, rash, and contact history with a presumed scabies patient (7, 8). Burrows are pathognomonic but rarely identified before thorough physical examination by experienced practitioners (8). Presentation can also include pustules, nodules, chronic excoriation, and skin crust (9). Though itch and sleep deprivation are the primary complications of scabies, secondary bacterial infections are associated with cellulitis, fasciitis, septicaemia, acute glomerulonephritis, and rheumatic heart disease (6, 10, 11). Clinical diagnosis is the most frequently used approach in clinical and field settings, though accuracy relies heavily on examiners' knowledge and experience of scabies (12).

Scabies accounts for an estimated 0.21% of disability-adjusted life-years (DALYs) globally (13). Prolonged physical contact is the main mode of scabies transmission, and circumstances that promote crowding predict increased transmission risk (14, 15). Outbreaks are thus common in institutional settings such as prisons, residential care homes for elderly people, hospitals, and refugee camps (15-17). Among individual level characteristics, age (children, adolescents, and the elderly), low socioeconomic status and travel history to areas of higher scabies prevalence have been identified as increasing risk of developing scabies (12, 18). Facility-based studies rank scabies amongst the top three to five diseases in dermatology clinics (19, 20). On the other hand, community-based studies still indicate a pervasive need for scabies care and a significant delay to care seeking (21, 22). Limited geographic and financial access to health care services, lack of knowledge about the disease and treatment, and stigma and discrimination against scabies patients can contribute to untimely care seeking (20, 22, 23).

Ethiopia reported a major scabies outbreak in 2015 (24). Studies conducted between 2015 and 2017 in the northern and southern parts of the country indicated a prevalence ranging between

For peer review only - http://bmjopen.bmj.com/site/about/guidelines.xhtml

11 and 33% (24-27). This marked a significant increase from the 4 to 7% estimates in pre-outbreak facility-based studies (27-29). Paucity of population-based data on the pattern and drivers of the outbreak has hindered the design of effective control interventions. In this study we aimed to determine the epidemiology of scabies in Ayu Guagusa district and characterize the community's knowledge and perceptions of the infestation.

METHODS

Study design and setting

We conducted this cross-sectional study in Ayu Guagusa district, Amhara region, northern Ethiopia in December 2018. As well as determining scabies epidemiology and community knowledge and attitudes, it was designed to function as a baseline for longitudinal research measuring the secondary impact on scabies prevalence of single dose ivermectin-only Mass Drug Administration (MDA) (30). The Ethiopian nation state is administratively divided into eleven regional states and two metropolitan councils, with regions subdivided into zones, districts and then *kebeles* (the lowest administrative unit comprising 3000-5000 people) (31). The state then organizes households in each *kebele* into *Gotes*, small villages holding semi-official positions in the administrative structure. Amhara is the second most populous region with 17 million people, 85% of whom draw livelihoods from rain-dependent agriculture (32, 33). The Ethiopian state health care system is a three-tier system. Primary health care units provide disease prevention and health promotion and comprise a primary hospital, health center, and five satellite health posts. General hospitals provide secondary level care, with specialized and teaching hospitals serving as centers for tertiary specialized care. Health center staff are mid-level health workers, trained to provide care for patients who do not require advanced medical attention. Health posts are the closest state health facilities to most communities, one post serves 3000 to 5000 people. Health posts are staffed with two health extension workers, who provide primary health care services and facility patient referrals (31).

Sampling procedure

We selected northern Ethiopia for this study as we aimed to investigate the scabies outbreak in the region. Ayu Guagusa district (online supplemental figure S1) was purposively selected as it was the only district, among 11 other districts in Awi zone of Amhara region, in which MDA to eliminate onchocerciasis was underway. There are 21 *kebeles* in the district. Six were selected for this study by using simple random sampling. The number of kebeles is determined by dividing the sample size to the average size of kebele population (3000 to 5000). Then, one *gote* (small village) was randomly selected from each *kebele*. A sampling frame comprising a list of

For peer review only - http://bmjopen.bmj.com/site/about/guidelines.xhtml

households in the selected *gotes* was prepared. Simple random sampling was employed to select study households from the frame. Eventually, all consenting members of selected households participated in the study.

Data collection

Sociodemographic characteristics, health care seeking for scabies manifestations, contact history with a person showing scabies-suggestive symptoms, taking ivermectin tablets during the last round of MDA against onchocerciasis, and knowledge of and attitude toward scabies were measured at the level of the individual participants. Household wealth, household size, and keeping domestic animals in the homestead were measured at the household level.

Data collection instruments and procedures

An interview questionnaire was used to collect data from adult study participants, and parents or guardians of children aged <15y. After the tool was translated to the local language, *Amharic*, we pretested it with 12 people selected from a *kebele* near to the study district.

Clinical diagnosis was employed to identify scabies cases. Classic manifestations such as itch, skin rash with typical distribution, and contact history with a person exhibiting scabies symptoms were considered (12). Clinical examination by naked eye was carried out in sufficiently illuminated rooms in participants' residences. During examination most parts of the body were exposed except the genitals and breasts. A chaperone was present for examinations of participants <18 y. Four nurses and one health officer, who were clinical staff in the nearby primary hospital, conducted the clinical examinations. These had prior experience identifying scabies patients in-clinics and were given a three-day refresher training on scabies diagnosis by a dermatovenerologist (co-author WE) from Bahir Dar University. The training aimed to standardise characterisation of disease manifestations and minimise inter-observer bias in diagnosing scabies. Additionally, during the first few days of the field work, data collectors sent every participant identified as a scabies patient to the dermatologist. At times when there were disagreements in diagnosis, WE discussed the case with the data collectors.

Sample size

We considered an average household size of rural communities 4 and intraclass correlation coefficient of 0.7 (1) to calculate a design effect of 3.1. Including 15% nonparticipation rate we needed to involve 1210 people to detect 33% scabies prevalence with 5% precision. This sample size is also sufficient to fit multi-level logistic regression models with >10 variables while maintaining an events per variable ratio of >10.

For peer review only - http://bmjopen.bmj.com/site/about/guidelines.xhtml

Data processing and analysis

An electronic template was prepared using *Epidata V.3.01* (EpiData Association, Odense) statistical software to enter data from the paper-based questionnaire. Data was later exported to *Stata 14.0* (StataCorp LLC, Texas) for analysis. Summary measures were calculated for categorical data (frequency and percentage) and continuous data (mean and SD or median and IQR). Study households were categorised into wealth quintiles factoring in ownership of fixed household assets. Principal Component Analysis was used to generate components which best captured variability in wealth between the study households. Twenty-six binary variables of household asset and livestock ownership (including access to infrastructure) were included in the analysis (Supplementary File, p. 3) and no significant correlation was observed among these covariates. Twenty-two principal components were generated with varimax rotation, and the first component, which had the highest *eigen value,* was used to generate the wealth index.

The dataset was hierarchical at two levels (i.e., household and individual household member), and there was a high possibility for observations among members of the same household to be correlated. The intra-class correlation coefficient (ICC) was used to characterise the level of correlation between observations from the same household. The ICC was 0.88 (95% CI 0.79–0.94), indicating a significant cluster effect between members of the same study household, justifying the multi-level modelling approach. We thus fitted multi-level logistic regression models to calculate the independent effects of individual and household level predictors on the odds of developing scabies. The models included individual participant level variables (i.e., age, sex, marital status, level of education, occupation, contact history with a person exhibiting signs of scabies, bathing, and participating in the last round of MDA) and three household level variables (i.e., household size, presence of domestic animals in the homestead, and household wealth) and a random effect for household.

We applied the ***melogit*** stata command to fit the models and calculate adjusted Odds Ratio (aOR), with 95% CI to determine the strength and direction of association between the dependent and the predictor variables. Variable inflation factors (VIFs) were calculated to detect multicollinearity among the predictor variables, but none were dropped from the model as all the VIF values were less than 5.

Patient and public involvement

People affected by podoconiosis (another heavily neglected tropical condition) were involved in the development of the funding proposal supporting this study. However, at that point in time

For peer review only - http://bmjopen.bmj.com/site/about/guidelines.xhtml

(2016), there was no scabies patient association in existence in Ethiopia. Subsequent proposals have included input from people affected by scabies, through proposal development workshops and a newly established Community of Practice.

Ethical considerations

Ethical clearance was obtained from Addis Ababa University Institutional Review Board (IRB) and Brighton and Sussex Medical School (BSMS) Research Governance and Ethics Committee (RGEC). Permission was sought from the Ministry of Health (MoH), Amhara regional health Bureau, and local administrative heads before commencing field work. Informed consent was requested and received from all adult participants, and assent from minors 15–17y. Minors <15y participated in the study once their parents or guardians gave consent. Participants diagnosed with scabies or other diseases were referred to the nearest health center for further investigation and treatment.

RESULTS

Participants

Across six *kebeles*, a total of 1437 people (797 females, 640 males) out of 1833 registered members of the 381 study households participated. Three hundred ninety-six people were excluded after data collectors reported failure to find them after three subsequent visits to their households (online supplemental figure S2). All members of the selected households approached for recruitment consented to participate. This may surprise some readers. However, high consent rates are not uncommon in medical research in rural Ethiopian communities (34, 35). This may be explained by the community's respect for and trust of health workers and the frequent routine contact communities have with HEWs when they are doing outreach (36).

Median age was 19y (10-37) (table 1). Almost half the participants (49.5%) were ≤18y, 30.3% were 19-40y, but, broadly in line with Ethiopia's demographics (34), 20.3% were ≥41y (compared to 16% of the national population). Most did not have a formal education (59.7%) and only 7% of participants had secondary level or above education. The most common occupational group was farming (52.2%), followed by being a student (47.8%). Most participants ≥16y were married (38.8% of total participants). The number of households with a household size of <5 (49.2%) and ≥5 (50.8%) was similar. Most study households (70.6%) always used surface water for any purpose. 26.6% of participants reported bathing less than weekly, 54.9% reported bathing 1-2 times weekly, and 18.5% ≥3 times weekly. 13.9% of interviewees stated their clothes were washed less than once per week, 77.7% 1-2 times weekly, and 8.5% ≥3 times weekly. Median time for a round trip to water source was 20 minutes.

For peer review only - http://bmjopen.bmj.com/site/about/guidelines.xhtml

Scabies cases

Scabies prevalence was 13.4% (95% CI 11.8%-15.5%). The highest age specific prevalence was among people aged 11 to 18 years 17.1% (95%CI 13.1%-21.0%) and the lowest among those between 31 and 40 years 6.7% (95%CI 2.9%-10.6%). The prevalence among people in the extremes of age: 0 to 10 years and people older than 41 years was 13.4% (95%CI 9.9%-16.9%) and 12.4% (95%CI 8.6%-16.2%), respectively. The socioeconomic characteristics and scabies diagnoses of those examined are provided in table 1. One-hundred and ninety-two participants had scabies (101 females, 91 males). Participants with clinical scabies were younger (median16y, IQR 10-30y) than those without signs and symptoms (median 20y, IQR 10-38y). Those with no scabies manifestations were evenly split between households with <5 and ≥5 people (respectively: 707, 49.2%; 730, 50.8%), whereas a markedly higher proportion of scabies cases lived in households with ≥5 people (122, 63.5%) than <5 (70, 36.5%). Stated frequency of clothes washing and personal bathing were similar between the scabies cases and those without manifestations.

Clinical presentations of identified scabies cases (n=192) are summarised in supplementary (online supplemental table S2). Vesicles were the most frequently found clinical signs (67.7%). The trunk and head were the most (62.0%) and least (3.6%) involved body parts. Nearly all individuals diagnosed with scabies (184, 95.8%) reported experiencing itch and the majority (165, 85.9%) stated they had a contact history with a presumed scabies case, most stating they believed they had become infested due to contact with a household member (146, 80.7%). Out of the 192 clinical scabies cases, only 62 (32.3%) reported seeking care by the time the data was collected. Of those that did, the majority (40, 64.5%) reported seeking care from a health facility, while a smaller proportion had carried out self-treatment (14, 22.6%). Three (4.8%) said they had used holy water as a remedy. The median number of days between the onset of symptoms and seeking treatment was 20 (IQR 7-30) (online supplemental table S3). Twenty-seven participants said they sought treatment later than this, giving the following reasons: twenty-one (77.8%) because they assumed the signs and symptoms would disappear on their own, three (11.1%) due to lack of time to seek care, two (7.1%) believed medical expenses would be high, and two (7.1%) because they considered the symptoms mild.

Community knowledge and attitudes about scabies

The knowledge and attitudes of adult study participants are summarised in tables 2 and 3. Two thirds (n=500) reported they had heard about scabies. When asked how they believed scabies was transmitted, most stated beliefs in line with established scientific knowledge: prolonged

For peer review only - http://bmjopen.bmj.com/site/about/guidelines.xhtml

physical contact (57.6%); sharing clothes and bedding with a scabies patient (51.4%); sharing a sleeping space with a scabies patient (42.4%). However, some stated modes of transmission not in line with scientific understanding: via contaminated water (10.0%); via blood contact (4.0%); from the soil (2.8%). Most said they wanted to know more about the disease, with a higher proportion interested among the scabies cases. When asked to choose from a list, most (93%) selected body creams and anti-scabies tablets as scabies treatments, while small minorities chose holy water (4.4%), traditional treatment (home remedies and traditional herbs) (3.4%), or prayer (1.4%). 56.3% of adults without scabies said they thought any community member could acquire scabies (table 2). Nearly all (441, 95.7%) said they would seek care from health care facilities if they developed scabies (table 3). 213 (48.3%) reported they would feel nothing emotionally if they developed scabies, but 116 (26.3%) said they would feel shame, and 113 (25.6%) fear. When asked to choose from a list of potential attitudes towards scabies patients, a similar proportion chose a response stating, 'they felt compassion and wanted to help' and 'they feared they would be infected by them' (respectively, 115, 56.4% and 104, 51.0%). 166 (81.4%) chose a response stating, 'they believed their community would prefer to avoid scabies patients', and 46 (22.5%) that 'people would appear friendly but wish to avoid them' (table 3).

Odds of infestation

Table 4 gives the results of the multi-level logistic regression analysis of determinants of scabies infestation. The odds of scabies infestation was lower among females (aOR=0.5, 95% CI 0.3–0.8), and people from households of five or more had higher odds of infestation (aOR=3.5, 95% CI 1.2 - 10.2). The following were not predictive of scabies diagnosis: occupation; education; marital status; age; frequency of bathing; taking ivermectin tablets during prior MDA; keeping domestic animals in the homestead; household wealth.

For peer review only - http://bmjopen.bmj.com/site/about/guidelines.xhtml

DISCUSSION

Scabies prevalence in Ayu Guagusa district was high. Vesicles, excoriations, and papules, often involving the trunk, were the most frequently observed signs, and nearly all participants complained of generalized itch. Only around a third of clinical scabies cases had sought care, with the median number of days before seeking care for the minority who did being 20 days. More than two-thirds had heard about scabies, and friends and family were the commonest sources of scabies-related information. Most believed scabies was transmitted through prolonged physical contact with a scabies patient and that it could be treated with drugs. In the multi-level logistic regression model being from households of size ≥5 people increased odds of scabies infestation but female sex was associated with lower odds of scabies.

There are few studies on scabies in Africa, including Ethiopia, and population-based studies employing primary data are even rarer (21, 24, 26, 37, 38). Scabies prevalence estimates for different parts of Ethiopia range from 2.5 to 33.5% (24). However, since most of these studies were conducted either in areas of scabies outbreaks or were secondary analyses on data related with scabies outbreak response activities, their prevalence estimates may not be representative of the remaining parts of the country (24, 38, 39). In our study, estimated scabies prevalence was 13.4%. We believe this high community prevalence is likely to be, in part, related to the wider ongoing scabies outbreak across Amhara region, first reported in 2015 (41). The prevalence is comparable to studies in southern Ethiopia (26, 37) but lower than estimates from studies elsewhere in the northern parts of the country (24, 40, 41). This may be a secondary impact of prior single dose ivermectin-only onchocerciasis MDA that had taken place 8-months before the current study.

Only around a third of those diagnosed with scabies had reported seeking care of any kind by the time this data was collected. Similar poor health seeking behaviour have been reported in other studies (19, 23). Not seeking care early because of low perceived severity of manifestations, was also found in a study in a Brazilian urban slum (19). Amongst those who sought care, more than two-thirds reported attending a primary health care facility. Even though we could not rule out the effect of social desirability bias (since the data collectors were health workers), the improving physical and financial access to primary health services could have made health care facilities the preferred source of care (42). However, participants reported to the first author that frequent stock-outs of the commonly used cream (permethrin) in many health centres discouraged their attendance. Self-treatment was the second most frequently mentioned source of care. From our anecdotal field observation, people applied local herbs on the lesions.

For peer review only - http://bmjopen.bmj.com/site/about/guidelines.xhtml

Nearly two thirds of the study population had heard about scabies, primarily from family and friends. The now pervasive awareness about scabies is, we suspect, mainly a result of its occurrence as an outbreak. More than half of the study participants in both groups (i.e., clinical scabies and no manifestations of scabies) correctly identified ‘prolonged physical contact with a scabies patient’ as a mode of scabies transmission. There remains room for educational interventions, as over a third in each group did not identify this mode of transmission. Local health worker teaching on scabies has focussed heavily on personal hygiene. However, reported frequency of bathing was not predictive in our study, and experimental and epidemiological evidence from elsewhere indicates personal body washing is probably unrelated to scabies prevalence (43). A better focus would be to prevent prolonged physical contact with presumed scabies cases (e.g., temporary separation of sleeping arrangements for symptomatic individuals), and to treat all contacts once a scabies case is identified. Most participants believed there is treatment for scabies and a comparable number wanted to know more about scabies in general. This presents an opportunity for effective behavioural change communications. Even though we did not use a validated tool to measure attitude, a subtle stigma was associated with scabies patients. This finding was consistent with Trettin *et al*., where scabies patients reported shame and fear of stigma as scabies was perceived to be associated with poor hygiene and poverty (44, 45). Arguably, the present focus on personal hygiene in teachings surrounding scabies may risk contributing to such social stigma against scabies patients.

High scabies prevalence is reported among children in high scabies burden settings, and in a global systematic review by Romani *et al.* (46) prevalence was lower in adults than in children in all relevant studies. In our study, though the association between age and scabies was not statistically significant, the majority of the scabies cases were still among people aged <18y. Household size was a strong predictor; odds of scabies infestation among people living in households with ≥5 members (a household size larger than the national average) (47) was high. Similarly, Hegazy *et al.* (48) reported that people from households of high crowding index had higher odds of scabies infestation in rural Egypt. Since prolonged physical contact is required for effective scabies transmission, high host density both in households and institutional settings facilitates transmission (16).

Recommendations

Mass Drug Administration for scabies need to be commenced in Ayu Guagusa district, as prevalence is higher than 10% (the cut off set for the initiation of preventive chemotherapy by the WHO informal consultation on scabies) (49), which indicates standard care alone is not

For peer review only - http://bmjopen.bmj.com/site/about/guidelines.xhtml

adequate to control the disease. Ivermectin MDAs for scabies were first trialled in Oceania in the late 1990s and have proven to be effective at reducing community prevalence (50). Ideally two doses of oral ivermectin (200 µg/kg) should be given 7-14 days apart, with alternative scabicides provided to whom ivermectin is presently not recommended (children weighing <15 kg or <90 cm height; pregnant women; women breastfeeding in the first week). MDA rounds should have a minimum of 80% population coverage and conducted annually for 3-5 years, with a stopping threshold of 2% community prevalence (49). Accompanying such MDAs with educational interventions may also help to: (1) increase awareness about modes of scabies transmission; (2) address the subtle stigma attached to scabies; and (3) support communities to modify environmental circumstances that facilitate transmission.

Limitations

Our epidemiological findings should be considered with the understanding that a clinical diagnostic approach cannot detect early cases in the asymptomatic incubation period. Purposive selection of the study district where there had been a prior single dose ivermectin-only MDA against onchocerciasis could have treated scabies cases, undermining the true prevalence in the study district. Though in our model, household size predicted increased odds of scabies, we could not objectively attribute this to host density, as we neither measured the size of housing units nor assessed sleeping arrangements.

AUTHORS CONTRIBUTIONS

This study was carried out as part of the work of the NIHR Global Health Research Unit on Neglected Tropical Diseases Phase 1 at Brighton and Sussex Medical School. For clarity we detail contributions using the CRediT Contributor Roles Taxonomy (https://casrai.org/credit/) and provide employment and disciplinary descriptions. Conceptualization: JM, AF, JAC, GD. Analysis: RY, JM. Investigation: RY, AT, WE. Methodology: RY, JM, AF, JAC, SB, CIJ, GD. Supervision: JM, AF, JAC, GD. Writing – original draft: RY, JM. Visualization: JM, AT. Writing – review & editing: RY, JM, AF, JAC, AT, SB, CIJ, WE, GD.

APPENDICES

Supplementary Files accompanying this paper include: study location map; study diagram; author reflexivity statement; tables detailing fixed asset variables used to categorise households into wealth quintiles, scabies manifestations among clinically diagnosed cases, care seeking characteristics of participants who sought care for scabies suggestive symptoms, and distribution of participants by age and sex.

For peer review only - http://bmjopen.bmj.com/site/about/guidelines.xhtml

383 ## ACKNOWLEDGMENTS

384 We thank the field team, health care workers from Gimjabet hospital, for conducting data
385 collection and Dr. Vasso Anagnostopoulou for supporting data management activities. Awi Zone
386 and Ayu Guagusa District Health office administration provided invaluable background
387 information about the institutions in the health system and for facilitating the field work. We are
388 also grateful to the participants who welcomed our field team into their homes and gave their
389 time for the interview.

390 ## FINANCIAL SUPPORT

391 This research was financially supported by the National Institute for Health and Care Research
392 (Grant number 16/136/29). The views expressed are those of the authors and not necessarily
393 those of the NHS, the NIHR or the Department of Health and Social Care.

394 ## CONFLICT OF INTEREST

395 None.

For peer review only

REFERENCES [51/100]

1. World Health Organization. Ending the neglected to attain the sustainable development goals. A road map for neglected tropical diseases 2021–2030. Geneva. 2020. https://www.who.int/publications/i/item/9789240010352 (accessed 01 May 2022).
2. Engelman D, Martin DL, Hay RJ, et al. Opportunities to investigate the effects of ivermectin mass drug administration on scabies. *Parasit Vectors* 2013;6:106.
3. Nagpal S, Sinclair D, Garner P. Has the NTD Community Neglected Evidence-Based Policy? *PLoS Negl Trop Dis* 2013;7(7):1-3.
4. Fuller LC. Epidemiology of scabies. *Curr Opin Infect Dis* 2013;26:123-126.
5. Hay RJ, Steer AC, Engelman D, Walton S. Scabies in the developing world—its prevalence, complications, and management. *Clin Microbiol Infect* 2012;18(4):313–323.
6. Chosidow O. Scabies. *N Engl J Med* 2006;354(16):1718-1727.
7. Mellanby K. Scabies in 1976. *R Soc Health J* 1977;97(1):32-36.
8. Hardy M, Engelman D, Steer A. Scabies: A clinical update. *Aust Fam Physician* 2017;46(5):264-268.
9. Walton SF, Currie BJ. Problems in Diagnosing Scabies, a Global Disease in Human and Animal Populations. *Clin Microbiol Rev* 2007;20(2):268-279.
10. Zaidi AKM, Thaver D, Ali SA, et al. Pathogens Associated With Sepsis in Newborns and Young Infants in Developing Countries. *Pediatr Infect Dis J* 2009;28:10-18.
11. Gao Z, Zhao H, Xia Y,et al.. Clinical Characteristics and Etiologic Analysis of Scabies-Associated Glomerulonephritis. *Intern Med* 2015;5(4):1-5.
12. Engelman D, Fuller LC, Steer AC. Consensus criteria for the diagnosis of scabies: A Delphi study of international expert. *PLoS Negl Trop Dis* 2018;12(5):1-9.
13. Karimkhani C, Colombara DV, Drucker AM, et al. The global burden of scabies: a cross-sectional analysis from the Global Burden of Disease Study 2015. *Lancet Infect Dis* 2017;17:1247-1254.
14. Mellanby K. The development of symptoms, parasitic infection and immunity in human scabies. *Parasitology* 1943;35(4):197-206.
15. Cassell JA, Middleton J, Nalabanda A, et al. Scabies outbreaks in ten care homes for elderly people: a prospective study of clinical features, epidemiology, and treatment outcomes. *Lancet Infect Dis* 2018;18:894-902.
16. Middleton J, Cassell JA, Walker S. Scabies management in institutions. In: Chosidow O, Fischer K, eds. *Scabies*. Berlin: Springer, In Press.
17. Richardson NA, Cassell JA, Head MG, et al. Scabies outbreak management in refugee/migrant camps across Europe 2014-17: a retrospective qualitative interview study of healthcare staff experiences and perspectives. *MedRxiv* 2021 [Preprint]. May 01, 2021 [cited May 10, 2022] https://doi.org/10.1101/2021.04.28.21256211
18. Feldmeier H, Jackson A, Ariza L, et al. The epidemiology of scabies in an impoverished community in rural Brazil: presence and severity of disease are associated with poor living conditions and illiteracy. *J Am Acad Dermatol* 2009;60(3):436-443.
19. Heukelbach Jr, Haeff Ev, Rump B, et al. Parasitic skin diseases: health care-seeking in a slum in north-east Brazil. *Trop Med Int Health* 2003;8(4):368-373.
20. Boateng LA. Healthcare-seeking behaviour in reporting of scabies and skin infections in Ghana: A review of reported cases. *Trans R Soc Trop Med Hyg* 2020(0):1-9.
21. Figueroa JI, Fuller LC, Abraha A,et al. Dermatology in southwestern Ethiopia: rationale for a community approach. *Int J Dermatol* 1998;37(10):752-758.
22. Vincente SL, Kearns T, Connors C,et al. Community Management of Endemic Scabies in Remote Aboriginal Communities of Northern Australia: Low Treatment Uptake and High Ongoing Acquisition. *PLoS Negl Trop Dis* 2009;3(5):1-8.
23. Lopes MJ, Silva ETd, Ca J, et al. Perceptions, attitudes and practices towards scabies in communities on the Bijagós Islands, Guinea-Bissau. *Trans R Soc Trop Med Hyg* 2020;114(1):49-56.
24. Enbiale W, Ayalew A. Investigation of a Scabies Outbreak in Drought-Affected Areas in Ethiopia. *Trop Med Infect Dis* 2018;3(114):1-9.

For peer review only - http://bmjopen.bmj.com/site/about/guidelines.xhtml

25. Yassin ZJ, Dadi AF, Nega HY,et al. Scabies Outbreak Investigation among “Yekolo Temaris” in Gondar Town, North Western Ethiopia, November 2015. *Electron J Biol* 2017;13(3):203-209.
26. Sara J, Haji Y, Gebretsadik A. Scabies Outbreak Investigation and Risk Factors in East Badewacho District, Southern Ethiopia: Unmatched Case Control Study. *Dermatol Res Pract 2018*;2018:1-10.
27. Accorsia S, Barnabasb GA, Faresea P, et al. Skin disorders and disease profile of poverty: analysis of medical records in Tigray, Northern Ethiopia, 2005—2007. *Trans R Soc Trop Med Hyg* 2009;103.
28. Figueroa JI, Hawranek T, Abraha A, et al. Prevalence of skin diseases in school children in rural and urban communities in the Illubabor province, south-western Ethiopia: a preliminary survey. *J Eur Acad Dermatol Venereol* 1997;9:142-148.
29. Walker SL, Lebas E, Sario VD, et al. The prevalence and association with healthrelated quality of life of tungiasis and scabies in schoolchildren in southern Ethiopia. *PLoS Negl Trop Dis* 2017;11(8):1-11.
30. Yirgu R, Middleton J, Fekadu A, et al. The secondary impact of ivermectin MDA for the control of onchocerciasis on the prevalence of scabies in the Amhara region of northern Ethiopia. In: *International Alliance for the Control of Scabies Annual Scientific Meeting 2021*, November 2021, online.
31. Federal Democratic Republic of Ethiopia Ministry of Health. *Health Sector Development Programme IV*. 2000. https://e-library.moh.gov.et/library/index.php/moh-resource-library/?wpv_post_search=hsdp&wpv_aux_current_post_id=1677&wpv_aux_parent_post_id=1677&wpv_view_count=1678 (accessed 04 May 2022).
32. Federal Democratic Repyblic of Ethiopia Population Census Commission. *Summary and Statistical Report of the 2007 Population and Housing Census*. Addis Ababa: United Nations Population Fund, 2008. https://www.statsethiopia.gov.et/census-2007-2/ (accessed 07 May 2022).
33. Central Statistics Agency (CSA). Ethiopia Socioeconomic Survey. 2019. https://www.statsethiopia.gov.et/ (accessed 04 May 2022).
34. Regasa MT, Hinkosa L, Besho M, et al. Predictors of preterm birth in Western Ethiopia: A case control study. *PLoS One* 2021;16(4):e0247927.
35. Legesse A, Adisalem D. Knowledge, Attitude and Practice toward legalization of abortion among female students of Sabiyan secondary and preparatory school, Dire Dawa, Eastern Ethiopia, 2018. A descriptive cross-sectional study. *Asian J Medl Res* 2020;9(2).
36. Assefa Y, Gelaw YA, Hill PS,et al.. Community health extension program of Ethiopia, 2003–2018: successes and challenges toward universal coverage for primary healthcare services. *Globalization and Health* 2019;15(1):24.
37. Wochebo W, Haji Y, Asnake S. Scabies outbreak investigation and risk factors in Kechabira district, Southern Ethiopia: unmatched case control study. *BMC Res Notes* 2019;12(305):1-6.
38. Azene AG, Aragaw AM, Wassie GT. Prevalence and associated factors of scabies in Ethiopia: systematic review and Meta-analysis. *BMC Infect Dis* 2020;20(1):1-10.
39. Enbiale W, Baynie TB, Ayalew A, et al. "Stopping the itch": mass drug administration for scabies outbreak control covered for over nine million people in Ethiopia. *J Infect Dev Countr* 2020;14(6.1):28S-35S.
40. Enbiale W, Ayalew A, Gebrehiwot T, , et al. Does mass drug administration for community-based scabies control works? The experience in Ethiopia. *J Infect Dev Countr* 2020;14(6.1):78s-85s.
41. Accorsi S, Barnabas GA, Farese P, et al. Skin disorders and disease profile of poverty: analysis of medical records in Tigray, northern Ethiopia, 2005-2007. *Trans R Soc Trop Med Hyg* 2009;103(5):469-475.
42. Demissie B, Negeri KG. Effect of Community-Based Health Insurance on Utilization of Outpatient Health Care Services in Southern Ethiopia: A Comparative Cross-Sectional Study. *Risk Management and Healthcare Policy* 2020(13):141-143.
43. Middleton J, Cassell JA, Jones CI et al. Scabies control: the forgotten role of personal hygiene - Authors’ reply. *Lancet Infect Dis* 2018;18(10):1068-1069.

For peer review only - http://bmjopen.bmj.com/site/about/guidelines.xhtml

44. Trettin B, Lassen JA, Andersen F, et al. The journey of having scabies–A qualitative study. *J Nur Edu Pract*. 2019;9(2):1-9.

45. Jin-gang A, Sheng-xiang X, Sheng-bin X, et al. Quality of life of patients with scabies. *J Euro A of Dermatol Venereol* 2010;24:1187-1191.46. Romani L, Steer AC, Whitfeld MJ, et al. Prevalence of scabies and impetigo worldwide: a systematic review. *Lancet Infect Dis* 2015;15:960-967.

47. Central Statistical Agency (CSA) Ethiopia and ICF. *Ethiopia Demographic and Health Survey 2016*. Addis Ababa Ethiopia and Rockville, Maryland, USA; 2016. https://evaw-global-database.unwomen.org/en/countries/africa/ethiopia/2016/demographic-and-health-survey-2016 (accessed 06 May 2022).

48. Hegazy AA, Darwish NM, Abdel-Hamid IA,et al. Epidemiology and control of scabies in an Egyptian village. *Int J Dermatol* 1999;38:291-295.

49. World Health Organization. *WHO Informal Consultation on a Framework for Scabies Control*. Manila; 2019. https://www.who.int/publications/i/item/9789240008069 (accessed 26 May 2022).

50. Middleton J. Can ivermectin mass drug administrations to control scabies also reduce skin and soft tissue infections? Hospitalizations and primary care presentations lower after a large-scale trial in Fiji. *Lancet Regional Health – Western Pacific* 2022;22:100454.

For peer review only

For peer review only - http://bmjopen.bmj.com/site/about/guidelines.xhtml

TABLES

Table 1 Socioeconomic characteristics of the study sample (n=1437)

Variables	Categories	Overall (n=1437)	Scabies diagnosis	
			No scabies manifestations (n=1245)	Clinical scabies (n=192)
**Sex**	Male	640 (44.5)	539 (43.3)	101 (52.6)
	Female	797 (55.5)	706 (56.7)	91 (47.4)
**Age in years**	≤10	365 (25.4)	316 (25.4)	49 (25.5)
	11–18	346 (24.1)	287 (23.1)	59 (30.7)
	19–40	435 (30.3)	387 (31.1)	48 (25.0)
	≥41[π]	291 (20.3)	255 (20.5)	36 (18.8)
	Median age (IQR)	19 (10-37)	20 (10-38)	16 (10-30)
**Level of education* (n=1422)**	No formal education	849 (59.7)	745 (60.6)	104 (54.2)
	Primary education	474 (33.3)	401 (32.6)	73 (38.0)
	Secondary and above	99 (7.0)	84 (6.8)	15 (7.8)
**Occupation (n=1412)**	Farmer	737 (52.2)	650 (53.3)	87 (45.3)
	Student	675 (47.8)	570 (46.7)	105 (54.7)
**Marital status[#]**	Not married	228 (15.9)	204 (16.4)	24 (12.5)
	Married	558 (38.8)	487 (39.1)	71 (37.0)
	<16y	651 (45.3)	554 (44.5)	97 (50.5)
**Household size**	<5 people	707 (49.2)	637 (51.2)	70 (36.5)
	≥5 people	730 (50.8)	608 (48.8)	122 (63.5)
**Contact history with symptomatic person**	No	947 (65.9)	939 (75.4)	8 (4.2)
	Yes	490 (34.1)	306 (24.6)	184 (95.8)
**Took pill during the last MDA**	No	338 (23.5)	274 (22.0)	64 (33.3)
	Yes	1099 (76.5)	971 (78.0)	128 (66.7)
**Domestic animals kept in the homestead**	No	202 (14.1)	171 (13.7)	31 (16.2)
	Yes	1235 (85.9)	1074 (86.3)	161 (83.9)
**Household wealth**	Lowest	291 (20.3)	266 (21.4)	25 (13.0)
	Second	277 (19.3)	240 (19.3)	37 (19.3)
	Middle	293 (20.4)	255 (20.5)	38 (19.8)
	Fourth	293 (20.4)	261 (20.9)	32 (16.7)
	Highest	283 (19.7)	223 (17.9)	60 (31.3)
**Main source of water for any purpose[‡]**	Public tap	181 (12.6)	139 (11.3)	42 (21.9)
	Dug well	405 (28.2)	355 (28.5)	50 (26.0)
	Spring water	850 (59.2)	746 (59.9)	104 (54.2)
	Surface water	1014 (70.6)	869 (69.8)	145 (75.5)
	No Response	0 (0)	0 (0)	0 (0)
**Frequency of bathing per week**	<1	382 (26.6)	326 (26.2)	56 (29.2)
	1-2	789 (54.9)	688 (55.3)	101 (52.6)
	≥3	266 (18.5)	231 (18.6)	35 (18.2)
**Frequency of washing clothes per week**	<1	199 (13.9)	174 (13.9)	25 (13.0)
	1-2	1116 (77.7)	967 (77.7)	149 (77.6)
	≥3	122 (8.5)	104 (8.4)	18 (9.4)

[π] People >40 y comprises just 16% of the Ethiopian population; *This category comprised people with no formal education and children younger than school age; [#] Marital status of participants was only asked of those ≥16y. [‡]Total scores exceed 100% as individual participants could select multiple response categories.

For peer review only

For peer review only - http://bmjopen.bmj.com/site/about/guidelines.xhtml

Table 2 Knowledge of adult study participants about scabies (n=500)

Variable	Categories	Overall n (%) *	Scabies diagnosis among adults	
			No scabies Manifestations (n=430)	Clinical scabies (n=70)
From what sources first heard about scabies	Family and friends	320 (64.0)	269 (62.6)	51 (72.9)
	Health care providers	71 (14.2)	65 (15.1)	6 (8.6)
	Health extension workers	48 (9.6)	9 (2.9)	39 (55.7)
	Mass media	44 (8.8)	33 (7.7)	11 (15.7)
	Religious leaders	18 (3.6)	18 (4.2)	0
	Not collected	44 (8.8)	42 (9.8)	2 (2.9)
What are the manifestations of scabies?	Itch	449 (89.8)	382 (88.8)	67 (95.7)
	Rash	242 (48.4)	187 (43.5)	55 (78.6)
	Skin crust	24 (4.8)	17 (3.9)	7 (10.0)
How is scabies transmitted?	Prolonged physical contact with a scabies patient	288 (57.6)	245 (56.9)	43 (61.4)
	Sharing clothing and bedding with a scabies patient	257 (51.4)	216 (50.2)	41 (58.6)
	Sharing sleeping space with scabies patient	212 (42.4)	175 (40.7)	37 (52.9)
	From contaminated water	50 (10.0)	49 (11.4)	1 (1.4)
	Blood contact with a scabies patient	20 (4.0)	18 (4.2)	2 (2.9)
	From the soil	14 (2.8)	12 (2.8)	2 (2.9)
	No response	58 (11.6)	49 (11.4)	9 (12.9)
Is scabies treatable?	Yes	450 (90.0)	387 (90.0)	63 (90.0)
	No	45 (9.0)	39 (9.1)	6 (8.6)
	No response	5 (1.0)	4 (1.0)	1 (1.4)
What is the treatment for scabies?	Body creams and tablets	469 (93.8)	404 (93.9)	65 (92.8)
	Holy water	22 (4.4)	21 (4.9)	1 (1.4)
	Traditional treatment	17 (3.4)	16 (3.7)	1 (1.4)
	Prayer	7 (1.4)	5 (1.2)	2 (2.8)
	No Response	0 (0)	0 (0)	0 (0)
Who can catch scabies?	Any one	282 (56.3)	235 (54.5)	47 (67.1)
	Poor people	53 (10.6)	42 (9.7)	11 (15.7)
	Rich people	14 (2.8)	11 (2.6)	3 (4.3)
	Homeless people	24 (4.8)	20 (4.6)	4 (5.7)
	Urban people	14 (2.8)	12 (2.8)	2 (2.9)
	Rural people	49 (11.1)	38 (10.1)	11 (16.9)
	Commercial sex workers	22 (4.4)	19 (4.4)	3 (4.3)
	Prisoners	22 (4.4)	18 (4.2)	4 (5.7)
	No response	44 (8.8)	39 (9.1)	5 (7.1)
Do you wish to know more about scabies?	Yes	435 (87.0)	371 (86.3)	64 (91.4)
	No	65 (13.0)	59 (13.7)	6 (8.6)

*Total scores may exceed 100% for some questions when individual participants could select more than one response categories.

For peer review only - http://bmjopen.bmj.com/site/about/guidelines.xhtml

Table 3 Attitude of adult study participants without scabies symptoms towards scabies (n=441)

Question	Categories	n (%) *
**Do you think you can acquire scabies?**	Yes	265 (61.0)
	No	169 (38.3)
	No response	7 (1.6)
**What would you feel if you developed scabies?**	Nothing	213 (48.3)
	Shame	116 (26.3)
	Fear	113 (25.6)
	Disappointment	68 (15.4)
	Surprise	16 (3.6)
	No response	13 (2.9)
**What would you do if you developed scabies?**	Seek care from health care facilities	422 (95.7)
	Use traditional medicine	2 (0.5)
	Do nothing because it improves on its own	2 (0.5)
	No response	16 (3.6)
**What is your feeling towards scabies patients? (n=204) ****	I feel compassion and wish to help	115 (56.4)
	I fear they may infect me	104 (51.0)
	I do not feel anything	66 (32.4)
	I feel compassion but prefer to stay away	40 (19.4)
	No response	6 (2.9)
**How does the community treat scabies patients? (n=204) ****	Most people prefer to avoid scabies patients	166 (81.4)
	People help and support them	78 (38.2)
	People appear friendly but they wish they avoided them	46 (22.5)
	No response	25 (22.3)

*All items in this table were multiselect and participants could give more than one response for each variable, and this might cause the cumulative score of some variables to exceed the total number of participants. **Calculated out of 204 participants who have knowledge of a scabies patient.

For peer review only

For peer review only - http://bmjopen.bmj.com/site/about/guidelines.xhtml

Table 4 Multi-level logistic regression analysis results of determinants of scabies infestation (Number of households N=381; number of participants N=1403; number of cases 192).

Variable	Category	aOR	95% CI
**Sex**	Male (ref)		
	Female	0.5	0.3 - 0.8
**Age (years)**	≤ 17 (ref)		
	18 - 40	0.5	0.0 - 4.4
	≥ 41	0.4	0.0 - 4.2
**Occupation**	Farmer (ref)		
	Student	0.9	0.3 - 3.2
**Education**	No formal education (ref)		
	Primary education	1.1	0.5 - 2.3
	Secondary and higher	2.8	0.8 - 9.9
**Marital status**	Not married (ref)		
	Married	0.8	0.1 - 7.0
	<16	1.3	0.5 - 3.8
**Frequency of bathing per week**	<1 (ref)		
	1-2 times	0.6	0.3 - 1.4
	≥3 times	1.0	0.3 - 2.9
**Took ivermectin during last MDA**	No (ref)		
	Yes	0.7	0.2 - 3.0
**Household size**	<5 people (ref)		
	≥5 people	3.5	1.2 - 10.2
**Domestic animals kept in homestead**	No (ref)		
	Yes	0.7	0.2 - 3.0
**Household wealth**	Lowest (ref)		
	Second	0.6	0.1 - 2.7
	Middle	0.8	0.2 - 4.1
	Fourth	0.8	0.2 - 4.2
	Highest	3.5	0.8 - 15.1
**Intracluster correlation coefficient (ICC, 95%CI)**		0.89	0.79 - 0.94

aOR: Adjusted odds ratio

For peer review only

For peer review only - http://bmjopen.bmj.com/site/about/guidelines.xhtml

Supplementary Materials

Yirgu, Middleton, Fekadu *et al.* Scabies in the Amhara region of northern Ethiopia: a cross-sectional study of prevalence, determinants, clinical presentation, and community knowledge. Submitted to *BMJ Global Health* April 2023.

Table of Contents

Figure S1. Study area and study locations

Table S1. Fixed asset variables used to categorise households into wealth quintiles, with justifications

Figure S2. Study diagram, number of participants, and place of residence (*kebeles*)

Table S2. Scabies manifestations among clinically diagnosed cases

Table S3. Care-seeking characteristics of participants who sought care for scabies suggestive symptoms

Table S4. Distribution of participants by age and sex

For peer review only

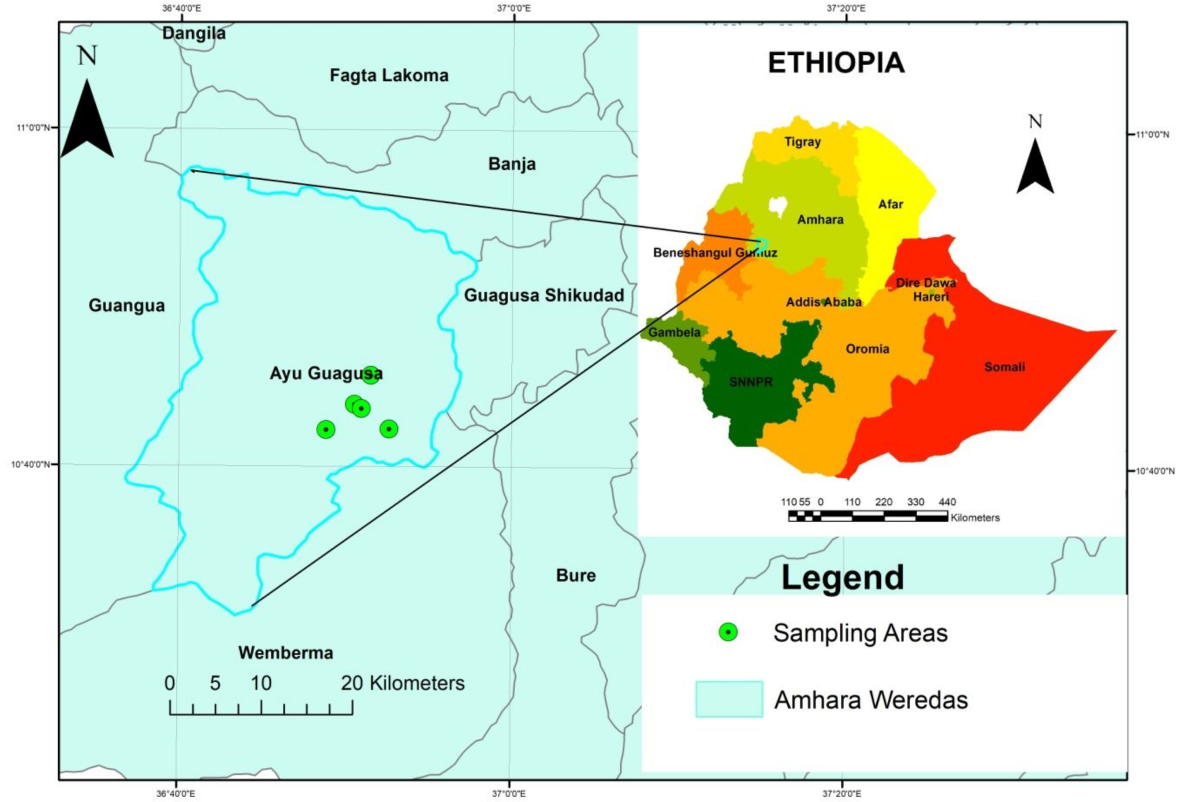

Figure S1. Study area and locations**:** Amhara Regional State; Ayu Guagusa district in Awi zone; sampled kebeles. Produced using ArcGIS version 10.8 (ESRI, Redlands, USA).

For peer review only - http://bmjopen.bmj.com/site/about/guidelines.xhtml

Table S1. Fixed asset variables used to categorise households into wealth quintiles.

**Household asset and infrastructure related variables**
Does your house have electricity?
Does your household own a clock?
Does your household own a radio?
Does your household own a television?
Does any member of your household own a mobile phone?
Does your household own a refrigerator?
Does your household own a table?
Does your household own chair?
Does your household own bed with cotton or sponge mattress?
Does your household own an electric hot-plate?
Does your household own a kerosene lamp?
Does any member of your household own a bicycle?
Does any member of your household own a motorcycle?
Does your household own animal drawn cart?
Does your household own a car?
Does your household own farm animals?
Does your household own piped water into the dwelling?
Does your household own piped water into the yard?
Does your household have access to public tap?
Does your household own a dug well?
Does your household use spring water?
Does your household use rainwater?
Does your household use water distributed using tanker trucks?
Is the floor of your house made of sand or earth or sand?
Is the floor of your house made of ceramic tiles?

For peer review only

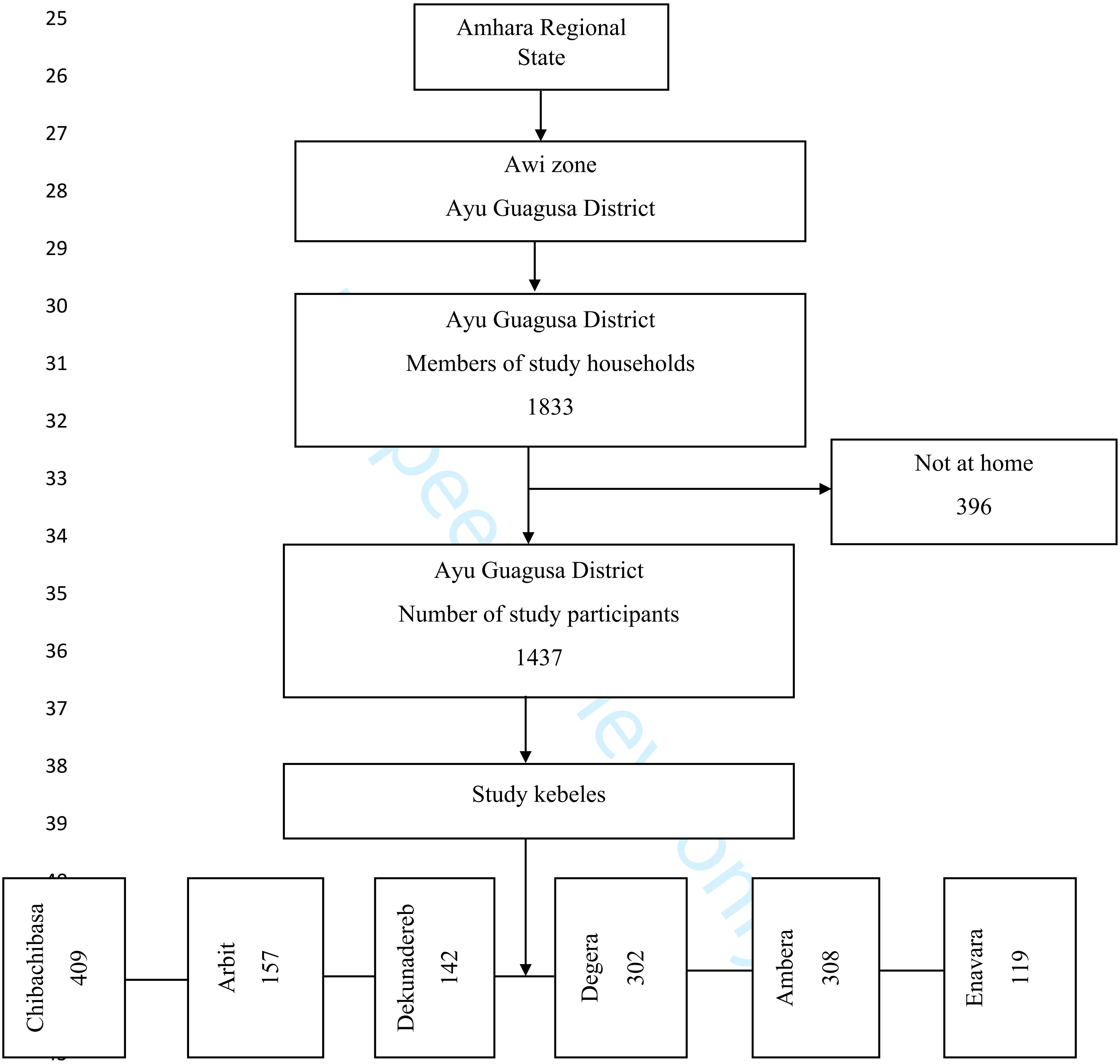

Figure S2. Study diagram, number of participants, and place of residence (*kebeles*)

For peer review only - http://bmjopen.bmj.com/site/about/guidelines.xhtml

Table S2 Scabies manifestations among clinically diagnosed cases (n=192)

Variable	Categories	n (%) *
**Skin lesions**	Vesicles	130 (67.7)
	Scratch marks	117 (60.9)
	Papules	99 (51.6)
	Skin crust	78 (40.6)
	Pustule	52 (27.1)
	Burrows	8 (4.2)
**Skin rash distribution**	Trunk	119 (62.0)
	Elbow	99 (51.6)
	Finger webs	98 (51.0)
	Wrist	81 (42.2)
	Knee	38 (19.8)
	Neck	25 (13.0)
	Ankle	12 (6.3)
	Head	7 (3.6)
**History features**	Itch	184 (95.8)
	Contact history with a presumed scabies patient	184 (95.8)
**Relationship of participant with participant presumed source of infestation (n=184)** [#]	Household members	146 (79.3)
	Neighbour	20 (10.9)
	Friend	15 (8.2)

*Cumulative scores for some variables were more than the number of participants as the items were multi select.

[#] Calculated out of the number of participants who reported contact history with a person exhibiting signs of scabies.

For peer review only - http://bmjopen.bmj.com/site/about/guidelines.xhtml

For peer review only

Table S3 Care seeking characteristics of participants who sought care for scabies suggestive symptoms (n=62)

Variable	Categories	n (%)
**Source of care**	Health facility	40 (64.5)
	Self-treatment	14 (22.6)
	Holy water	3 (4.8)
	Prayer	1 (1.6)
	Missing values	4 (6.5)
**Time to care seeking**	Median number of days (IQR)	20 (7–30)

For peer review only

For peer review only - http://bmjopen.bmj.com/site/about/guidelines.xhtml

Table S4 Distribution of participants by age and sex (n=1437)

Sex	Age categories	Total (%) (n=1437)	No scabies (%) (n=1245)	Clinical scabies (%) (n=192)
**Male**	0–10	173 (27.0)	148 (27.5)	25 (24.8)
	11–18	170 (26.6)	134 (24.9)	36 (35.6)
	19–40	165 (25.8)	144 (26.7)	21 (20.8)
	>41	132 (20.6)	113 (20.9)	19 (18.8)
**Female**	0–10	192 (24.1)	168 (23.8)	24 (26.4)
	11–18	176 (22.1)	153 (21.7)	23 (25.3)
	19–40	270 (33.9)	243 (34.4)	27 (29.7)
	>41	159 (19.9)	142 (20.1)	17 (18.7)

For peer review only

For peer review only - http://bmjopen.bmj.com/site/about/guidelines.xhtml

For peer review only

For peer review only - http://bmjopen.bmj.com/site/about/guidelines.xhtml

BMJ Open

Scabies in the Amhara region of northern Ethiopia: a cross-sectional study of prevalence, determinants, clinical presentation, and community knowledge

Journal:	*BMJ Open*
Manuscript ID	bmjopen-2023-075038.R1
Article Type:	Original research
Date Submitted by the Author:	18-Aug-2023
Complete List of Authors:	Yirgu, Robel; Addis Ababa University College of Health Sciences, Public Health; Brighton and Sussex Medical School,<br>Middleton, Jo; Brighton and Sussex Medical School,<br>Fekadu, Abebaw; Addis Ababa University, Centre for Innovative Drug Development and Therapeutic Trials for Africa (CDT-Africa); Brighton and Sussex Medical School, Department of Global Health & Infection<br>Cassell, Jackie; Brighton and Sussex Medical School<br>Tesfaye, Abraham; Therapeutic Trials for Africa (CDT-Africa) CDT-Africa<br>Jones, Christopher; Brighton and Sussex Medical School<br>Bremner, Stephen; University of Sussex, Department of Primary Care and Public Health<br>Enbiale, Wendemagegn; Bahir Dar University College of Medical and Health Sciences<br>Davey, Gail; University of Sussex, Wellcome Trust Brighton and Sussex Centre for Global Health Research, Brighton and Sussex Medical School, Brighton
**Primary Subject Heading**:	Epidemiology
Secondary Subject Heading:	Infectious diseases, Public health
Keywords:	Epidemiology < TROPICAL MEDICINE, Infectious diseases & infestations < DERMATOLOGY, Public health < INFECTIOUS DISEASES, Neglected Diseases, Dermatological epidemiology < DERMATOLOGY

SCHOLARONE™
Manuscripts

I, the Submitting Author has the right to grant and does grant on behalf of all authors of the Work (as defined in the below author licence), an exclusive licence and/or a non-exclusive licence for contributions from authors who are: i) UK Crown employees; ii) where BMJ has agreed a CC-BY licence shall apply, and/or iii) in accordance with the terms applicable for US Federal Government officers or employees acting as part of their official duties; on a worldwide, perpetual, irrevocable, royalty-free basis to BMJ Publishing Group Ltd ("BMJ") its licensees and where the relevant Journal is co-owned by BMJ to the co-owners of the Journal, to publish the Work in this journal and any other BMJ products and to exploit all rights, as set out in our licence.

The Submitting Author accepts and understands that any supply made under these terms is made by BMJ to the Submitting Author unless you are acting as an employee on behalf of your employer or a postgraduate student of an affiliated institution which is paying any applicable article publishing charge ("APC") for Open Access articles. Where the Submitting Author wishes to make the Work available on an Open Access basis (and intends to pay the relevant APC), the terms of reuse of such Open Access shall be governed by a Creative Commons licence – details of these licences and which Creative Commons licence will apply to this Work are set out in our licence referred to above.

Other than as permitted in any relevant BMJ Author's Self Archiving Policies, I confirm this Work has not been accepted for publication elsewhere, is not being considered for publication elsewhere and does not duplicate material already published. I confirm all authors consent to publication of this Work and authorise the granting of this licence.

For peer review only

Scabies in the Amhara region of northern Ethiopia: a cross-sectional study of prevalence, determinants, clinical presentation, and community knowledge

Robel Yirgu[1,2], Jo Middleton[1,3*], Abebaw Fekadu[1,4], Jackie A Cassell[1,3], Abraham Tesfaye[4], Christopher Iain Jones[3], Stephen Bremner[1,3], Wendemagegn Enbiale[5], Gail Davey[1,2]

[1]NIHR Global Health Research Unit on Neglected Tropical Diseases, Brighton and Sussex Medical School, Falmer, UK. [2]School of Public Health, Addis Ababa University, Addis Ababa, Ethiopia. [3]Primary Care and Public Health, Brighton and Sussex Medical School, Falmer, UK. [4]Center for Innovative Drug Development and Therapeutic Trials for Africa, Addis Ababa University, Addis Ababa, Ethiopia. [5]Bahir Dar University, Bahir Dar, Ethiopia.

*Correspondence: Robel Yirgu - yirgurob@gmail.com

Keywords: Scabies, Ethiopia, Neglected Tropical Diseases, *Sarcoptes scabiei*

For peer review only - http://bmjopen.bmj.com/site/about/guidelines.xhtml

Abstract

Background The World Health Organization aims to prevent, eliminate, or control Neglected Tropical Diseases (NTDs), including scabies, by 2030. However, limited epidemiological data presented a challenge to control efforts, especially in high burden countries. There was a major scabies outbreak in northern Ethiopia starting in 2015 and prevalence has since increased across much of the country.

Objective To estimate scabies prevalence, identify its predictors, and assess community perception of, and knowledge about, the infestation.

Design Population-based cross-sectional study.

Study setting Ayu Guagusa district, Amhara region, northern Ethiopia.

Participants 1437 people who were members of 381 randomly selected households participated in the study. Five trained mid-level health workers clinically diagnosed people with scabies.

Outcome measures Clinically diagnosed scabies infestation.

Data analysis Multi-level logistic regression models were fitted to adjust for individual and household-level confounding variables, and identify predictors of scabies infestation.

Results Scabies prevalence was 13.4% (95% CI 11.8 - 15.5). Households of more than five people (adjusted Odds Ratio (aOR)=3.5, 95% CI 1.2 - 10.2) were associated with increased odds of developing scabies, however, females had lower odds (aOR=0.5 95% CI 0.3 - 0.8). Scabietic lesions most frequently involved the trunk (62.0%), and vesicles were the commonest types of lesions (67.7%). Two-thirds of adult study participants had heard about scabies and most obtained scabies related information from informal sources. Only 32% of cases sought care for scabies from any source.

Conclusion Scabies prevalence was high, signifying the need for community-based control interventions. Host density and sex were important predictors of scabies. Despite the favourable attitude toward the effectiveness of scabies treatment, health care seeking was low.

For peer review only - http://bmjopen.bmj.com/site/about/guidelines.xhtml

Strengths and limitations of the study

- The clinical diagnosis that we employed in this study cannot detect cases in the asymptomatic incubation period.
- Physical examination of participants did not involve private body parts and we might have missed some people with scabies.
- Single dose ivermectin-only Mass Drug Administration (MDA) against onchocerciasis had been underway in the study district. This could have treated scabies cases, undermining the true prevalence.
- Though in our model, household size predicted increased odds of scabies, we could not objectively attribute this to host density, as we neither measured the size of housing units nor assessed sleeping arrangements.

For peer review only

For peer review only - http://bmjopen.bmj.com/site/about/guidelines.xhtml

Introduction

The World Health Organization (WHO) aims to prevent, eliminate or control Neglected Tropical Diseases (NTDs) by 2030 (1). Achieving this is contingent on availability of epidemiological data on NTD burden and distribution (2). However, due to long standing neglect we know little about the epidemiology of many NTDs, including scabies, especially in high-burden low-income settings (3).

Scabies is a skin infestation caused by the mite *Sarcoptes scabiei* (4), which is established after a pregnant female burrow into the epidermis (4, 5). The average number of burrowing mites on an individual human host ranges between 10 and 15 but severe infestations of 'crusted scabies' (which are less common), can involve thousands of mites on an individual host (6). Typical manifestations include generalized itch, rash, and contact history with a presumed scabies patient (7, 8). Burrows are pathognomonic but rarely identified before thorough physical examination by experienced practitioners (8). Presentation can also include pustules, nodules, chronic excoriation, and skin crust (9). Though itch and sleep deprivation are the primary complications of scabies, secondary bacterial infections are associated with cellulitis, fasciitis, septicaemia, acute glomerulonephritis, and rheumatic heart disease (6, 10, 11). Clinical diagnosis is the most frequently used approach in clinical and field settings, though accuracy relies heavily on examiners' knowledge and experience of scabies (12).

Scabies accounts for an estimated 0.21% of disability-adjusted life-years (DALYs), globally (13). Prolonged physical contact is the main mode of scabies transmission, and circumstances that promote crowding predict increased transmission risk (14, 15). Outbreaks are thus common in institutional settings such as prisons, residential care homes for elderly people, hospitals, and refugee camps (15, 16). Among individual level characteristics, age (children, adolescents, and the elderly), low socioeconomic status and travel history to areas of higher scabies prevalence have been identified as increasing risk of developing scabies (12, 17). Facility-based studies rank scabies amongst the top three to five diseases in dermatology clinics (18, 19). On the other hand, community-based studies still indicate a pervasive need for scabies care and a significant delay to care seeking (20, 21). Limited geographic and financial access to health care services, lack of knowledge about the disease and treatment, and stigma and discrimination against scabies patients can contribute to untimely care seeking (19, 21, 22).

Ethiopia reported a major scabies outbreak in 2015 (23). Studies conducted between 2015 and 2017 in the northern and southern parts of the country indicated a prevalence ranging between

For peer review only - http://bmjopen.bmj.com/site/about/guidelines.xhtml

11 and 33% (23-26). This marked a significant increase from the 4 to 7% estimates in pre-outbreak facility-based studies (26-28). Paucity of population-based data on the pattern and drivers of the outbreak has hindered the design of effective control interventions. In this study we aimed to determine the epidemiology of scabies in Ayu Guagusa district and characterize the community's knowledge and perceptions of the infestation.

Methods

Study design and setting

We conducted this cross-sectional study in Ayu Guagusa district, Amhara region, northern Ethiopia in December 2018. As well as determining scabies epidemiology and community knowledge and attitudes, it was designed to function as a baseline for longitudinal research measuring the secondary impact on scabies prevalence of single dose ivermectin-only Mass Drug Administration (MDA) (29). The Ethiopian nation state is administratively divided into eleven regional states and two metropolitan councils, with regions subdivided into zones, districts and then *kebeles* (the lowest administrative unit comprising 3000-5000 people) (30). The state then organizes households in each *kebele* into *Gotes*, small villages holding semi-official positions in the administrative structure. Amhara is the second most populous region with 17 million people, 85% of whom draw livelihoods from rain-dependent agriculture (31, 32). The Ethiopian state health care system is a three-tier system. Primary health care units provide disease prevention and health promotion and comprise a primary hospital, health center, and five satellite health posts. General hospitals provide secondary level care, with specialized and teaching hospitals serving as centers for tertiary specialized care. Health center staff are mid-level health workers, trained to provide care for patients who do not require advanced medical attention. Health posts are the closest state health facilities to most communities, one post serves 3000 to 5000 people. Health posts are staffed with two health extension workers, who provide primary health care services and facility patient referrals (30).

Sampling procedure

We selected northern Ethiopia for this study as we aimed to investigate the scabies outbreak in the region. Ayu Guagusa district (online supplemental figure S1) was purposively selected as it was the only district, among 11 other districts in Awi zone of Amhara region, in which MDA to eliminate onchocerciasis was underway. There are 21 *kebeles* in the district. Six were selected for this study by using simple random sampling. The number of kebeles is determined by dividing the sample size to the average size of kebele population (3000 to 5000). Then, one *gote*

For peer review only - http://bmjopen.bmj.com/site/about/guidelines.xhtml

(small village) was randomly selected from each *kebele*. A sampling frame comprising a list of households in the selected *gotes* was prepared. Simple random sampling was employed to select study households from the frame. Eventually, all consenting members of selected households participated in the study.

Data collection

Sociodemographic characteristics, health care seeking for scabies manifestations, contact history with a person showing scabies-suggestive symptoms, taking ivermectin tablets during the last round of MDA against onchocerciasis, and knowledge of and attitude toward scabies were measured at the level of the individual participants. Household wealth, household size, and keeping domestic animals in the homestead were measured at the household level.

Data collection instruments and procedures

An interview questionnaire was used to collect data from adult study participants, and parents or guardians of children aged <15y (online supplemental table S1). After the tool was translated to the local language, *Amharic*, we pretested it with 12 people selected from a *kebele* near to the study district.

Clinical diagnosis was employed to identify scabies cases. Classic manifestations such as itch, skin rash with typical distribution, and contact history with a person exhibiting scabies symptoms were considered (12). Clinical examination with naked eye was carried out in sufficiently illuminated rooms in participants' residences. During examination most parts of the body were exposed except the genitals and breasts. A chaperone was present for examinations of participants <18 y. Four nurses and one health officer, who were clinical staff in the nearby primary hospital, conducted the clinical examinations. These had prior experience identifying scabies patients in-clinics and were given a three-day refresher training on scabies diagnosis by a dermatovenerologist (co-author WE) from Bahir Dar University. The training aimed to standardise characterisation of disease manifestations and minimise inter-observer bias in diagnosing scabies. Additionally, during the first few days of the field work, data collectors sent every participant identified as a scabies patient to the dermatologist. At times when there were disagreements in diagnosis, WE discussed the case with the data collectors and the consensual diagnosis was taken.

Sample size

We considered an average household size of rural communities 4 and intraclass correlation coefficient of 0.7 (33) to calculate a design effect of 3.1. Including 15% nonparticipation rate we

For peer review only - http://bmjopen.bmj.com/site/about/guidelines.xhtml

needed to involve 1210 people to detect 33% scabies prevalence with 5% precision. This sample size is also sufficient to fit multi-level logistic regression models with >10 variables while maintaining an events per variable ratio of >10.

Data processing and analysis

An electronic template was prepared using *Epidata V.3.01* (EpiData Association, Odense) statistical software to enter data from the paper-based questionnaire. Data was later exported to *Stata 14.0* (StataCorp LLC, Texas) for analysis. Summary measures were calculated for categorical data (frequency and percentage) and continuous data (mean and SD or median and IQR). Study households were categorised into wealth quintiles factoring in ownership of fixed household assets. Principal Component Analysis was used to generate components which best captured variability in wealth between the study households. Twenty-six binary variables of household asset and livestock ownership (including access to infrastructure) were included in the analysis (online supplemental table S2) and no significant correlation was observed among these covariates. Twenty-two principal components were generated with varimax rotation, and the first component, which had the highest *eigen value,* was used to generate the wealth index.

The dataset was hierarchical at two levels (i.e., household and individual household member), and there was a high possibility for observations among members of the same household to be correlated. The intra-class correlation coefficient (ICC) was used to characterise the level of correlation between observations from the same household. The ICC was 0.88 (95% CI 0.79–0.94), indicating a significant cluster effect between members of the same study household, justifying the multi-level modelling approach. We thus fitted multi-level logistic regression models to calculate the independent effects of individual and household level predictors on the odds of developing scabies. The models included individual participant level variables (i.e., age, sex, marital status, level of education, occupation, contact history with a person exhibiting signs of scabies, bathing, and participating in the last round of MDA) and three household level variables (i.e., household size, presence of domestic animals in the homestead, and household wealth) and a random effect for household.

We applied the ***melogit*** stata command to fit the models and calculate adjusted Odds Ratio (aOR), with 95% CI to determine the strength and direction of association between the dependent and the predictor variables. Variable inflation factors (VIFs) were calculated to detect multicollinearity among the predictor variables, but none were dropped from the model as all the VIF values were less than 5.

For peer review only - http://bmjopen.bmj.com/site/about/guidelines.xhtml

Patient and public involvement

People affected by podoconiosis, a non-infectious lymphoedema caused by prolonged exposure of the feet to irritant soil, another heavily neglected tropical condition, were involved in the development of the funding proposal supporting this study. However, at that point in time (2016), there was no scabies patient association in existence in Ethiopia. Subsequent proposals have included input from people affected by scabies, through proposal development workshops and a newly established Community of Practice.

Ethical considerations

Permission was sought from the Ministry of Health (MoH), Amhara regional health Bureau, and local administrative heads before commencing field work. Informed consent was requested and received from all adult participants, and assent from minors 15–17y. Minors <15y participated in the study once their parents or guardians gave consent. Participants diagnosed with scabies or other diseases were referred to the nearest health center for further investigation and treatment.

Results

Participants

Across six *kebeles*, a total of 1437 people (797 females, 640 males) out of 1833 registered members of the 381 study households participated. Three hundred ninety-six people were excluded after data collectors reported failure to find them after three subsequent visits to their households (online supplemental figure S2). All members of the selected households approached for recruitment consented to participate. This may surprise some readers. However, high consent rates are not uncommon in medical research in rural Ethiopian communities (34, 35). This may be explained by the community's respect for and trust of health workers and the frequent routine contact communities have with HEWs when they are doing outreach (36).

Median age was 19y (10-37) (table 1). Almost half the participants (49.5%) were ≤18y, 30.3% were 19-40y, but, broadly in line with Ethiopia's demographics (34), 20.3% were ≥41y (compared to 16% of the national population). Most did not have a formal education (59.7%) and only 7% of participants had secondary level or above education. The most common occupational group was farming (52.2%), followed by being a student (47.8%). Most participants ≥16y were married (38.8% of total participants). The number of households with a household size of <5 (49.2%) and ≥5 (50.8%) was similar. Most study households (70.6%)

For peer review only - http://bmjopen.bmj.com/site/about/guidelines.xhtml

always used surface water for any purpose. 26.6% of participants reported bathing less than weekly, 54.9% reported bathing 1-2 times weekly, and 18.5% ≥3 times weekly. 13.9% of interviewees stated their clothes were washed less than once per week, 77.7% 1-2 times weekly, and 8.5% ≥3 times weekly. Median time for a round trip to water source was 20 minutes.

Scabies cases

Scabies prevalence was 13.4% (95% CI 11.8%-15.5%). The highest age specific prevalence was among people aged 11 to 18 years 17.1% (95%CI 13.1%-21.0%) and the lowest among those between 31 and 40 years 6.7% (95%CI 2.9%-10.6%). The prevalence among people in the extremes of age: 0 to 10 years and people older than 41 years was 13.4% (95%CI 9.9%-16.9%) and 12.4% (95%CI 8.6%-16.2%), respectively. The socioeconomic characteristics and scabies diagnoses of those examined are provided in table 1. One-hundred and ninety-two participants had scabies (101 females, 91 males) (online supplemental table S3). Participants with clinical scabies were younger (median16y, IQR 10-30y) than those without signs and symptoms (median 20y, IQR 10-38y). Those with no scabies manifestations were evenly split between households with <5 and ≥5 people (respectively: 707, 49.2%; 730, 50.8%), whereas a markedly higher proportion of scabies cases lived in households with ≥5 people (122, 63.5%) than <5 (70, 36.5%). Stated frequency of clothes washing and personal bathing were similar between the scabies cases and those without manifestations.

Clinical presentations of identified scabies cases (n=192) are summarised in supplementary (online supplemental table S4). Vesicles were the most frequently found clinical signs (67.7%). The trunk and head were the most (62.0%) and least (3.6%) involved body parts. Nearly all individuals diagnosed with scabies (184, 95.8%) reported experiencing itch and the majority (165, 85.9%) stated they had a contact history with a presumed scabies case, most stating they believed they had become infested due to contact with a household member (146, 80.7%). Out of the 192 clinical scabies cases, only 62 (32.3%) reported seeking care by the time the data was collected. Of those that did, the majority (40, 64.5%) reported seeking care from a health facility, while a smaller proportion had carried out self-treatment (14, 22.6%). Three (4.8%) said they had used holy water as a remedy. The median number of days between the onset of symptoms and seeking treatment was 20 (IQR 7-30) (online supplemental table S5). Twenty-seven participants said they sought treatment later than this, giving the following reasons: twenty-one (77.8%) because they assumed the signs and symptoms would disappear on their own, three (11.1%) due to lack of time to seek care, two (7.1%) believed medical expenses would be high, and two (7.1%) because they considered the symptoms mild.

For peer review only - http://bmjopen.bmj.com/site/about/guidelines.xhtml

Community knowledge and attitudes about scabies

The knowledge and attitudes of adult study participants are summarised in tables 2 and 3. Two thirds (n=500) reported they had heard about scabies. When asked how they believed scabies was transmitted, most stated beliefs in line with established scientific knowledge: prolonged physical contact (57.6%); sharing clothes and bedding with a scabies patient (51.4%); sharing a sleeping space with a scabies patient (42.4%). However, some stated modes of transmission not in line with scientific understanding: via contaminated water (10.0%); via blood contact (4.0%); from the soil (2.8%). Most said they wanted to know more about the disease, with a higher proportion interested among the scabies cases. When asked to choose from a list, most (93%) selected body creams and anti-scabies tablets as scabies treatments, while small minorities chose holy water (4.4%), traditional treatment (home remedies and traditional herbs) (3.4%), or prayer (1.4%). 56.3% of adults without scabies said they thought any community member could acquire scabies (table 2). Nearly all (441, 95.7%) said they would seek care from health care facilities if they developed scabies (table 3). 213 (48.3%) reported they would feel nothing emotionally if they developed scabies, but 116 (26.3%) said they would feel shame, and 113 (25.6%) fear. When asked to choose from a list of potential attitudes towards scabies patients, a similar proportion chose a response stating, 'they felt compassion and wanted to help' and 'they feared they would be infected by them' (respectively, 115, 56.4% and 104, 51.0%). 166 (81.4%) chose a response stating, 'they believed their community would prefer to avoid scabies patients', and 46 (22.5%) that 'people would appear friendly but wish to avoid them' (table 3).

Odds of infestation

Table 4 gives the results of the multi-level logistic regression analysis of determinants of scabies infestation. The odds of scabies infestation was lower among females (aOR=0.5, 95% CI 0.3–0.8), and people from households of five or more had higher odds of infestation (aOR=3.5, 95% CI 1.2 - 10.2). The following were not predictive of scabies diagnosis: occupation; education; marital status; age; frequency of bathing; taking ivermectin tablets during prior MDA; keeping domestic animals in the homestead; household wealth.

For peer review only - http://bmjopen.bmj.com/site/about/guidelines.xhtml

Discussion

Scabies prevalence in Ayu Guagusa district was high. Vesicles, excoriations, and papules, often involving the trunk, were the most frequently observed signs, and nearly all participants complained of generalized itch. Only around a third of clinical scabies cases had sought care, with the median number of days before seeking care for the minority who did being 20 days. More than two-thirds had heard about scabies, and friends and family were the commonest sources of scabies-related information. Most believed scabies was transmitted through prolonged physical contact with a scabies patient and that it could be treated with drugs. In the multi-level logistic regression model being from households of size ≥5 people increased odds of scabies infestation but female sex was associated with lower odds of scabies.

There are few studies on scabies in Africa, including Ethiopia, and population-based studies employing primary data are even rarer (20, 23, 25, 37, 38). Scabies prevalence estimates for different parts of Ethiopia range from 2.5 to 33.5% (23). However, since most of these studies were conducted either in areas of scabies outbreaks or were secondary analyses on data related with scabies outbreak response activities, their prevalence estimates may not be representative of the remaining parts of the country (23, 39, 40). In our study, estimated scabies prevalence was 13.4%. We believe this high community prevalence is likely to be, in part, related to the wider ongoing scabies outbreak across Amhara region, first reported in 2015 (26). The prevalence is comparable to studies in southern Ethiopia (25, 37) but lower than estimates from studies elsewhere in the northern parts of the country (23, 26, 41). This may be a secondary impact of prior single dose ivermectin-only onchocerciasis MDA that had taken place 8-months before the current study.

Only around a third of those diagnosed with scabies had reported seeking care of any kind by the time this data was collected. Similar poor health seeking behaviour have been reported in other studies (18, 22). Not seeking care early because of low perceived severity of manifestations, was also found in a study in a Brazilian urban slum (18). Amongst those who sought care, more than two-thirds reported attending a primary health care facility. Even though we could not rule out the effect of social desirability bias (since the data collectors were health workers), the improving physical and financial access to primary health services could have made health care facilities the preferred source of care (42). However, services at these primary health care facilities may benefit from enhancing quality of care and employing teledermatology (43).

For peer review only - http://bmjopen.bmj.com/site/about/guidelines.xhtml

Participants reported to the first author that frequent stock-outs of the commonly used cream (permethrin) in many health centres discouraged their attendance. Self-treatment was the second most frequently mentioned source of care. From our anecdotal field observation, people applied local herbs on the lesions.

Nearly two thirds of the study population had heard about scabies, primarily from family and friends. The now pervasive awareness about scabies is, we suspect, mainly a result of its occurrence as an outbreak. More than half of the study participants in both groups (i.e., clinical scabies and no manifestations of scabies) correctly identified 'prolonged physical contact with a scabies patient' as a mode of scabies transmission. There remains room for educational interventions, as over a third in each group did not identify this mode of transmission. Local health worker teaching on scabies has focused heavily on personal hygiene. However, reported frequency of bathing was not predictive in our study, and experimental and epidemiological evidence from elsewhere indicates personal body washing is probably unrelated to scabies prevalence (44). A better focus would be to prevent prolonged physical contact with presumed scabies cases (e.g., temporary separation of sleeping arrangements for symptomatic individuals), and to treat all contacts once a scabies case is identified. Most participants believed there is treatment for scabies and a comparable number wanted to know more about scabies in general. This presents an opportunity for effective behavioural change communications. Even though we did not use a validated tool to measure attitude, a subtle stigma was associated with scabies patients. This finding was consistent with Trettin *et al.*, where scabies patients reported shame and fear of stigma as scabies was perceived to be associated with poor hygiene and poverty (45, 46). Arguably, the present focus on personal hygiene in teachings surrounding scabies may risk contributing to such social stigma against scabies patients.

High scabies prevalence is reported among children in high scabies burden settings, and in a global systematic review by Romani *et al.* (47) prevalence was lower in adults than in children in all relevant studies. In our study, though the association between age and scabies was not statistically significant, the majority of the scabies cases were still among people aged <18y. Household size was a strong predictor; odds of scabies infestation among people living in households with ≥5 members (a household size larger than the national average) (48) was high. Similarly, Hegazy *et al.* (49) reported that people from households of high crowding index had higher odds of scabies infestation in rural Egypt. Since prolonged physical contact is required for effective scabies transmission, high host density both in households and institutional settings facilitates transmission (50).

For peer review only - http://bmjopen.bmj.com/site/about/guidelines.xhtml

Conclusion

Scabies prevalence in Ayu Guagusa district is high and crosses the recommended threshold to initiate preventive chemotherapy (51). MDA need to be initiated as the outbreak can no longer be controlled through standard care (52). Ivermectin MDAs for scabies were first trialled in Oceania in the late 1990s and have proven to be effective at reducing community prevalence (53). Ideally two doses of oral ivermectin (200 μg/kg) should be given 7-14 days apart, with alternative scabicides provided to whom ivermectin is presently not recommended (children weighing <15 kg or <90 cm height; pregnant women; women breastfeeding in the first week). MDA rounds should have a minimum of 80% population coverage and conducted annually for 3-5 years, with a stopping threshold of 2% community prevalence (51). Accompanying such MDAs with educational interventions may also help to: (1) increase awareness about modes of scabies transmission; (2) address the subtle stigma attached to scabies; and (3) support communities to modify environmental circumstances that facilitate transmission.

Data availability statement

Raw data are available upon request to the corresponding author (RY).

Ethics statements

Patient consent for publication

Not applicable.

Ethics approval

The study involved human participants and ethics approval was obtained from Addis Ababa University Institutional Review Board (IRB) (AAUMF 03-008) and Brighton and Sussex Medical School (BSMS) Research Governance and Ethics Committee (RGEC) (ER/BSMS9G1Z/1).

Author's contributions

This study was carried out as part of the work of the NIHR Global Health Research Unit on Neglected Tropical Diseases Phase 1 at Brighton and Sussex Medical School. For clarity we detail contributions using the CRediT Contributor Roles Taxonomy (https://casrai.org/credit/) and provide employment and disciplinary descriptions. Conceptualization: JM, AF, JAC, GD. Analysis: RY, JM. Investigation: RY, AT, WE. Methodology: RY, JM, AF, JAC, SB, CIJ, GD. Supervision: JM, AF, JAC, GD. Writing – original draft: RY, JM. Visualization: JM, AT. Writing – review & editing: RY, JM, AF, JAC, AT, SB, CIJ, WE, GD.

For peer review only - http://bmjopen.bmj.com/site/about/guidelines.xhtml

Appendices

Supplementary Files accompanying this paper include: study location map; study diagram; author reflexivity statement; tables detailing fixed asset variables used to categorise households into wealth quintiles, scabies manifestations among clinically diagnosed cases, care seeking characteristics of participants who sought care for scabies suggestive symptoms, and distribution of participants by age and sex.

Acknowledgments

We thank the field team, health care workers from Gimjabet hospital, for conducting data collection and Dr. Vasso Anagnostopoulou for supporting data management activities. Awi Zone and Ayu Guagusa District Health office administration provided invaluable background information about the institutions in the health system and for facilitating the field work. We are also grateful to the participants who welcomed our field team into their homes and gave their time for the interview.

Funding statement

This research was financially supported by the National Institute for Health and Care Research (Grant number 16/136/29).

Competing interest

None.

For peer review only - http://bmjopen.bmj.com/site/about/guidelines.xhtml

References

1. World Health Organization. Ending the neglected to attain the sustainable development goals. A road map for neglected tropical diseases 2021–2030. Geneva. 2020. https://www.who.int/publications/i/item/9789240010352 (accessed 01 May 2022).
2. Engelman D, Martin DL, Hay RJ, Chosidow O, McCarthy JS, Fuller LC, et al. Opportunities to investigate the effects of ivermectin mass drug administration on scabies. *Parasit Vectors*. 2013;6:106.
3. Nagpal S, Sinclair D, Garner P. Has the NTD Community Neglected Evidence-Based Policy? *PLoS Negl Trop Dis*. 2013;7(7):1-3.
4. Fuller LC. Epidemiology of scabies. *Curr Opin Infect Dis*. 2013;26:123-126.
5. Hay RJ, Steer AC, Engelman D, Walton S. Scabies in the developing world—its prevalence, complications, and management. *Clin Microbiol Infect*. 2012;18(4):313–323.
6. Chosidow O. Scabies. *N Engl J Med*. 2006;354(16):1718-1727.
7. Mellanby K. Scabies in 1976. *R Soc Health J*. 1977;97(1):32-36.
8. Hardy M, Engelman D, Steer A. Scabies: A clinical update. *Aust Fam Physician*. 2017;46(5):264-268.
9. Walton SF, Currie BJ. Problems in Diagnosing Scabies, a Global Disease in Human and Animal Populations. *Clin Microbiol Rev*. 2007;20(2):268-279.
10. Zaidi AKM, Thaver D, Ali SA, Khan TA. Pathogens Associated With Sepsis in Newborns and Young Infants in Developing Countries. *Pediatr Infect Dis J*. 2009;28:10-18.
11. Gao Z, Zhao H, Xia Y, Gan H, Xiang Z. Clinical Characteristics and Etiologic Analysis of Scabies-Associated Glomerulonephritis. *Intern Med*. 2015;5(4):1-5.
12. Engelman D, Fuller LC, Steer AC. Consensus criteria for the diagnosis of scabies: A Delphi study of international expert. *PLoS Negl Trop Dis*. 2018;12(5):1-9.
13. Karimkhani C, Colombara DV, Drucker AM, Norton SA, Hay R, Engelman D, et al. The global burden of scabies: a cross-sectional analysis from the Global Burden of Disease Study 2015. *Lancet Infect Dis*. 2017;17:1247-1254.
14. Mellanby K. The development of symptoms, parasitic infection and immunity in human scabies. *Parasitology*. 1943;35(4):197-206.
15. Cassell JA, Middleton J, Nalabanda A, Lanza S, Head MG, Bostock J, et al. Scabies outbreaks in ten care homes for elderly people: a prospective study of clinical features, epidemiology, and treatment outcomes. *Lancet Infect Dis*. 2018;18:894-902.
16. Richardson NA, Cassell JA, Head MG, Lanza S, Schaefer C, Walker SL, et al. Scabies outbreak management in refugee/migrant camps across Europe 2014-17: a retrospective qualitative interview study of healthcare staff experiences and perspectives. *medRxiv*; 2021 (accessed May 01, 2021).
17. Feldmeier H, Jackson A, Ariza L, Calheiros CM, Soares Vde L, Oliveira FA, et al. The epidemiology of scabies in an impoverished community in rural Brazil: presence and severity of disease are associated with poor living conditions and illiteracy. *J Am Acad Dermatol*. 2009;60(3):436-443.

For peer review only

18. Heukelbach Jr, Haeff Ev, Rump B, Wilcke T, Ce´sar Rm, Moura Si, et al. Parasitic skin diseases: health care-seeking in a slum in north-east Brazil. *Trop Med Int Health*. 2003;8(4):368-373.

19. Boateng LA. Healthcare-seeking behaviour in reporting of scabies and skin infections in Ghana: A review of reported cases. *Trans R Soc Trop Med Hyg*. 2020(0):1-9.

20. Figueroa JI, Fuller LC, Abraha A, Hay RJ. Dermatology in southwestern Ethiopia: rationale for a community approach. *Int J Dermatol*. 1998;37(10):752-758.

21. Vincente SL, Kearns T, Connors C, Cameron S, Carapetis J, Andrews R. Community Management of Endemic Scabies in Remote Aboriginal Communities of Northern Australia: Low Treatment Uptake and High Ongoing Acquisition. *PLoS Negl Trop Dis*. 2009;3(5):1-8.

22. Lopes MJ, Silva ETd, Ca J, Gonçalves A, Amabelia, Rodrigues, et al. Perceptions, attitudes and practices towards scabies in communities on the Bijagós Islands, Guinea-Bissau. *Trans R Soc Trop Med Hyg*. 2020;114(1):49-56.

23. Enbiale W, Ayalew A. Investigation of a Scabies Outbreak in Drought-Affected Areas in Ethiopia. *Trop Med Infect Dis*. 2018;3(114):1-9.

24. Yassin ZJ, Dadi AF, Nega HY, Derseh BT, Asegidew W. Scabies Outbreak Investigation among "Yekolo Temaris" in Gondar Town, North Western Ethiopia, November 2015. *Electronic Journal of Biology*. 2017;13(3):203-209.

25. Sara J, Haji Y, Gebretsadik A. Scabies Outbreak Investigation and Risk Factors in East Badewacho District, Southern Ethiopia: Unmatched Case Control Stud. *Dermalogy Research and Practice*. 2018;2018:1-10.

26. Accorsi S, Barnabas GA, Farese P, Padovese V, Terranova M, Racalbuto V, et al. Skin disorders and disease profile of poverty: analysis of medical records in Tigray, northern Ethiopia, 2005-2007. Trans R *Soc Trop Med Hyg*. 2009;103(5):469-475.

27. Figueroa JI, Hawranek T, Abraha A, J.Hay R. Prevalence of skin diseases in school children in rural and urban communities in the Illubabor province, south-western Ethiopia: a preliminary survey. *J Eur Acad Dermatol Venereol*. 1997;9:142-148.

28. Walker SL, Lebas E, Sario VD, Deyasso Z, Doni SN, Marks M, et al. The prevalence and association with healthrelated quality of life of tungiasis and scabies in schoolchildren in southern Ethiopia. *PLoS Negl Trop Dis*. 2017;11(8):1-11.

29. Yirgu R. From quantification to control: a population-based study to measure the secondary impact of ivermectin-based mass drug administration aimed to control onchocerciasis on the prevalence of scabies in northern Ethiopia 2023.

30. Federal Democratic Republic of Ethiopia Ministry of Health. Health Sector Development Programme IV.2000. http://www.nationalplanningcycles.org/sites/default/files/country_docs/Ethiopia/ethiopia_hsdp_iv_final_draft_2010_-2015.pdf2010 (accessed 04 May 2022).

For peer review only - http://bmjopen.bmj.com/site/about/guidelines.xhtml

31. Federal Democratic Repiblic of Ethiopia Population Census Commission. Summary and statical report of the 2007 populatin and housing census Addis Ababa: United Nations Population Fund, 2008. https://www.statsethiopia.gov.et/census-2007-2/ (accessed 07 May 2022).
32. Central Statistics Agency (CSA). Ethiopia Socioeconomic Survey. 2019. https://www.statsethiopia.gov.et/ (accessed 04 May 2022).
33. Kositz C, Drammeh M, Vasileva H, Houghton J, Ashall J, D'Alessandro U, et al. Effects of ivermectin mass drug administration for malaria vector control on ectoparasites and soil-transmitted helminths: a cluster randomized trial. *Int J Infect Dis*. 2022;125:258-264.
34. Regasa MT, Hinkosa L, Besho M, Bekele T, Bekuma TT, Tsegaye R, et al. Predictors of preterm birth in Western Ethiopia: A case control study. *PLoS One*. 2021;16(4):e0247927.
35. Legesse A, Adisalem D. Knowledge, Attitude and Practice toward legalization of abortion among female students of Sabiyan secondary and preparatory school, Dire Dawa, Eastern Ethiopia, 2018. A descriptive cross-sectional study. *Asian Journal of Medical Research*. 2020;9(2).
36. Assefa Y, Gelaw YA, Hill PS, Taye BW, Van Damme W. Community health extension program of Ethiopia, 2003–2018: successes and challenges toward universal coverage for primary healthcare services. *Globalization and Health*. 2019;15(1):24.
37. Wochebo W, Haji Y, Asnake S. Scabies outbreak investigation and risk factors in Kechabira district, Southern Ethiopia: unmatched case control study. *BMC Res Notes*. 2019;12(305):1-6.
38. Dagne H, Dessie A, Destaw B, Yallew WW, Gizaw Z. Prevalence and associated factors of scabies among schoolchildren in Dabat district, northwest Ethiopia, 2018. *Environ Health Prev Med*. 2019;24(67):1-8.
39. Azene AG, Aragaw AM, Wassie GT. Prevalence and associated factors of scabies in Ethiopia: systematic review and Meta-analysis. *BMC Infect Dis*. 2020;20(1):1-10.
40. Enbiale W, Baynie TB, Ayalew A, Gebrehiwot T, Getanew T, Ayal A, et al. "Stopping the itch": mass drug administration for scabies outbreak control covered for over nine million people in Ethiopia. *Journal of infection in developing countries.* 2020;14(6.1):28S-35S.
41. Enbiale W, Ayalew A, Gebrehiwot T, Mulu Y, Azage M, Zachariah R, et al. Does mass drug administration for community-based scabies control works? The experience in Ethiopia. *Journal of infection in developing countries.* 2020;14(6.1):78s-85s.
42. Demissie B, Negeri KG. Effect of Community-Based Health Insurance on Utilization of Outpatient Health Care Services in Southern Ethiopia: A Comparative Cross-Sectional Study. *Risk Management and Healthcare Policy*. 2020(13):141-143.
43. Joshi TP, Ren V. Teledermatology in the Control of Skin Neglected Tropical Diseases: A Systematic Review. *Dermatol Pract Concept.* 2021;11(4):e2021130.
44. Middleton J, Cassell JA, Jones CI, Lanza S, Head MG, Walker SL. Scabies control: the forgotten role of personal hygiene-Authors' reply. *The Lancet Infectious Diseases.* 2018;18(10):1068-1069.

For peer review only - http://bmjopen.bmj.com/site/about/guidelines.xhtml

45. Trettin B, Lassen JA, Andersen F, Agerskov H. The journey of having scabies–A qualitative study. *Journal of Nursing Education and Practice*. 2019;9(2):1-9.
46. Jin-gang A, Sheng-xiang X, Sheng-bin X, Jun-min W, Song-mei G, Ying-ying D, et al. Quality of life of patients with scabies. *Journal of the European Academy of Dermatology and Venereology.* 2010;24:1187-1191.
47. Romani L, Whitfeld MJ, Koroivueta J, Kama M, Wand H, Tikoduadua L, et al. Mass Drug Administration for Scabies Control in a Population with Endemic Disease. *The new England Journal of Medicine.* 2015;373(24):2305-2313.
48. Central Statistical Agency (CSA) Ethiopia and ICF. Ethiopia Demographic and Health Survey 2016. Addis Ababa Ethiopia and Rockville, Maryland, USA; 2016. https://evaw-global-database.unwomen.org/en/countries/africa/ethiopia/2016/demographic-and-health-survey-2016 (accessed 06 May 2022).
49. Hegazy AA, Darwish NM, Abdel-Hamid IA, Hammad SM. Epidemiology and control of scabies in an Egyptian village. *Int J Dermatol*. 1999;38:291-295.
50. Jo Middleton, Jackie A Cassell, Steven Walker. Scabies management in institutions. In: Chosidow O, Fischer K, eds. *Scabies*. Berlin: Springer, In Press.
51. World Health Organization. WHO Informal Consultation on a Framework for Scabies Control. Manila; 2019. https://www.who.int/publications/i/item/9789240008069 (accessed 26 May 2022).
52. Hotez PJ. Ten failings in global neglected tropical diseases control. *PLoS Negl Trop Dis*. 2017;11(12):e0005896.
53. Middleton J. Can ivermectin mass drug administrations to control scabies also reduce skin and soft tissue infections? Hospitalizations and primary care presentations lower after a large-scale trial in Fiji. *The Lancet Regional Health – Western Pacific*. 2022;22.

For peer review only

For peer review only - http://bmjopen.bmj.com/site/about/guidelines.xhtml

TABLES

Table 1 Socioeconomic characteristics of the study sample (n=1437)

Variables	Categories	Overall (n=1437) n (%)	Scabies diagnosis: No scabies manifestations (n=1245) n (%)	Scabies diagnosis: Clinical scabies (n=192) n (%)
**Sex**	Male	640 (44.5)	539 (43.3)	101 (52.6)
	Female	797 (55.5)	706 (56.7)	91 (47.4)
**Age in years**	≤10	365 (25.4)	316 (25.4)	49 (25.5)
	11–18	346 (24.1)	287 (23.1)	59 (30.7)
	19–40	435 (30.3)	387 (31.1)	48 (25.0)
	≥41[π]	291 (20.3)	255 (20.5)	36 (18.8)
	Median age (IQR)	19 (10-37)	20 (10-38)	16 (10-30)
**Level of education* (n=1422)**	No formal education	849 (59.7)	745 (60.6)	104 (54.2)
	Primary education	474 (33.3)	401 (32.6)	73 (38.0)
	Secondary and above	99 (7.0)	84 (6.8)	15 (7.8)
**Occupation (n=1412)**	Farmer	737 (52.2)	650 (53.3)	87 (45.3)
	Student	675 (47.8)	570 (46.7)	105 (54.7)
**Marital status[#]**	Not married	228 (15.9)	204 (16.4)	24 (12.5)
	Married	558 (38.8)	487 (39.1)	71 (37.0)
	<16y	651 (45.3)	554 (44.5)	97 (50.5)
**Household size**	<5 people	707 (49.2)	637 (51.2)	70 (36.5)
	≥5 people	730 (50.8)	608 (48.8)	122 (63.5)
**Contact history with symptomatic person**	No	947 (65.9)	939 (75.4)	8 (4.2)
	Yes	490 (34.1)	306 (24.6)	184 (95.8)
**Took pill during the last MDA**	No	338 (23.5)	274 (22.0)	64 (33.3)
	Yes	1099 (76.5)	971 (78.0)	128 (66.7)
**Domestic animals kept in the homestead**	No	202 (14.1)	171 (13.7)	31 (16.2)
	Yes	1235 (85.9)	1074 (86.3)	161 (83.9)
**Household wealth**	Lowest	291 (20.3)	266 (21.4)	25 (13.0)
	Second	277 (19.3)	240 (19.3)	37 (19.3)
	Middle	293 (20.4)	255 (20.5)	38 (19.8)
	Fourth	293 (20.4)	261 (20.9)	32 (16.7)
	Highest	283 (19.7)	223 (17.9)	60 (31.3)
**Main source of water for any purpose[‡]**	Public tap	181 (12.6)	139 (11.3)	42 (21.9)
	Dug well	405 (28.2)	355 (28.5)	50 (26.0)
	Spring water	850 (59.2)	746 (59.9)	104 (54.2)
**Frequency of bathing per week**	<1	382 (26.6)	326 (26.2)	56 (29.2)
	1-2	789 (54.9)	688 (55.3)	101 (52.6)
	≥3	266 (18.5)	231 (18.6)	35 (18.2)
**Frequency of washing clothes per week**	<1	199 (13.9)	174 (13.9)	25 (13.0)
	1-2	1116 (77.7)	967 (77.7)	149 (77.6)
	≥3	122 (8.5)	104 (8.4)	18 (9.4)

[π] People >40 y comprises just 16% of the Ethiopian population; *This category comprised people with no formal education and children younger than school age; [#] Marital status of participants was only asked of those ≥16y. [‡]Total scores exceed 100% as individual participants could select multiple response categories.

For peer review only - http://bmjopen.bmj.com/site/about/guidelines.xhtml

Table 2 Knowledge of adult study participants about scabies (n=500)

Variable	Categories	Overall n (%) *	Scabies diagnosis among adults	
			No scabies Manifestations (n=430) n (%)	Clinical scabies (n=70) n (%)
**From what sources first heard about scabies**	Family and friends	320 (64.0)	269 (62.6)	51 (72.9)
	Health care providers	71 (14.2)	65 (15.1)	6 (8.6)
	Health extension workers	48 (9.6)	9 (2.9)	39 (55.7)
	Mass media	44 (8.8)	33 (7.7)	11 (15.7)
	Religious leaders	18 (3.6)	18 (4.2)	0
	Not collected	44 (8.8)	42 (9.8)	2 (2.9)
**What are the manifestations of scabies?**	Itch	449 (89.8)	382 (88.8)	67 (95.7)
	Rash	242 (48.4)	187 (43.5)	55 (78.6)
	Skin crust	24 (4.8)	17 (3.9)	7 (10.0)
**How is scabies transmitted?**	Prolonged physical contact with a scabies patient	288 (57.6)	245 (56.9)	43 (61.4)
	Sharing clothing and bedding with a scabies patient	257 (51.4)	216 (50.2)	41 (58.6)
	Sharing sleeping space with scabies patient	212 (42.4)	175 (40.7)	37 (52.9)
	From contaminated water	50 (10.0)	49 (11.4)	1 (1.4)
	Blood contact with a scabies patient	20 (4.0)	18 (4.2)	2 (2.9)
	From the soil	14 (2.8)	12 (2.8)	2 (2.9)
	No response	58 (11.6)	49 (11.4)	9 (12.9)
**Is scabies treatable?**	Yes	450 (90.0)	387 (90.0)	63 (90.0)
	No	45 (9.0)	39 (9.1)	6 (8.6)
	No response	5 (1.0)	4 (1.0)	1 (1.4)
**What is the treatment for scabies?**	Body creams and tablets	469 (93.8)	404 (93.9)	65 (92.8)
	Holy water	22 (4.4)	21 (4.9)	1 (1.4)
	Traditional treatment	17 (3.4)	16 (3.7)	1 (1.4)
**Who can catch scabies?**	Any one	282 (56.3)	235 (54.5)	47 (67.1)
	Poor people	53 (10.6)	42 (9.7)	11 (15.7)
	Rich people	14 (2.8)	11 (2.6)	3 (4.3)
	Homeless people	24 (4.8)	20 (4.6)	4 (5.7)
	Urban people	14 (2.8)	12 (2.8)	2 (2.9)
	Rural people	49 (11.1)	38 (10.1)	11 (16.9)
	Commercial sex workers	22 (4.4)	19 (4.4)	3 (4.3)
	Prisoners	22 (4.4)	18 (4.2)	4 (5.7)
	No response	44 (8.8)	39 (9.1)	5 (7.1)
**Do you wish to know more about scabies?**	Yes	435 (87.0)	371 (86.3)	64 (91.4)
	No	65 (13.0)	59 (13.7)	6 (8.6)

*Total scores may exceed 100% for some questions when individual participants could select more than one response categories.

For peer review only - http://bmjopen.bmj.com/site/about/guidelines.xhtml

Table 3 Attitude of adult study participants without scabies symptoms towards scabies (n=441)

Question	Categories	n (%) *
**Do you think you can acquire scabies?**	Yes	265 (61.0)
	No	169 (38.3)
	No response	7 (1.6)
**What would you feel if you developed scabies?**	Nothing	213 (48.3)
	Shame	116 (26.3)
	Fear	113 (25.6)
	Disappointment	68 (15.4)
	Surprise	16 (3.6)
	No response	13 (2.9)
**What would you do if you developed scabies?**	Seek care from health care facilities	422 (95.7)
	Use traditional medicine	2 (0.5)
	Do nothing because it improves on its own	2 (0.5)
	No response	16 (3.6)
**What is your feeling towards scabies patients? (n=204) ****	I feel compassion and wish to help	115 (56.4)
	I fear they may infect me	104 (51.0)
	I do not feel anything	66 (32.4)
	I feel compassion but prefer to stay away	40 (19.4)
	No response	6 (2.9)
**How does the community treat scabies patients? (n=204) ****	Most people prefer to avoid scabies patients	166 (81.4)
	People help and support them	78 (38.2)
	People appear friendly but they wish they avoided them	46 (22.5)
	No response	25 (22.3)

*All items in this table were multiselect and participants could give more than one response for each variable, and this might cause the cumulative score of some variables to exceed the total number of participants. **Calculated out of 204 participants who have knowledge of a scabies patient.

For peer review only - http://bmjopen.bmj.com/site/about/guidelines.xhtml

Table 4 Multi-level logistic regression analysis results of determinants of scabies infestation (Number of households N=381; number of participants N=1403; number of cases 192).

Variable	Category	aOR	95% CI
**Sex**	Male (ref)		
	Female	0.5	0.3 - 0.8
**Age (years)**	≤ 17 (ref)		
	18 - 40	0.5	0.0 - 4.4
	≥ 41	0.4	0.0 - 4.2
**Occupation**	Farmer (ref)		
	Student	0.9	0.3 - 3.2
**Education**	No formal education (ref)		
	Primary education	1.1	0.5 - 2.3
	Secondary and higher	2.8	0.8 - 9.9
**Marital status**	Not married (ref)		
	Married	0.8	0.1 - 7.0
	<16	1.3	0.5 - 3.8
**Frequency of bathing per week**	<1 (ref)		
	1-2 times	0.6	0.3 - 1.4
	≥3 times	1.0	0.3 - 2.9
**Took ivermectin during last MDA**	No (ref)		
	Yes	0.7	0.2 - 3.0
**Household size**	<5 people (ref)		
	≥5 people	3.5	1.2 - 10.2
**Domestic animals kept in homestead**	No (ref)		
	Yes	0.7	0.2 - 3.0
**Household wealth**	Lowest (ref)		
	Second	0.6	0.1 - 2.7
	Middle	0.8	0.2 - 4.1
	Fourth	0.8	0.2 - 4.2
	Highest	3.5	0.8 - 15.1
**Intracluster correlation coefficient (ICC, 95%CI)**		0.89	0.79 - 0.94

aOR: Adjusted odds ratio

For peer review only

Supplementary Materials

Yirgu, Middleton, Fekadu *et al*. Scabies in the Amhara region of northern Ethiopia: a cross-sectional study of prevalence, determinants, clinical presentation, and community knowledge. Submitted to *BMJ Global Health* April 2023.

Table of Contents

Figure S1. Study area and study locations

Table S1. Full questionnaire

Table S2. Fixed asset variables used to categorise households into wealth quintiles, with justifications

Figure S2. Study diagram, number of participants, and place of residence (*kebeles*)

Table S3. Distribution of participants by age and sex

Table S4. Scabies manifestations among clinically diagnosed cases

Table S5. Care-seeking characteristics of participants who sought care for scabies suggestive symptoms

For peer review only

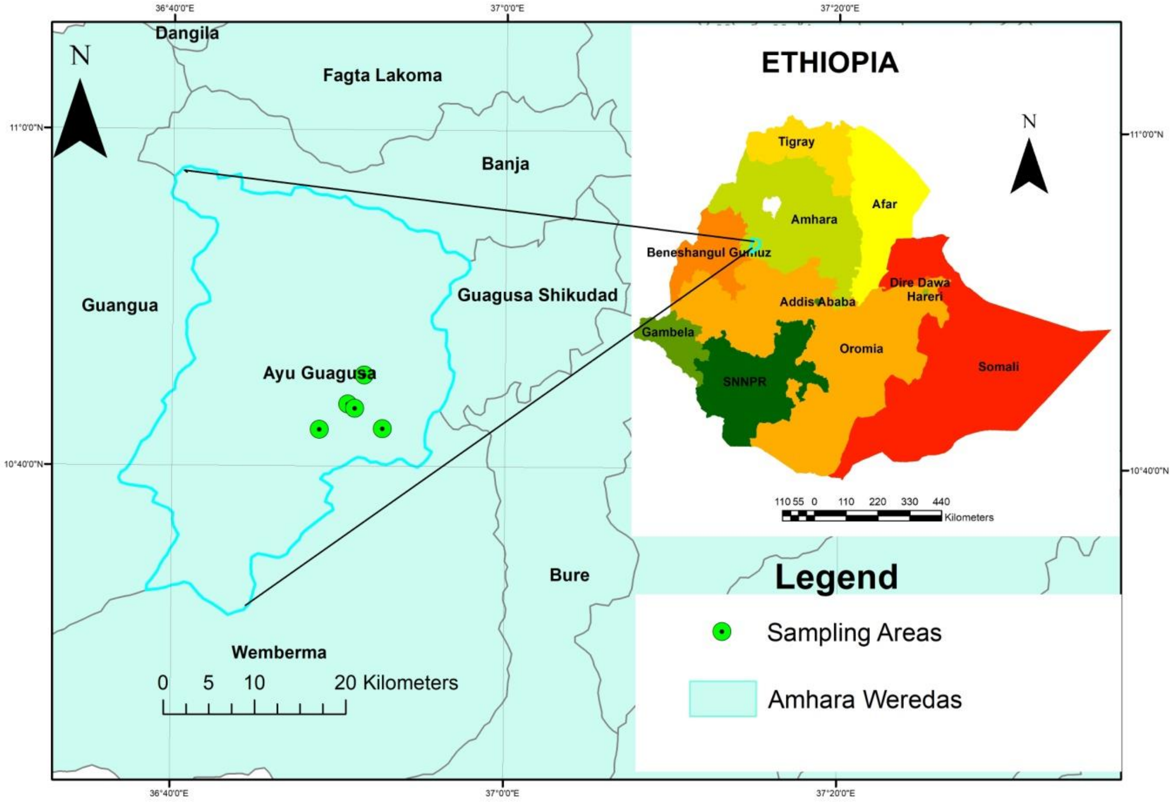

Figure S1. Study area and locations**:** Amhara Regional State; Ayu Guagusa district in Awi zone; sampled kebeles. Produced using ArcGIS version 10.8 (ESRI, Redlands, USA).

For peer review only - http://bmjopen.bmj.com/site/about/guidelines.xhtml

Table S1. Full questionnaire

Household Questionnaire

**Questionnaire.ID**		________________				
**NO**	**QUESTIONS AND FILTERS**	**CODING CATEGORIES**				
**IDENTIFICATION**<br>**Please record the following identifying information prior to beginning the interview.**						
001	**Name of the interviewer**					
002	**Profession of the interviewer**					
003	**Date and time**	Date	Month	Day	Year	
004a	**Region**	Tigray........1<br>Afar........2<br>Amhara........3<br>Oromia........4<br>Ethiopia Somali........5<br>Benishangul Gumuz........6<br>SNNPR........7<br>Gambella........8<br>Harari........9<br>Addis Ababa........10<br>Dire Dawa........11				
004b	**Zone**					
004c	**District**					
004d	**Locality (Kebele) name**					

For peer review only - http://bmjopen.bmj.com/site/about/guidelines.xhtml

Questionnaire.ID		________________________________	
**NO**	**QUESTIONS AND FILTERS**	**CODING CATEGORIES**	
004e	**Gote**		
005	**Household number**	Number ☐	
006	**Number of visits to the house hold?**	Number ☐	
007	**Respondents first name**		
008	**Relationship of the respondent to the study household?**	HH member…………………………..1<br>Neighbor……………………………….2<br>A friend of the family…………………3<br>Community leader……………………4<br>Don't know…………………………….-88<br>No response………………………….-99	

For peer review only

**SECTION 1.1 – Household Roster**<br>**I am now going to ask you a series of questions about usual members of the household**								
**HH member ID**	**Relationship to head of the HH**	**Age at the last birth day**	**Gender**	**Education**	**Marital status**	**Occupation**	**Scabies symptoms**	**Referred for verification**
	101.What is (name) relationship to (name of the head of the HH)?<br>Head…………..1<br>Wife/Husband...2<br>Son/Daughter…3<br>Son/Daughter-in-law...4<br>Grandchild….…..5<br>Parent………….6<br>Parent in law.….7<br>Brother/Sister…8<br>House help…….9<br>Other____________<br>Don't know……-88<br>No response.…-99	102.How old is (name)?	103.Is (name) male or female?<br><br>Male………1<br>Female……2	104.What is the highest level of school (name) attended? *Document the last completed grade.*<br>Never Attended .... 0<br>Primary .... 1<br>Secondary .... 2<br>Technical & vocational .... 3<br>Higher.................................. 4<br>No response……….-99	105.What is (name) marital status?<br><br><br>Married .... 1<br>Living with a partner................ 2<br>Divorced / separated............ 3<br>Widow / widower 4<br>Never Married.. .. 5<br>NA……………6<br>Don't know…-88<br>No response . . . .-99	106.What is (name) occupation?<br><br>Farmer………1<br>Government employee……2<br>Daily laborer..3<br>Merchant……4<br>Student……..5<br>Unemployed...6<br>Notapplicable..7	107.Did (name) complain itchy skin rash/papules in the past two months?<br><br>Yes………1<br>No………..2<br>No response…-99<br>Don't know-88	108. The participant is sent for verification?<br><br>Yes………1<br>No………..2

For peer review only

CHAPTER - I - HOUSEHOLD CHARACTERISTICS
SECTION-1 HOUSEHOLD CHARACTERISTICS

Section 1. 2 – Household asset

Now I would like to ask you a few questions about the asset your household owns, this information helps us to assess the contribution of household wealth to the health status of members of the household

NO	QUESTIONS AND FILTERS	CODING CATEGORIES	Skip to:
109	**Please tell me about the items your household owns. Does your household have:**<br>*Read out all types and select all that apply.*<br>*If an item is reported broken but said to be out of use only temporarily, select the item. Otherwise do not select the item.*	Electricity .... 1/0<br>A watch/clock........ 1/0<br>A radio........ 1/0<br>A television .... 1/0<br>A mobile phone .... 1/0<br>A non-mobile telephone .... 1/0<br>A refrigerator........ 1/0<br>A table .... 1/0<br>A chair........ 1/0<br>A bed with cotton/sponge/spring mattress 1/0<br>An electric mitad .... 1/0<br>A kerosene lamp/pressure lamp........ 1/0<br>A bicycle .... 1/0<br>A motorcycle/ scooter .... 1/0<br>An animal-drawn cart........ 1/0<br>A car/truck........ 1/0<br>None of the above........ -77<br>No response..……………………-99	
110a	**Does this household own any livestock, herds, other farm animals, or poultry?**<br>*These livestock can be kept anywhere, not necessarily on the homestead.*	Yes........ 1<br>No .... 0<br>No response .... -99	0 → 111a

For peer review only - http://bmjopen.bmj.com/site/about/guidelines.xhtml

110b	**How many of the following animals does this household own?**<br><br>**Zero is a possible answer. Enter -88 for do not know. Enter -99 for no response.**<br><br>*The household can keep the livestock anywhere but must own the livestock recorded here.*	Milk cows/bulls/oxen [ ]<br>Horses/donkeys/mules [ ]<br>Camels [ ]<br>Goats [ ]<br>Sheep [ ]<br>Chickens [ ]<br>Beehives [ ]	
110c	**Are any of the livestock, herds, other farm animals, or poultry kept in the homestead?**	Yes..……………………………………1<br>No..……………………………………..2<br>Don't know..…………………………-88<br>No response..………………………-99	0 → 111a
110d	**Which animals are kept in the homestead?**<br><br>*Only refer to those animals which were mentioned in 109c*	Milk cows/bulls/oxen ………………1<br>Horses/donkeys/mules……...............2<br>Camels……………………………….3<br>Goats………………………………..4<br>Sheep……………………………….5<br>Chickens…………………………….6<br>Don't know…………………………-88<br>No response……………………….-99	
**The next questions are about the source of water your household regularly use for different purposes.**			

For peer review only - http://bmjopen.bmj.com/site/about/guidelines.xhtml

111a	**Which of the following water sources does your household use on a regular basis for any part of the year for any purpose?**<br><br>*Read out all types and check all that are used.*	Piped Water<br>Piped into dwelling/indoor.. .... 1/0<br>Pipe to yard/plot………….. ... 1/0<br>Public tap/standpipe ………1/0<br>Tube well or borehole ... ……………1/0<br>Dug Well<br>Protected Well ... ……………..1/0<br>Unprotected Well . ……………1/0<br>Water from Spring<br>Protected Spring .... …………..1/0<br>Unprotected Spring .. ………….1/0<br>Rainwater …………………………1/0<br>Tanker Truck ………………………1/0<br>Cart or Bicycle with Small Tank .. …1/0<br>Surface water<br>(River / Dam / Lake / Pond / Stream<br>/ Canal / Irrigation Channel)... ……..1/0<br>Bottled Water ……………………...1/0<br>Sachet Water . ………………………1/0<br>No Response________________________-99	

For peer review only - http://bmjopen.bmj.com/site/about/guidelines.xhtml

111b	**What is the main source of water used by your household to take shower?**<br>*Read out Q110a selections only.*	Piped Water<br>Piped into dwelling/indoor.. .... 1/0<br>Pipe to yard/plot………….. ... 1/0<br>Public tap/standpipe ……….1/0<br>Tube well or borehole .. …………….1/0<br>Dug Well<br>Protected Well . ……………….1/0<br>Unprotected Well …………….1/0<br>Water from Spring<br>Protected Spring .. …………….1/0<br>Unprotected Spring . …………..1/0<br>Rainwater …………………………..1/0<br>Tanker Truck ………………………1/0<br>Cart or Bicycle with Small Tank . ….1/0<br>Surface water<br>(River / Dam / Lake / Pond / Stream<br>/ Canal / Irrigation Channel)... ……..1/0<br>Bottled Water ……………………...1/0<br>Sachet Water . ………………………1/0<br>No Response________________________-99	

For peer review only - http://bmjopen.bmj.com/site/about/guidelines.xhtml

111c	**What is the main source of water used by your household to wash cloths?**<br>*Read out 110b selections only.*	Piped Water<br>Piped into dwelling/indoor.. .... 1/0<br>Pipe to yard/plot………….. ... 1/0<br>Public tap/standpipe ………1/0<br>Tube well or borehole ... ……………1/0<br>Dug Well<br>Protected Well ... ……………..1/0<br>Unprotected Well . ……………1/0<br>Water from Spring<br>Protected Spring .... …………..1/0<br>Unprotected Spring .. ………….1/0<br>Rainwater …………………………1/0<br>Tanker Truck ………………………1/0<br>Cart or Bicycle with Small Tank .. …1/0<br>Surface water<br>(River / Dam / Lake / Pond / Stream<br>/ Canal / Irrigation Channel)... ……..1/0<br>Bottled Water ……………………...1/0<br>Sachet Water . ………………………1/0<br>No Response……………………….-99	
111d	**How long does it take to go to [MAIN WATER SOURCE]get water, and come back?**<br>*Zero is a possible answer*<br>*Enter -88 for do not know*<br>*Enter -99 for no response*<br><br>*Report the time into minutes.*<br>*Answer includes waiting time in line.*	Minutes: [ ]	

For peer review only

SECTION-1 HOUSEHOLD CHARACTERISTICS

Section 1. 3 – Household Observation

To fill the next part of the questionnaire I need to actually observe what materials you have used to build the floor, wall and roof of your house. I also wish to take a look at the place where members of the household wash their hands. Is it ok if I take a walk with you in the compound and do the observations?

112	**Main material of the floor**<br>*Observe.*	Earth/Sand ....1<br>Dung ....2<br>Wood Planks....................................3<br>Palm/Bamboo ....4<br>Polished wood ....5<br>Asphalt strips....................................6<br>Ceramic Tiles ....7<br>Cement ....8<br>Carpet ....9<br>Other________________________<br>Don't know…………………………-88	
113	**Main material of the roof**<br>*Observe.*	No Roof ....1<br>Thatch/Leaf/ Mud....................................2<br>Rustic Mat/Plastic Sheets ....3<br>Bamboo ....4<br>Wood Planks....................................5<br>Cardboard ....6<br>Corrugated Iron/Metal ....7<br>Asbestos/Cement Fiber..............................8<br>Cement/Concrete ....9<br>Roof Shingles ....10<br>Others________________________<br>Don't know…………………………-88	

For peer review only

For peer review only - http://bmjopen.bmj.com/site/about/guidelines.xhtml

114	**Main material of the exterior walls**<br>*Observe.*	No Walls..1<br>Trunks/Bamboo ....2<br>Dirt.……………………………….....3<br>Wood with Mud...4<br>Stone with Mud ....5<br>Uncovered Adobe.......................................6<br>Plywood..7<br>Cardboard ....8<br>Reused Wood ....9<br>Corrugated sheets..………………10<br>Cement ....11<br>Stone with Lime/Cement..........................12<br>Bricks ....13<br>Cement Blocks ....14<br>Covered Adobe...15<br>Wood Planks/Shingles..............................16<br>Others___________________________<br>Don’t know…………………………-88	
115a	**We would like to learn about the places that households use to wash their hands. Can you please show me where members of your household most often wash their hands?**	Observed, fixed place ....……………….1<br>Observed, mobile.......................................2<br>Not observed, not in yard/plot ....3<br>Not observed, no permission to see ....4<br>Not observed, other reason ....5<br>No response .... -99	
115b	**At the place where the household washes their hands, observe if:**<br>*Check all that apply.*	Soap is present..1/0<br>Stored water is present ....1/0<br>Running water is present ....1/0<br>None of the above.................................. -77	

For peer review only - http://bmjopen.bmj.com/site/about/guidelines.xhtml

SECTION – 2 SCABIES AT A HOUSEHOLD LEVEL

Now I am going to ask you about skin complaints, whether you have seen or heard any of your household members complaining [small or medium fluid containing or solid itchy or non-itchy rashes with a burrow connecting the bumps and mainly affecting the finger webs, the wrist, back of the knee, around the ankle or beneath the breast or the buttock].

[→ Show picture set no 1]

201	**Have you seen or heard any of your household members complaining [***Repeat the manifestations indicated in the preamble***]?**	Yes……………………………………1<br>No……………………………………..0<br>Don't know…………………………-88<br>No response………………………-99	0 → 205
202	**How many of the household members exhibited [***Repeat the manifestations indicated in the preamble***]?**	Number_______	
203	**Which members of the household presented [***Repeat the manifestations indicated in the preamble***]?**<br>*Take name/code of members of the household from section 1(Household roster)*	1.<br>2.<br>3.<br>4.<br>5.	
204	**What signs have you observed on their skin [→ Show picture set no 2]?**<br>*This question is about any of the household members*<br>*More than one answer is possible*	Vesicles.……………………………….1<br>Papules ……………………………….2<br>Pustules ..……………………………..3<br>Bumps……………………………………4<br>Scabies burrows……………………..5<br>Crust…………………………………...6<br>Scratch……………………………….7<br>Others________________________<br>Don't know…………………………-88<br>No response……………………….-99	

For peer review only - http://bmjopen.bmj.com/site/about/guidelines.xhtml

**Next, I will ask you few questions about health service availability and utilization**			
205	**What is the nearest health facility to your home?**	Health post………………………….1<br>Health center……………………….2<br>Private clinic………………………..3<br>Hospital..…………………………...4<br>Don't know……………………….-88<br>No response…………………….-99	
206	**Where do you usually go if you are sick, or to treat a general health problem?**	Health post………………………….1<br>Health center……………………….2<br>Private clinic………………………..3<br>Hospital..…………………………...4<br>Traditional healers…………………5<br>Holly water………………………….6<br>Prayer……………………………….7<br>Don't go anywhere…………………8<br>Others______________________<br>Don't know……………………...-88<br>No response……………………...-99	
207	**How far do you live from the (***The facility selected in Q.no.205***)?**<br>*The response could be recorded either in KMS or the time it takes to get to the facility, walking on foot.*<br>*The time should be filled in minutes*	In KMS ☐<br>Time it takes if walked on foot ☐	

For peer review only - http://bmjopen.bmj.com/site/about/guidelines.xhtml

208	**How do you travel to (*The facility selected in Q.no.205*)?**	On foot……………………………1<br>On a horse back……………………2<br>On a chariot………………………..3<br>By car……………………………...4<br>By other motorized vehicles……….5<br>Others_____________________<br>Don't know………………………-88<br>No response..…………………….-99	

For peer review only - http://bmjopen.bmj.com/site/about/guidelines.xhtml

CHAPTER-2

INDIVIDUAL QUESTIONNAIRE FOR MEMBERS OF THE SELECTED HOUSEHOLD

HH number: ___________

Name of HH head :____________

Code HH member :____________

SECTION 3 – SCABIES MANIFESTATIONS ON INDIVIDUAL PARTICIPANTS

I am going to ask you if you have skin complaints, such as itch and rash, we ask these questions to all participants in order to understand the occurrence of the skin condition that we are interested to know about. For some of the questions you can simply tell me whether you have the symptoms, but for some I will ask you to show me parts of your body affected by the condition. I hope it is ok, but if you feel uncomfortable to show me your body you can tell me and we can skip the part which asks to show me your skin.

NB: All members of the household will be asked questions 301-310

301	**Relationship of the respondent with the participant of the study?**<br>*This question will be asked only if the participant is a minor*	Parent……………………………..1<br>Care taker…………………………2<br>Relative……………………………3<br>Others_______________________<br>Don't know………………………-88<br>No response……………………..-99	
302	**Do you feel itch on any part of your body?**	Yes…………………………………1<br>No…………………………………..0<br>Don't know………………………-88<br>No response……………………-99	0 → 305
303	**How do you grade the itch?**	Mild………………………………1<br>Moderate…………………………..2<br>Intense……………………………..3<br>Don't know………………………-88<br>No response..……………………-99	

304	**On which part of your body do you mainly feel the itch?**<br>*More than one response is possible*<br>*Please help the respondent use the body map*	The wrist……………………………1<br>Inter digital space……………………2<br>The elbow……………………………..3<br>Abdomen………………………………4<br>Back of the knee……………………...5<br>Ankle area……………………………..6<br>The head………………………………7<br>The neck area……..…………………8<br>Genital area…………………………...9<br>The buttock……………………………10<br>Others________________________<br>Don't know…………………………-88<br>No response……………………..-99	
305	**Do you have a skin rash on any part of your body?**	Yes…………………………………1<br>No……………………………………..0<br>Don't know…………………………-88<br>No response..………………………-99	0 → 401
306	**Is the rash associated with an itchy sensation?**	Yes…………………………………1<br>No……………………………………..0<br>Don't know…………………………-88<br>No response..………………………-99	

For peer review only - http://bmjopen.bmj.com/site/about/guidelines.xhtml

307	**Which part of your body does the rash mainly involve?**<br>*More than one response is possible*	The wrist……………………………….1<br>Inter digital space.…………………….2<br>The elbow……………………………..3<br>Abdomen……………………………….4<br>Back of the knee.……………………...5<br>Ankle area……………………………..6<br>The head……………………………….7<br>The neck area.……..………………….8<br>Genital area…………………………...9<br>The buttock..…………………………10<br>Others________________________<br>Don't know………………………….-88<br>No response..………………………..-99			
308	**Can you show me parts of your body affected by the skin rash?**<br>*Observe and select parts of the body affected by the rash*<br>*More than one body part can be checked*		Yes	No	
		The wrist<br>Inter digital space<br>The elbow<br>Abdomen<br>Back of the knee<br>Ankle area<br>The head<br>The neck area			
		The below two body parts could be documented based on history			
		Genital area<br>The buttock			
		Others________________________<br>Don't know………………………….-88<br>No response……………………….-99			

For peer review only

309	**Characterize the rash you have observed on the respondent?**<br>*This question is for the data collector*	Vesicles……………………………….1<br>Papules ……………………………….2<br>Pustules ..…………………………….3<br>Bumps……………………………….4<br>Scabies burrows……………………….5<br>Crust……………………………….....6<br>Scratch……………………………….7<br>Others_______________________<br>Don’t know…………………………-88<br>No response..………………………..-99	
310	**How many lesions were observed on the patient.**	≤10………………………………….1<br>10-50……………………………….2<br>≥ 50………………………………..3	

SECTION 4 – SCABIES TRANSMISSION

The skin condition which we are interested to know about has manifestations like; [*small or medium fluid containing or solid itchy or non-itchy rashes with a burrow connecting the bumps mainly affecting the finger webs, on the wrist, back of the knee, around the ankle or beneath the breast or the buttock*]. Since one of the major mode of transmission of this condition is a close physical contact with the infected person, knowing all about the possibilities which invites close physical contact is pretty important for us to understand the disease transmission.

The physical contact should be [*In the past two months]*

[→ Show picture no 1]

NB: Only adults will respond to questions 401-403

401	**Do you know anyone presenting the symptoms [**Read the manifestations indicated in the preamble**]?**	Yes…………………………………….1<br>No...…………………………………..0<br>No response..………………………-99<br>Don’t know…………………………-88	0 → 501

When answering questions 402a-c please think of three people, with the mentioned manifestations, whom are closer to you in comparison to the others with manifestations. We will refer to these people as person 1-3.

For peer review only - http://bmjopen.bmj.com/site/about/guidelines.xhtml

402a	**How do you know person 1?**	Friend…………………………….......1<br>Neighbour…………………………….2<br>Business associate…………………....3<br>From the market place..……………...4<br>From school………………………….5<br>On a public/private transport...……....6<br>Spouse…………………………….....7<br>Sexual partner..……………….........8<br>Other members of the household……9<br>Others____________________<br>No response………………………-99<br>Don't know………………………-88	
402b	**How do you know person 2?**	Friend…………………………….......1<br>Neighbor……………………………..2<br>Business associate…………………....3<br>From the market place..……………...4<br>From school………………………….5<br>On a public/private transport..……....6<br>Spouse…………………………….....7<br>Sexual partner..……………….........8<br>Other members of the household……9<br>Others____________________<br>No response………………………-99<br>Don't know…………………………-88	

For peer review only - http://bmjopen.bmj.com/site/about/guidelines.xhtml

402c	**How do you know person 3?**	Friend……………………………......1<br>Neighbor……………………………..2<br>Business associate…………………....3<br>From the market place..……………...4<br>From school…………………………5<br>On a public/private transport...……....6<br>Spouse…………………………….....7<br>Sexual partner..……………….........8<br>Other members of the household……9<br>Others_______________________<br>No response..……………………….-99<br>Don't know…………………………-88	
When answering questions **403a-c**, please think of the type of contact you had with each one of the three people, you have referred to in questions 402a-c.			
403a	**Which one of the listed encounters did you have with person 1?**<br>*The encounter should be a minimum of one time.*<br>*More than one answer is possible.*<br>*Categories **7 and 8** will only be mentioned as possible responses if the reported contacts are spouses, sexual partners or household members.*	Sat together...……………….………...1<br>Shook hands with.....…………………2<br>Dine together...…..……………………3<br>Shared work related materials….…….4<br>Shared utensils…..……………………5<br>Shared cloths…..……………………......6<br>*Shared a bed/resting space… ….…..….7*<br>*Took shower together..… … … … … ....8*<br>Others_____________________<br>Don't know…...……………………….-88<br>No response..…...…………………..-99	

For peer review only - http://bmjopen.bmj.com/site/about/guidelines.xhtml

403b	**Which one of the listed encounters did you have with person 2?**<br>*The encounter should be a minimum of one time.*<br>*More than one answer is possible.*<br>*Categories **7 and 8** will only be mentioned as possible responses if the reported contacts are spouses, sexual partners or household members.*	Sat together...……………….………...1<br>Shook hands with....…………………2<br>Dine together...….…………………….3<br>Shared work related materials..………4<br>Shared utensils….…………………….5<br>Shared cloths…..…………………....6<br>*Shared a bed/resting space… ….....…...7*<br>*Took shower together…..… … … … ….8*<br>Others______________________<br>Don't know…...…………………….-88<br>No response…...…………………..-99			
403c	**Which one of the listed encounters did you have with person 3?**<br>*The encounter should be a minimum of one time.*<br>*More than one answer is possible.*<br>*Categories **7 and 8** will only be mentioned as possible responses if the reported contacts are spouses, sexual partners or household members.*	Sat together...……………….………...1<br>Shook hands with....…………………2<br>Dine together...….…………………….3<br>Shared work related materials..………4<br>Shared utensils….…………………….5<br>Shared cloths…..…………………....6<br>*Shared a bed/resting space… ….....…...7*<br>*Took shower together…..… … … … ….8*<br>Others______________________<br>Don't know…...…………………….-88<br>No response...…...……………………..-99			
**SECTION-5 SUMMARY OF THE REPORTED MANIFESTATIONS**<br>**This part summarizes the reported symptoms and observed signs on the skin of the participant. This table is to be filled only by the provider, and at the end of this section the HEW will report her impression of the participants condition.**					
	**Main criteria**	**Yes**	**No**	**Not sure**	
501	Scabies burrows				
502	Typical lesions affecting male genitalia				
503	Typical lesions in a typical distribution				

For peer review only

504	Atypical lesions or atypical distribution				
	History features				
505	Itch				
506	Close contact with an individual who has itch or typical lesions in a typical distribution				
507	**What is the impression of the health extension worker based on the above information?**	Clinical scabies……………………….1<br>Suspected scabies…………………..2<br>Other skin diseases…………………3<br>Others_______________________<br>Don't know………………………….-88<br>No response………………………..-99			
508	**Remarks on the diagnosis from the provider**				
**<u>SECTION-6 HEATH SEEKING</u>**<br>**Based on the symptoms you told me before I think you might probably have an infestation called scabies and we will reach at a confirmatory diagnosis after we discuss with the skin doctors at the health center. But up until then I would like to ask you few questions about your condition.**<br>***NB: Only participants with clinical scabies will be asked questions 601-606***					
601	**What was the first manifestation of the disease?**	Itch………………………………….1<br>Rash………………………………….2<br>Papules……………………………….3<br>Nodules……………………………….4<br>Ulcer…………………………………..5<br>Crust…………………………………..6<br>Don't know………………………….-88<br>No response……………………….-99			
602	**For how long did you stay with the disease counting from the day you experienced the [**The response to Q.no 601**]?**<br>*Assist the respondent to remember the day when he/she had the first manifestation*	Number of days ☐			

For peer review only - http://bmjopen.bmj.com/site/about/guidelines.xhtml

603	**Have you done anything to treat the symptoms?**	Yes…………………………………1<br>No…………………………………..0<br>Don't know………………………-88<br>No response……………………-99	0 → 701
604	**What did you do for the first time to get rid of the symptoms?**<br>*This question pertains to both modern and traditional health care services*	Home remedy………………………1<br>Traditional medicine………………2<br>Holly water…………………………3<br>Prayer………………………………4<br>Health care from a health post……...5<br>Health care from a health center……6<br>Health care from private clinic.……7<br>Health care from hospital…………8<br>Others______________________<br>Don't know………………………..-88<br>No response…..…………………...-99	If 1,2,3,4 → 701
605	**How many days after the onset of the first symptom did you visit [**Health care facility from Q.no.604]**?**	Number of days ☐	

For peer review only

606	**Why didn't you visit** [The facility from Q.no.604] **earlier?**	Not sure where to go……………..……1<br>The symptoms were not serious……2<br>Could recover on its own…………..…3<br>I didn't have time…………………..4<br>Fear of stigma……………………...5<br>Didn't think there is a medicine for it……………………………………..6<br>Distance/lack of transport to go to the health facility……....................................7<br>Fear of high treatment cost………..8<br>Others______________________<br>Don't know……………………….-88<br>No response…………………........-99	
**SECTION- 7- DERMATOLOGIC QUALITY OF LIFE QUESTIONS**<br>**The following questions are intended to measure the effect of your skin condition on your overall quality of life. The responses are organized in a way which reflects the degree of severity of your skin condition on the indicated aspect of your life. So before you give your responses please take a moment to rate how severely it has affected you. The responses range from *not at all to very much*, please select the response which describes your condition appropriately.**<br>*NB: Questions 701-708 will only be directed to adult (≥18 years) participants.*			
701	Over the last week, how itchy, sore, painful or stinging has your skin been?	Very much…………………………..4<br>A lot………………………………….3<br>A little……………………………….2<br>Not at all..……………………………1	
702	Over the last week, how embarrassed or self conscious have you been because of your skin?	Very much…………………………..4<br>A lot………………………………….3<br>A little……………………………….2<br>Not at all..……………………………1	
703	Over the last week, how much has your skin interfered with you going shopping or looking after your home or garden?	Very much…………………………..4<br>A lot………………………………….3<br>A little……………………………….2<br>Not at all..……………………………1	

For peer review only - http://bmjopen.bmj.com/site/about/guidelines.xhtml

704	Over the last week, how much has your skin influenced the clothes you wear?	Very much……………………………4<br>A lot…………………………………3<br>A little………………………………2<br>Not at all……………………………1	
705	Over the last week, how much has your skin affected any social or leisure activities?	Very much……………………………4<br>A lot…………………………………3<br>A little………………………………2<br>Not at all……………………………1	
706	over the last week how much has your skin been a problem at work or studying?	Very much……………………………4<br>A lot…………………………………3<br>A little………………………………2<br>Not at all……………………………1	
707	Over the last week, how much has your skin created problems with your partner or any of your close friends or relatives	Very much……………………………4<br>A lot…………………………………3<br>A little………………………………2<br>Not at all……………………………1	
708	Over the last week, how much has your skin caused any sexual difficulties?	Very much……………………………4<br>A lot…………………………………3<br>A little………………………………2<br>Not at all……………………………1	
**SECTION-8 KNOWLEDGE ABOUT SCABIES**<br>**I will ask you few questions about your knowledge about scabies. You will just tell me what you know about the disease, you are expected to tell me what you know about the disease. It doesn't need to be the right answer.**<br>**NB: Questions 801-810 are for adult (>18 years of age) study participants.**			
801	**Have you ever heard about scabies?**	Yes…………………………………1<br>No…………………………………..0<br>Don't know………………………-88<br>No response……………………-99	0 → 1001

For peer review only - http://bmjopen.bmj.com/site/about/guidelines.xhtml

802	**Where did you first learn about scabies?**<br>*Check all that are mentioned*	Newspapers and magazines……………..1<br>Radio……………………………………2<br>TV………………………………………3<br>Billboards………………………………4<br>Brochures, posters and other printed materials……………………………….5<br>Health extension workers………………6<br>Health workers…………………………7<br>Family, friends, neighbors and colleagues………………………………8<br>Religious leaders……………………….9<br>Teachers………………………………10<br>Others_______________________<br>Don't know………………………….-88<br>No response……………………........-99	
803	**What are the signs and symptoms of scabies?**<br>*Check all that are mentioned*<br>*Don't read the choices to the respondent*	Itch…………………………………1<br>Rash/papules……………………..2<br>Ulceration of the skin……………3<br>Skin crust…………………………4<br>Others____________________<br>Don't know………………………..-88<br>No response……………………...-99	

For peer review only

804	**How can a person get scabies?**<br>*Check all that are mentioned*<br>*Don't read the choices to the respondent*	Prolonged physical contact with a patient…………………………….………1<br>Sharing clothes with the infected person……..…………………………2<br>Sharing bedding with the infected person…..……………………………3<br>Through droplets when coughing….4<br>Through blood contact………………5<br>Due to evil spirit………………………6<br>From the soil…………………………7<br>From contaminated water…………..8<br>Others_____________________<br>Don't know..………………………..-88<br>No response..……………………...-99	
805	**Can a person with scabies be cured?**	Yes………………………………1<br>No…………………………………..0<br>Don't know………………………..-88<br>No response……………………...-99	
806	**In your opinion who can catch scabies?**<br>*Check all that are mentioned*<br>*Don't read the choices to the respondent*	Anybody……………………………..1<br>Poor people…………………………2<br>Rich people…………………………3<br>Homeless people…………………...4<br>People from the city………………...5<br>Farmers……………………………...6<br>Commercial sex workers…………..7<br>Prisoners……………………………8<br>Others_______________________<br>Don't know………………………..-88<br>No response……………………...-99	

For peer review only - http://bmjopen.bmj.com/site/about/guidelines.xhtml

807	**How can someone with scabies get cured?**	With therapeutic cream or tablets…..1<br>With traditional healers………………2<br>With holly water……………………….3<br>With prayer……………………………4<br>Others_______________________<br>Don't know………………………….-88<br>No response…………………….....-99	
808	**Do you feel you are well informed about scabies?**	Yes……………………………………1<br>No…………………………………..0<br>Don't know……………………….-88<br>No response……………………...-99	
809	**Do you wish to get more information about scabies?**	Yes……………………………………1<br>No…………………………………..0<br>Don't know……………………….-88<br>No response……………………...-99	

For peer review only

810	**Which medium can best reach people like you, who are in need of information on scabies?**	Newspapers and magazines………1<br>Radio…………………………………2<br>TV……………………………………3<br>Billboards……………………………4<br>Brochures, posters and other printed materials……………………………..5<br>Health extension workers…………6<br>Health workers………………………7<br>Family, friends, neighbors and colleagues……………………………8<br>Religious leaders……………………9<br>Teachers……………………………..10<br>Other________________________<br>Don't know………………………..-88<br>No response……………………...-99	
**SECTION-9 ATTITUDE TOWARDS SCABIES**<br>**The following few questions are intended to assess your attitude towards scabies**<br>***NB: Questions 801- 807 will be asked for scabies free adults (Aged ≥ 18 years)***			
901	**Do you think you can get scabies?**	Yes…………………………………1<br>No…………………………………..2<br>Don't know………………………..-88<br>No response……………………...-99	
902a	**Why do you think you can catch scabies?**	Reason______________________________<br>________________________	
902b	**Why do you think you don't catch scabies?**	Reason______________________________<br>________________________	

For peer review only - http://bmjopen.bmj.com/site/about/guidelines.xhtml

903	**What would you feel if you found out that you have scabies?**<br>*Check all that are mentioned*<br>*Don't read the choices to the respondent*	Nothing……………………………1<br>Fear………………………………..2<br>Shame……………………………..3<br>Surprise……………………………4<br>Sadness……………………………5<br>Others_______________________<br>Don't know………………………..-88<br>No response……………………...-99	
904a	**What would you do if you think you had scabies?**	Go to a health facility………………..1<br>Wait till it goes away on its own……2<br>Seek self-treatment options………...3<br>Go to traditional healers…………….4<br>Go to a pharmacy……………………5<br>Others_______________________<br>Don't know………………………..-88<br>No response……………………...-99	
904b	**Why wouldn't you want to go to a health facility?**<br>*Check all that are mentioned*<br>*Don't read the choices to the respondent*	Not sure where to go…………………1<br>The symptoms were not serious……2<br>Could recover on its own…………….3<br>I didn't have time……………………..4<br>Fear of stigma………………………...5<br>Didn't think there is a medicine for it..6<br>Distance/lack of transport to go to the health facility....................................7<br>Fear of high treatment cost………....8<br>Others_______________________<br>Don't know…………………………-88<br>No response……………………....-99	

For peer review only - http://bmjopen.bmj.com/site/about/guidelines.xhtml

905	**How expensive do you think the care for scabies at health care facilities?**<br>*Check only one response*	Reasonable price……………………1<br>Moderately expensive..……………..2<br>Very expensive………………………3<br>Free of charge………………………..4<br>Don't know………………………….-88<br>No response……………………......-99	
906a	**Do you know people who have/had scabies?**	Yes……………………………………1<br>No……………………………………..2	
906b	**Which of the following statements best describe your feeling towards scabies patients?**<br>*Check only one response*	I don't feel anything……………………1<br>It is their problem I don't get scabies..2<br>I feel compassion and want to help....3<br>I feel compassion but prefer to stay away……………………………………4<br>I fear they may infect me……………5<br>Others_______________________<br>Don't know………………………….-88<br>No response……………………......-99	
907	**In your community how does a person with scabies get treated?**<br>*Check only one response*	Most people prefer to reject him/her..1<br>People appear friendly but they prefer to reject him/her……………………2<br>They get help/support………………..3<br>Others_______________________<br>Don't know………………………….-88<br>No response……………………......-99	

SECTION 10 – PERSONAL HYGIENE AND ONCHO MDA RELATED QUESTIONS

Now I would like to ask you a few questions about water, sanitation and hygiene

NB: All participants will be asked questions 1001-1010.

For peer review only - http://bmjopen.bmj.com/site/about/guidelines.xhtml

1001	**How often do you take shower?**	Number of times:<br>In a week ___<br>In a month ___<br>In a year ___	
1002	**Do you use soap or other detergents when you take shower?**	Yes, always……………………………1<br>Yes, sometimes...…………………..2<br>No………………………………….....3<br>Others_____________________<br>No response……………………….-88	
1003	**How often do you wash your cloths?**	In a week ___<br>In a month ___<br>In a year ___	
1004	**Do you use soap or other detergents when you wash your cloths?**	Yes, always……………………………1<br>Yes, sometimes..…………………..2<br>No……………………………………3<br>Others_____________________<br>No response……………………….-88	
**Questions 1005-1010 are about onchocerchiasis MDA given in your kebele, so you will answer the following questions based on your past experience.**			
1005	**Have you ever received a medication to treat or prevent onchocerciasis?**	Yes……………………………………1<br>No ………………………………..0<br>Don't know…...…………………….-88<br>No response…..…………………..-99	0→END
1006	**How many times have you received a medication to treat or prevent onchocerciasis?**	Number ☐	
1007	**When was the last time you received a medication to treat or prevent onchocerciasis?**	Day___Month____Year_____	

For peer review only

1008	**Did you take the medication you received the last time?**	Yes……………………………………1<br>No……………………………………..0<br>Don't know…………………………-88<br>No response…..…………………..-99	1 → 1010
1009	**Why didn't you take the medication?**	It makes me itch……………………..1<br>Not good for my health……………..2<br>I was fasting…………………………..3<br>My friends/family told me not to…….4<br>Others________________________<br>Don't know…………………………-88<br>No response…..…………………..-99	
1010	**Where did you get the medication from?**	Health center…………………………1<br>Hospital………………………………2<br>HEWs…………………………………3<br>HDA…………………………………...4<br>Others________________________<br>Don't know…………………………-88<br>No response………………………-99	
**END**	**I have completed the interview thank you for your time.**		

For peer review only - http://bmjopen.bmj.com/site/about/guidelines.xhtml

Table S2. Fixed asset variables used to categorise households into wealth quintiles.

Household asset and infrastructure related variables
Does your house have electricity?
Does your household own a clock?
Does your household own a radio?
Does your household own a television?
Does any member of your household own a mobile phone?
Does your household own a refrigerator?
Does your household own a table?
Does your household own chair?
Does your household own bed with cotton or sponge mattress?
Does your household own an electric hot-plate?
Does your household own a kerosene lamp?
Does any member of your household own a bicycle?
Does any member of your household own a motorcycle?
Does your household own animal drawn cart?
Does your household own a car?
Does your household own farm animals?
Does your household own piped water into the dwelling?
Does your household own piped water into the yard?
Does your household have access to public tap?
Does your household own a dug well?
Does your household use spring water?
Does your household use rainwater?
Does your household use water distributed using tanker trucks?
Is the floor of your house made of sand or earth or sand?
Is the floor of your house made of ceramic tiles?

For peer review only

For peer review only - http://bmjopen.bmj.com/site/about/guidelines.xhtml

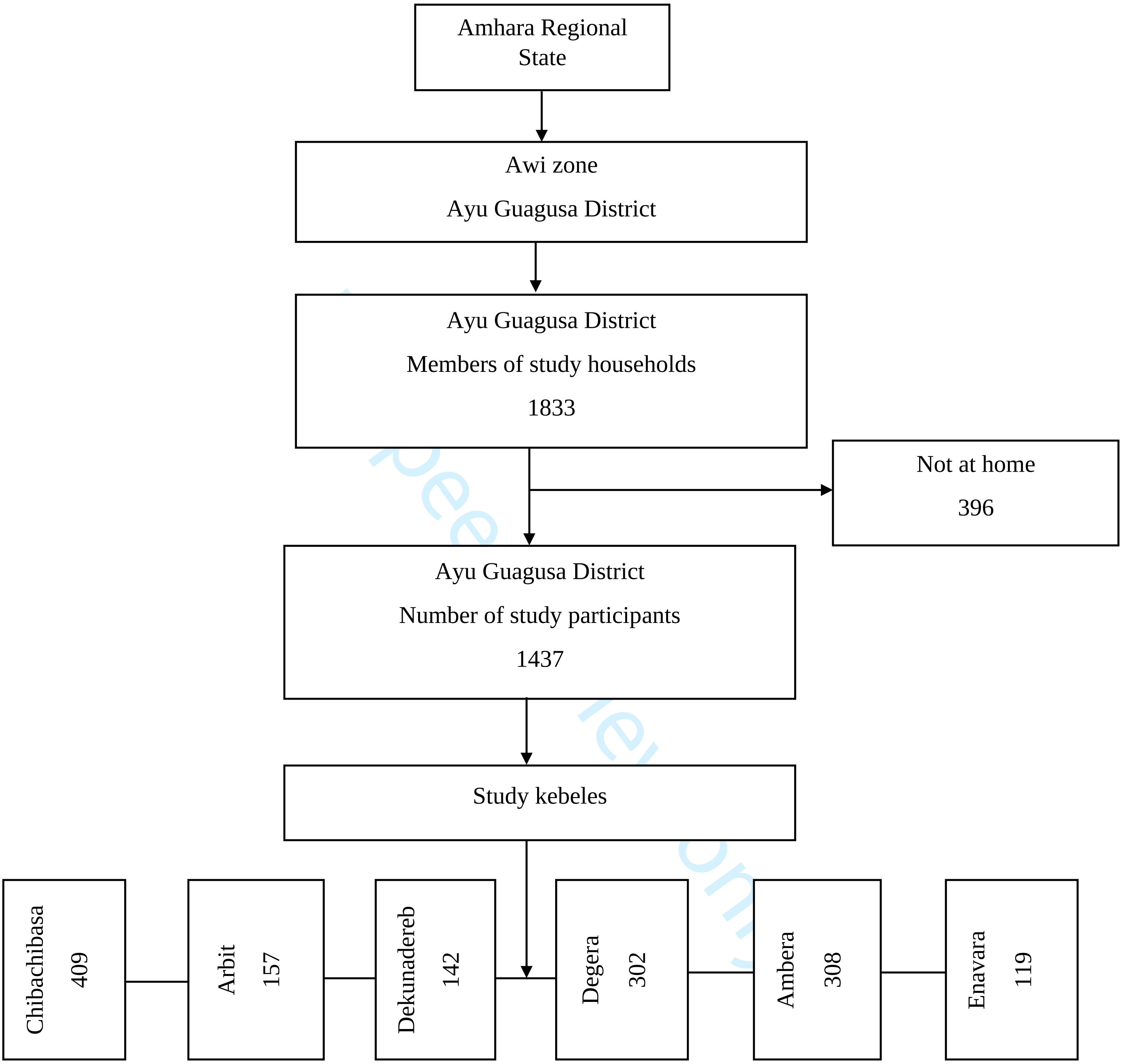

Figure S2. Study diagram, number of participants, and place of residence (*kebeles*)

For peer review only - http://bmjopen.bmj.com/site/about/guidelines.xhtml

Table S3 Distribution of participants by age and sex (n=1437)

Sex	Age categories	Total (%) (n=1437)	No scabies (%) (n=1245)	Clinical scabies (%) (n=192)
**Male**	0–10	173 (27.0)	148 (27.5)	25 (24.8)
	11–18	170 (26.6)	134 (24.9)	36 (35.6)
	19–40	165 (25.8)	144 (26.7)	21 (20.8)
	>41	132 (20.6)	113 (20.9)	19 (18.8)
**Female**	0–10	192 (24.1)	168 (23.8)	24 (26.4)
	11–18	176 (22.1)	153 (21.7)	23 (25.3)
	19–40	270 (33.9)	243 (34.4)	27 (29.7)
	>41	159 (19.9)	142 (20.1)	17 (18.7)

For peer review only

Table S4 Scabies manifestations among clinically diagnosed cases (n=192)

Variable	Categories	n (%) *
**Skin lesions**	Vesicles	130 (67.7)
	Scratch marks	117 (60.9)
	Papules	99 (51.6)
	Skin crust	78 (40.6)
	Pustule	52 (27.1)
	Burrows	8 (4.2)
**Skin rash distribution**	Trunk	119 (62.0)
	Elbow	99 (51.6)
	Finger webs	98 (51.0)
	Wrist	81 (42.2)
	Knee	38 (19.8)
	Neck	25 (13.0)
	Ankle	12 (6.3)
	Head	7 (3.6)
**History features**	Itch	184 (95.8)
	Contact history with a presumed scabies patient	184 (95.8)
**Relationship of participant with participant presumed source of infestation (n=184) #**	Household members	146 (79.3)
	Neighbour	20 (10.9)
	Friend	15 (8.2)

*Cumulative scores for some variables were more than the number of participants as the items were multi select.

Calculated out of the number of participants who reported contact history with a person exhibiting signs of scabies.

For peer review only

Table S5 Care seeking characteristics of participants who sought care for scabies suggestive symptoms (n=62)

Variable	Categories	n (%)
**Source of care**	Health facility	40 (64.5)
	Self-treatment	14 (22.6)
	Holy water	3 (4.8)
	Prayer	1 (1.6)
	Missing values	4 (6.5)
**Time to care seeking**	Median number of days (IQR)	20 (7–30)

For peer review only

For peer review only - http://bmjopen.bmj.com/site/about/guidelines.xhtml

STROBE Statement—Checklist of items that should be included in reports of ***cross-sectional studies***

	Item No	Recommendation	Page No
**Title and abstract**	1	(*a*) Indicate the study's design with a commonly used term in the title or the abstract	Pages 1-3
		(*b*) Provide in the abstract an informative and balanced summary of what was done and what was found	2
**Introduction**			
Background/rationale	2	Explain the scientific background and rationale for the investigation being reported	4-5
Objectives	3	State specific objectives, including any prespecified hypotheses	5
**Methods**			
Study design	4	Present key elements of study design early in the paper	5
Setting	5	Describe the setting, locations, and relevant dates, including periods of recruitment, exposure, follow-up, and data collection	5
Participants	6	(*a*) Give the eligibility criteria, and the sources and methods of selection of participants	6
Variables	7	Clearly define all outcomes, exposures, predictors, potential confounders, and effect modifiers. Give diagnostic criteria, if applicable	7
Data sources/ measurement	8*	For each variable of interest, give sources of data and details of methods of assessment (measurement). Describe comparability of assessment methods if there is more than one group	6
Bias	9	Describe any efforts to address potential sources of bias	6
Study size	10	Explain how the study size was arrived at	6
Quantitative variables	11	Explain how quantitative variables were handled in the analyses. If applicable, describe which groupings were chosen and why	7
Statistical methods	12	(*a*) Describe all statistical methods, including those used to control for confounding	7
		(*b*) Describe any methods used to examine subgroups and interactions	7
		(*c*) Explain how missing data were addressed	
		(*d*) If applicable, describe analytical methods taking account of sampling strategy	7
		(*e*) Describe any sensitivity analyses	
**Results**			
Participants	13*	(a) Report numbers of individuals at each stage of study—eg numbers potentially eligible, examined for eligibility, confirmed eligible, included in the study, completing follow-up, and analysed	8
		(b) Give reasons for non-participation at each stage	8
		(c) Consider use of a flow diagram	8
Descriptive data	14*	(a) Give characteristics of study participants (eg demographic, clinical, social) and information on exposures and potential confounders	Pages 8&9
		(b) Indicate number of participants with missing data for each variable of interest	Tables 1, 2 & 3.
Outcome data	15*	Report numbers of outcome events or summary measures	9
Main results	16	(*a*) Give unadjusted estimates and, if applicable, confounder-adjusted estimates and their precision (eg, 95% confidence interval). Make clear which confounders were adjusted for and why they were included	10

For peer review only

		(*b*) Report category boundaries when continuous variables were categorized	8
		(*c*) If relevant, consider translating estimates of relative risk into absolute risk for a meaningful time period	8 & 9
Other analyses	17	Report other analyses done—eg analyses of subgroups and interactions, and sensitivity analyses	
**Discussion**			
Key results	18	Summarise key results with reference to study objectives	11
Limitations	19	Discuss limitations of the study, taking into account sources of potential bias or imprecision. Discuss both direction and magnitude of any potential bias	3
Interpretation	20	Give a cautious overall interpretation of results considering objectives, limitations, multiplicity of analyses, results from similar studies, and other relevant evidence	Pages 11-13
Generalisability	21	Discuss the generalisability (external validity) of the study results	11
**Other information**			
Funding	22	Give the source of funding and the role of the funders for the present study and, if applicable, for the original study on which the present article is based	14

*Give information separately for exposed and unexposed groups.

Note: An Explanation and Elaboration article discusses each checklist item and gives methodological background and published examples of transparent reporting. The STROBE checklist is best used in conjunction with this article (freely available on the Web sites of PLoS Medicine at http://www.plosmedicine.org/, Annals of Internal Medicine at http://www.annals.org/, and Epidemiology at http://www.epidem.com/). Information on the STROBE Initiative is available at www.strobe-statement.org.

For peer review only
